# Single-Arm, Non-randomized, Time Series, Single-Subject Study of Fecal Microbiota Transplantation in Multiple Sclerosis

**DOI:** 10.3389/fneur.2020.00978

**Published:** 2020-09-08

**Authors:** Phillip A. Engen, Antonia Zaferiou, Heather Rasmussen, Ankur Naqib, Stefan J. Green, Louis F. Fogg, Christopher B. Forsyth, Shohreh Raeisi, Bruce Hamaker, Ali Keshavarzian

**Affiliations:** ^1^Division of Gastroenterology, Department of Internal Medicine, Rush University Medical Center, Chicago, IL, United States; ^2^Department of Biomedical Engineering, Stevens Institute of Technology, Hoboken, NJ, United States; ^3^Department of Nutrition and Health Sciences, University of Nebraska, Lincoln, NE, United States; ^4^Genome Research Core, Research Resources Center, University of Illinois at Chicago, Chicago, IL, United States; ^5^Department of Biological Sciences, University of Illinois at Chicago, Chicago, IL, United States; ^6^Department of Community, Systems and Mental Health Nursing, College of Nursing, Rush University Medical Center, Chicago, IL, United States; ^7^Department of Food Science, Purdue University, West Lafayette, IN, United States; ^8^Department of Physiology, Rush University Medical Center, Chicago, IL, United States; ^9^Division of Pharmacology, Utrecht Institute for Pharmaceutical Sciences, Utrecht University, Utrecht, Netherlands

**Keywords:** multiple sclerosis, relapsing-remitting multiple sclerosis, fecal microbiota transplantation, microbiome, metabolomics, short-chain fatty acids, brain-derived neurotrophic factor, gait

## Abstract

Emerging evidence suggests intestinal microbiota as a central contributing factor to the pathogenesis of Relapsing-Remitting-Multiple-Sclerosis (RRMS). This novel RRMS study evaluated the impact of fecal-microbiota-transplantation (FMT) on a broad array of physiological/clinical outcomes using deep metagenome sequencing of fecal microbiome. FMT interventions were associated with increased abundances of putative beneficial stool bacteria and short-chain-fatty-acid metabolites, which were associated with increased/improved serum brain-derived-neurotrophic-factor levels and gait/walking metrics. This proof-of-concept single-subject longitudinal study provides evidence of potential importance of intestinal microbiota in the pathogenesis of MS, and scientific rationale to help design future randomized controlled trials assessing FMT in RRMS patients.

## Introduction

Multiple sclerosis (MS) is an autoimmune inflammatory demyelinating disease of the Central Nervous System (CNS) (1, 2). The pathological characteristics of MS is associated with the formation of demyelinating lesions primarily affecting the CNS white matter (1, 2). Focal lymphocytic infiltration is thought to be caused by the host's immune cells, including activated T cells and a significant contribution from B cells, accompanied by an abnormal blood brain barrier, which leads to the inflammatory/damaging lesions and plaques on the myelin and axons of the CNS (1, 2). Over time, damaged myelin causes impaired nerve signaling (axonal damage), leading to neurodegeneration and associated motor function degradation. MS is predominately in young adults (20–45 years of age) and affects twice as many women as men (3). Common symptoms include numbness, fatigue/weakness, visual impairment, gait, constipation, urinary bladder dysfunction, and depression (3). A brain MRI scan is the most useful test for confirming the diagnosis of MS (4). Currently, more than 2 million patients worldwide suffer from MS (5).

There are four clinical MS forms: relapsing-remitting-MS (RRMS), secondary-progressive-MS, primary-progressive-MS, and progressive-relapsing-MS (1, 2). Most common phenotype of MS (80% of cases) is RRMS, characterized by alternating periods of acute inflammation (relapses) and periods of relative clinical stability devoid of new neurological symptoms (remissions) (6). Inflammatory demyelinating focal white matter lesions dominate the pathology in acute MS and RRMS (1). As of 2020, the Federal Drug Administration (FDA) has approved multiple medications to suppress this immune/inflammatory cascade to prevent relapses, accumulation of lesions on magnetic resonance imaging (MRI), and disability progression. Yet, none of the medications have been shown to provide a cure for MS. With the cause of the disease still unknown, better understanding of the trigger for this immuno-inflammatory cascade is essential for developing more effective and novel therapeutic target(s) to inhibit progression of this debilitating disease.

Intestinal microbiome has been identified as a potential trigger modulating the host's immune and nervous system function in MS (7). Preclinical animal models of MS and cross-sectional observational studies in MS patients have reported altered intestinal (stool) microbiota communities (“dysbiosis”) which were associated with systemic inflammatory autoimmune responses of the host (8–11). Majority of MS intestinal dysbiotic microbiota data is characterized by decreases in the relative abundance of short-chain-fatty-acids (SCFA)-producing bacteria (e.g., *Faecalibacterium prausnitzii*) (11, 12). Elsewhere, prior studies demonstrated that low levels of SCFAs are associated with disruption of intestinal barrier and endotoxin leak (13–16). SCFAs, especially butyrate, enhance barrier function, and anti-inflammatory activities (17–19). Thus, a low SCFA-producing microbial community could mediate an inflammatory trigger in MS.

Accordingly, microbiota-directed interventions, like fecal-microbiota-transplantation (FMT), represent novel therapies to prevent MS flares and improve disease course (20, 21). Thus, we used an “n-of-1, longitudinal” design to assess whether FMT interventions can alter the MS microbiome, decrease inflammatory biomarkers, and improve MS symptoms in a subject with RRMS and severe gait problem.

## Methods and Materials

### Study Subject

A 48 year-old Caucasian male with active RRMS for 2 years, and complaints of difficulty in walking and bloating, agreed to provide biological samples and undergo repeated evaluation for 12-months before/after FMTs that he personally decided to undergo. Subject signed the Rush University Medical Center (RUMC) Institutional Review Board approved informed consent form (ORA# 18082009), and study was registered (ClinicalTrials.gov Identifier: NCT03975413). Subject underwent an FMT administered at Taymount Clinic-Bahamas Medical Center for treatment of MS disease, based on subject's own decision and not under medical direction by RUMC doctors. Additionally, the household spouse of the subject provided written informed consent to join the study. The spouse provided medical information and donated one stool sample to be used as an observational standard of control, for the fecal experiments. Demographic/clinical information (Supplementary Table 1).

### Study Design

This single-subject study is based on repeated observations within an individual over 12-months. Subject's stool and serum were collected at the following time points: before FMT (baseline: 0 week) followed by 3, 13, 26, 39, and 52 weeks. Two FMT interventions occurred during the year-long study. First FMT occurred between 0 and 3 weeks (Taymount Clinic); Second FMT occurred between 26 and 39 weeks (at-home booster) (Supplementary Figure 1). Sample collections are detailed in Supplementary Material.

### Study Procedures

The subject provided the following detailed Taymount Center protocol to RUMC to review. The Taymount FMT protocols and use of frozen implants, provided by the main Taymount Center in the United Kingdom, were implemented. Prior to the procedure, the subject provided the following test results to the Taymount Clinic in Nassau Bahamas: blood tests (SMAC-25, CBC, ESR, CRP, HIV, and acute viral hepatitis profiles); stool test (*Clostridium Difficile*, culture and sensitivity, and ova and parasite). These tests were normal/negative. Before the first FMT, standard bowel prep (MiraLAX powder) was conducted following Taymount Clinic protocol. The morning before the first FMT, no food or drink were consumed by the subject to keep the colon empty. All Taymount Clinic fecal preparations were from carefully pre-screened healthy stool donors chosen to be transplanted into the colon of the subject. Each FMT implant was from different donors and was numbered to ensure the subject would get a different donor for each day. This was done to provide a broader variety of microbiome over the multiple days of FMT implants.

The FMT implants were frozen liquid form (each vial ~2 total ounces of fluid with 84.5% water, 15% glycerol, and 0.5% sodium chloride), with very little if any solids. Each FMT implant was slowly thawed in its sealed vial in a water bath. When thawed, the FMT implant was combined with three vials of sodium chloride (Ph.Eur 0.9% saline Pod) in a 100 ml catheter tip syringe and the other 50 ml catheter tip syringe was filled with two vials of sodium chloride with a half pack of BiMuno probiotic (5 g sachet). The anus and the catheter were both coated with Vitamin A&B ointment for lubrication. With the subject lying sideways, the 18fg closed tip rectal catheter was inserted slowly and entirely up the colon. The FMT implant syringe was injected first, followed by the probiotic syringe injected second, to ensure the FMT went as far up the colon as possible. The rectal catheter was then removed with very little spillage. Immediately following this procedure, with the subject now lying face up, the stomach was rubbed up and to the left from the pelvis to the belly button for ~1 min. Then, the subject would lie on their right side for 10 min; then their left side for 10 min; and then on their back with legs lifted for 10 min. Finally, the subject would be remained seated upright for another 30 min. This completed the FMT implant procedure for that day. Furthermore, the subject was encouraged to refrain from making a bowel movement for as long as possible, but at least 2 h. The subject was able to achieve this.

In total, the subject underwent five consecutive days of FMT implants; followed by 2 days off; then another five consecutive days of FMT implants; and then left the Taymount Clinic in Nassau Bahamas to return home. The subject was later shipped multiple frozen stool boosters (stool received from United Kingdom Certified Stool Bank) to perform the FMT at home (similar procedural process as described above at the Taymount Clinic), excepted the subject performed boosters for 5 days vs. 10 days, as in the original Taymount Clinic Nassau Bahama's session. The subject was informed by the Taymount Clinic to perform the at-home frozen stool FMT boosters when their overall health began to regress due to their MS symptoms, and not based on a scheduled time frame. The subject was able to achieve this.

### Stool Microbiota Interrogation

Stool microbiota was assessed by non-targeted shotgun metagenome sequencing and taxonomic and functional gene profiling. Total DNA was extracted from fecal samples utilizing the FastDNA bead-beating Spin Kit for Soil (MP Biomedicals, Solon, OH, USA), and verified with fluorometric quantitation (Qubit, Life Technologies, Grand Island, NY). Library preparation was performed using the Celero DNA-Seq library preparation protocol (Nugen, Redwood City, CA) according to the manufacturer's instructions. Library was screened initially using an Illumina MiniSeq mid-output flow cell, and re-pooled and sequenced on an Illumina HiSeq4000 instrument. Library preparation was performed at the University of Illinois at Chicago Sequencing Core (UICSQC), and HiSeq4000 sequencing was performed by Novogene Corp (Chula Vista, CA).

Targeted SCFA metabolomics analysis of fecal samples was conducted by gas chromatography. Fecal targeted SCFA metabolite concentrations measured were acetate (mM/kg), propionate (mM/kg), butyrate (mM/kg), total SCFA (mM/kg), and total Butyrate-to-total SCFA ratio (mM/kg). Further methodological experimental details are in provided in the Supplementary Material.

### Serum Brain-Derived-Neurotrophic-Factor and Inflammatory Biomarkers

Serum Brain-Derived-Neurotrophic-Factor (BDNF) was assessed for neuronal brain development and synaptic plasticity (22), because BDNF has reported to be decreased in MS and correlated with severity of CNS injury (23). Interleukin-6 cytokine (IL-6), interleukin-8 chemokine (IL-8), interleukin-17 (IL-17), and tumor-necrosis-factor-alpha (TNF-α) were measured as inflammatory biomarkers (24). These cytokines have been shown to be elevated in MS and correlate with MS symptoms and CNS lesions (24, 25). Enzyme-Linked-Immunosorbent-Assays used for measurements and methodological details are provided in the Supplementary Material.

### Symptom Assessment

At the time of diagnosis, the subject could barely walk and had significant tingling sensations in extremities, 2-years prior to the FMT study. One-year prior to the FMT study, the subject was walking with gait issues requiring to use cane, after many rounds of physical therapy. Overall, the subject's primary MS symptom was abnormal gait. Subjective (questionnaire) and objective (gait metrics) methods were assessed for the impact of FMT on walking/gait status. The walking tasks included: (1) walking normally (“gait”); (2) walking while looking to the right or left (each gaze sustained throughout 6 m; “side gaze gait”); and (3) walking while alternating gaze right and left in a pedestrian manner (“alternating gaze gait”). Additional gait metrics included: stride time, stride distance, cadence, step width, average pelvis forward velocity, and pelvis smoothness. Full methodological details are provided in the Supplementary Material.

### Clinical and Dietary Questionnaires

To assess the impact of FMT on clinical symptoms, the subject completed the following validated questionnaires at all study visits: 12-item MS walking scale (MSWS-12) (26), and PROMIS gastrointestinal symptom scale (27). To determine whether the changes in microbiota community were affected by dietary habit, the subject completed these questionnaires: Automated Self-Administered 24-h (ASA24®) recall (28), and 24-h recall Food Timing Screener (FTS) and Food Timing Questionnaire (FTQ) (29). MRI of the brain and spine for lesions were examined week 0 (baseline) and week 39 (after FMT_1_ and FMT_2_) collection time points. Methodological questionnaire and MRI summary details are provided in Supplemental Material.

### Statistical Analysis

Significant differences for microbiome and pathway analysis were summarized over time points using the linear model R/Bioconductor software package *limma* (30), and adjusted with the stringent Bonferroni *post-hoc* test. Targeted SCFA metabolomics, serum biomarkers, and gait metrics significant changes over time were assessed by parametric repeated measures One-way ANOVA or non-parametric Friedman's test, based on Shapiro Wilk normality test of residuals and Levene homogeneity of variance, which provided a *P*-value for each test. *Post-hoc* test of Bonferroni provided an adjusted *P*-value, along with each collection visit's mean average comparison. Additionally, Pearson correlations (log10 transformed) associations and linear regression predictions were examined over time for all outcome experimental (e.g., gait, Supplementary Figure 2) and clinical variables. The *R, R*^2^ and *P*-values are reported. Graphs were created using GraphPad Prism (v8.2.1).

## Results

### Characterizations of Fecal Microbiome

**Figure 1:**
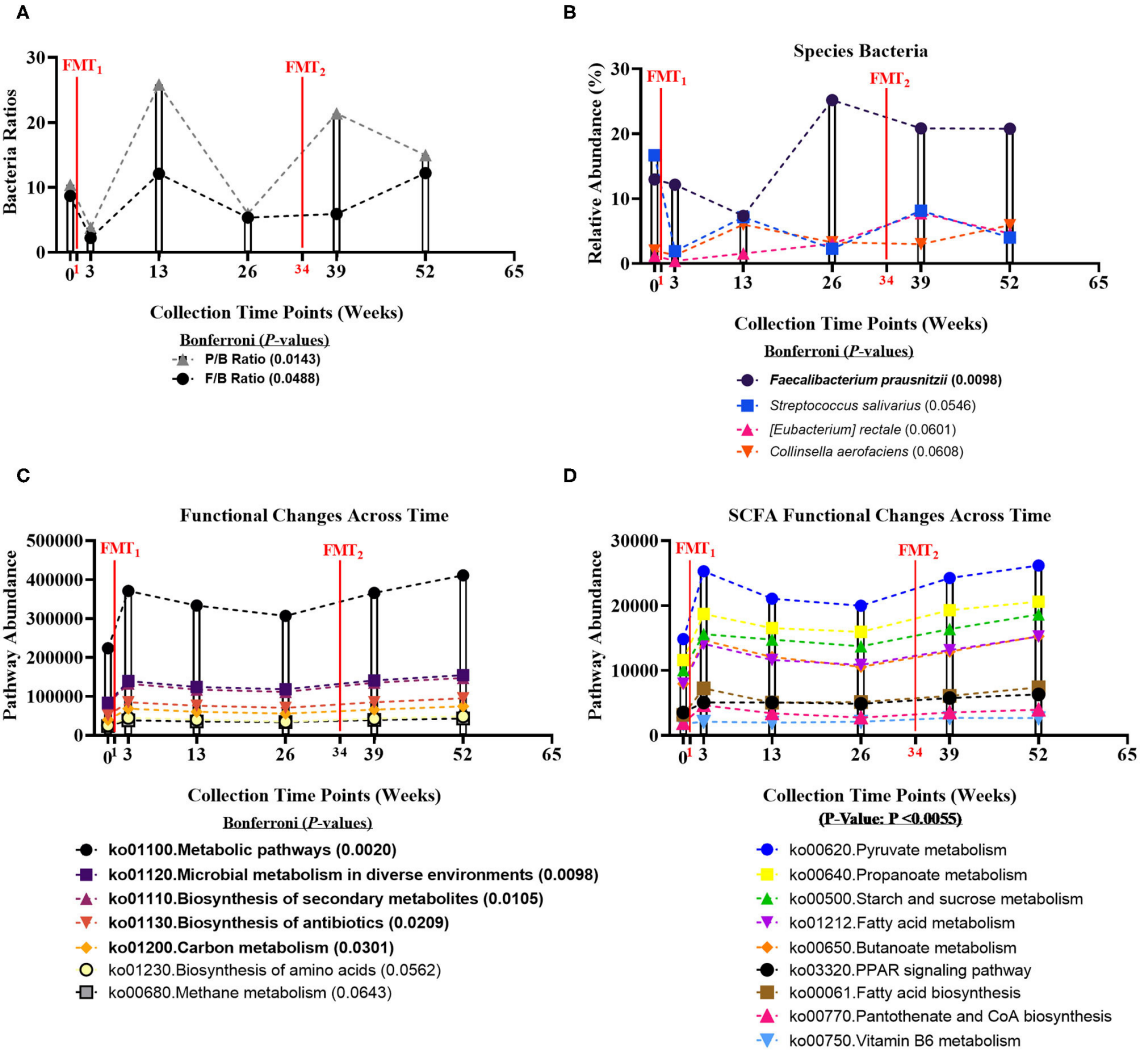
Bacteria ratios, species, and genomic pathways significant movements across time of study. Differential shifts in percent relative abundance across time for **(A)** bacterial ratios of Firmicutes-to-Bacteroidetes (F/B) (Bonferroni: *P* = 0.0488) and Prevotellaceae-to-Bacteroidaceae (P/B) (Bonferroni: *P* = 0.0143) and **(B)** butyrate-producing species, like *Faecalibacterium prausnitzii* (Bonferroni: *P* = 0.0098). Significant differential shifts in abundance across time for **(C)** non-targeted functional gene pathways (Bonferroni: *P* < 0.05); and **(D)** targeted SCFA functional gene pathways (*P*-value: *P* < 0.0055). Results were summarized over six collection time points using the linear model R/Bioconductor software package *limma*, and adjusted with the stringent Bonferroni *post-hoc* test. Directional mean trend dotted line shown across collection time points and FMT weeks.

Fecal microbiome was assessed for longitudinal shifts in microbial diversity, community structure for different high-level taxonomic groups (Bacteria, Archaea, Virus, Fungi), and functional gene content. Alpha diversity metrics, including Shannon index for Bacteria and Fungi, and Species Richness for Bacteria, significantly increased across time of study (Table 1). Microbial parameters, including ratios of Firmicutes-to-Bacteroidetes (F/B) (Bonferroni: *P* = 0.0488) and Prevotellaceae-to-Bacteroidaceae (P/B) (Bonferroni: *P* = 0.0143), significantly increased across time of study (Figure 1A). The relative abundance of only a single bacterial species, *Faecalibacterium prausnitzii* (butyrate-producing organism), significantly increased across time of study (Bonferroni: *P* = 0.0098) (Figure 1B). *Faecalibacterium prausnitzii* spiked higher before the second FMT and remained elevated, compared to baseline. The relative abundances of two other putative butyrate-producing species *Collinsella aerofaciens* (Bonferroni: *P* = 0.0601) and *Eubacterium rectale* (Bonferroni: *P* = 0.0608) increased across time of study, but corrected *P*-values were not significant at *P* = 0.05 level.

**Table 1:** Change in microbial diversity across time in an RRMS FMT subject^*^.

**Diversity metric (Taxon)**	**Average across time points**	**LFC**	**B Stats**	***P*-value**	**Bonferroni *P*-value**	***F***	***F*** ***P-value***
Shannon (Bacteria)	2.1433	0.0612	2.9939	**0.0072**	**0.0287**	8.9636	0.0028
Shannon (Archaea)	1.1283	0.0289	1.4144	0.1726	0.6905	2.0005	0.1572
Shannon (Fungi)	2.4650	0.0703	3.4409	**0.0026**	**0.0103**	11.8395	0.0006
Shannon (Virus)	1.4283	0.0411	2.0132	0.0577	0.2310	4.0532	0.0441
Simpson (Bacteria)	−0.0917	−0.0026	−2.5554	**0.0461**	0.1843	6.5300	0.0461
Simpson (Archaea)	−0.9350	−0.0299	−3.4112	**0.0160**	0.0641	11.6360	0.0160
Simpson (Fungi)	−0.0117	−0.0003	−1.1778	0.2867	1.0000	1.3871	0.2867
Simpson (Virus)	−0.5783	−0.0177	−3.1606	**0.0216**	0.0864	9.9895	0.0216
Richness (Bacteria)	12.1743	0.3461	3.3583	**0.0031**	**0.0125**	11.2784	0.0008
Richness (Archaea)	7.8332	0.2221	2.1552	**0.0435**	0.1740	4.6451	0.0311
Richness (Fungi)	8.9563	0.2541	2.4658	**0.0228**	0.0913	6.0804	0.0137
Richness (Virus)	8.8958	0.2554	2.4777	**0.0223**	0.0891	6.1388	0.0132
Evenness (Bacteria)	−0.9317	−0.0261	−2.7450	**0.0313**	0.1252	7.5352	0.0313
Evenness (Archaea)	−1.3083	−0.0402	−3.3618	**0.0137**	0.0549	11.3020	0.0137
Evenness (Fungi)	−0.1717	−0.0046	−1.4305	0.1994	0.7977	2.0463	0.1994
Evenness (Virus)	−1.1983	−0.0340	−2.8548	**0.0269**	0.1077	8.1500	0.0269

* *Alpha diversity calculated at the taxonomic level of species. LFC, log fold change. Linear model R/Bioconductor software package limma performed. Log2 fold change calculated for each time point. Weeks 0, 3, 13, 26, 39, 52 were examined across six time points. Bold value indicates adjusted P-values were significantly different at Bonferroni (P < 0.05); different at P < 0.05*.

Non-targeted analysis revealed that the abundances of five genomic pathways, including Metabolic pathways (ko01100: Bonferroni: *P* = 0.0020), Microbial metabolism in diverse environments (ko01120: Bonferroni: *P* = 0.0098), Biosynthesis of secondary metabolites (ko01110: Bonferroni: *P* = 0.0105), Biosynthesis of antibiotics (ko01130: Bonferroni: *P* = 0.0209), and Carbon metabolism (ko01200: Bonferroni: *P* = 0.0301), were enhanced after each FMT, and significantly increased across time of study (Figure 1C). Additionally, we hypothesized that FMT would increase pathways involved in SCFA-production. We observed SCFA genomic pathways were higher after each FMT, and increased across time of study (*P*-value: *P* < 0.0055) (Figure 1D). Complete microbiome and pathway analysis, including spouse observational comparison, depicted in Supplementary Figures 3–8, Supplementary Tables 2–8.

### Fecal Short-Chain Fatty Acids Metabolomics

Three principal colonic SCFAs (acetate, propionate, and butyrate) were examined across time. Subject's fecal SCFAs were within or above the normal SCFA concentration ranges (colon millimolar ratio of 60:20:20) (31). Following two FMTs, Propionate, Butyrate, Total SCFA, and Total Butyrate-to-Total SCFA ratio concentrations significantly increased at weeks 13 and 39. Comprehensive SCFA metabolomics results, including spouse observational comparison, depicted in Supplementary Figure 9, Supplementary Table 9.

### Longitudinal Assessment of Serum BDNF and Inflammatory Biomarkers

**Figure 2:**
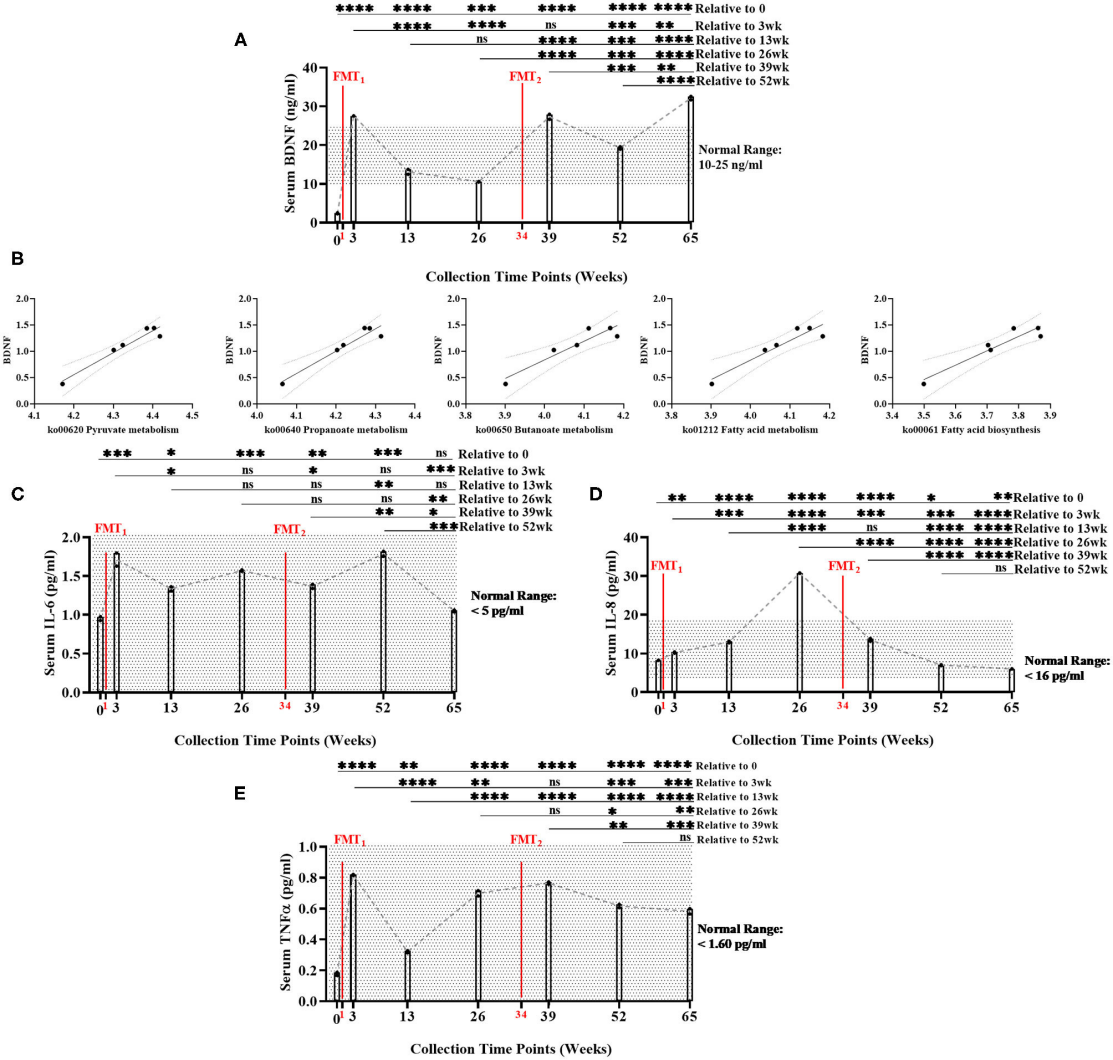
Measurement of serum biomarker changes over time of study. **(A)** Brain-derived neurotrophic factor (BDNF) (ng/ml) measurements significantly increased between baseline and end of study (Bonferroni: *P* < 0.0001). **(B)** Positive linear regression relationships between BDNF and SCFA functional gene pathways across time (*R*^2^ > 0.90, *P* < 0.008). **(C)** Interleukin-6 (IL-6) (pg/ml) did not significantly change between baseline and end of study (Bonferroni: *P* > 0.9999); **(D)** Interleukin-8 (IL-8) (pg/ml) significantly decreased between baseline and end of study (Bonferroni: *P* < 0.0012); and **(E)** Tumor necrosis factor alpha (TNF-α) (pg/ml) significantly increased between baseline and end of study, but remained with normal range (Bonferroni: *P* < 0.0001). Results were summarized over seven collection time points using repeated measures one-way ANOVA, and adjusted with the stringent Bonferroni *post-hoc* test. Biomarker's classified “normal ranges” derived from ELISA standard protocols. Directional mean trend dotted line shown across collection time points and FMT weeks. *Post-hoc* test adjusted *P*-values: ns, no significance, * < 0.05, ** < 0.01, *** < 0.001, **** < 0.0001.

Baseline serum BDNF (mean = 2.39 ng/ml) was below normal range of BDNF (10–25 ng/ml). Serum BDNF was significantly higher (Bonferroni: *P* ≤ 0.0002) at all time points after FMTs, especially Week 65 (mean = 32.12 ng/ml), compared to baseline (Figure 2A, Supplementary Table 10). After FMTs, serum BDNF significantly increased (Bonferroni: *P* < 0.0001) at weeks 3 and 39, compared to baseline and week 26.

Additionally, BDNF levels were significantly positively correlated with the abundance of microbial SCFA-pathway gene content (*R*^2^ < 0.90, *P* < 0.008) (Figure 2B, Supplementary Table 21). As the neuronal blood serum marker BDNF increased across time, our model predicted beneficial SCFA functional genomic pathways (i.e., pyruvate metabolism, propanoate metabolism, fatty acid biosynthesis, fatty acid metabolism, and butanoate metabolism) positively increased across time of study.

Overall, serum levels of inflammatory cytokines remained inhibited throughout the study: IL-6 (<5 pg/ml), IL-8 (<16 pg/ml), and TNF-α (<1.60 pg/ml) (Figures 2C–E, Supplementary Table 10). While remaining within normal range, IL-6, IL-8, and TNF-α did significantly increased following the first FMT. After the second FMT, IL-6, and TNF-α did not change, while IL-8 significantly decreased within normal range. *Post-hoc* results between baseline and end-of-study indicated that IL-6 levels did not change (Bonferroni: *P* > 0.9999); IL-8 decreased (Bonferroni: *P* = 0.0012); and TNF-α increased, but remained within normal range (Bonferroni: *P* < 0.0001). IL-17 (<0.190 pg/ml) was undetected in all seven collection time points (data not shown). Interestingly at 26 week, BDNF decreased and IL-8 increased (mean = 30.77 pg/ml—only time point above normal range). This data could suggest that the subject's overall health was regressing which could explain why the second FMT was administered.

### Longitudinal Assessment of Gait Metrics

**Figure 3:**
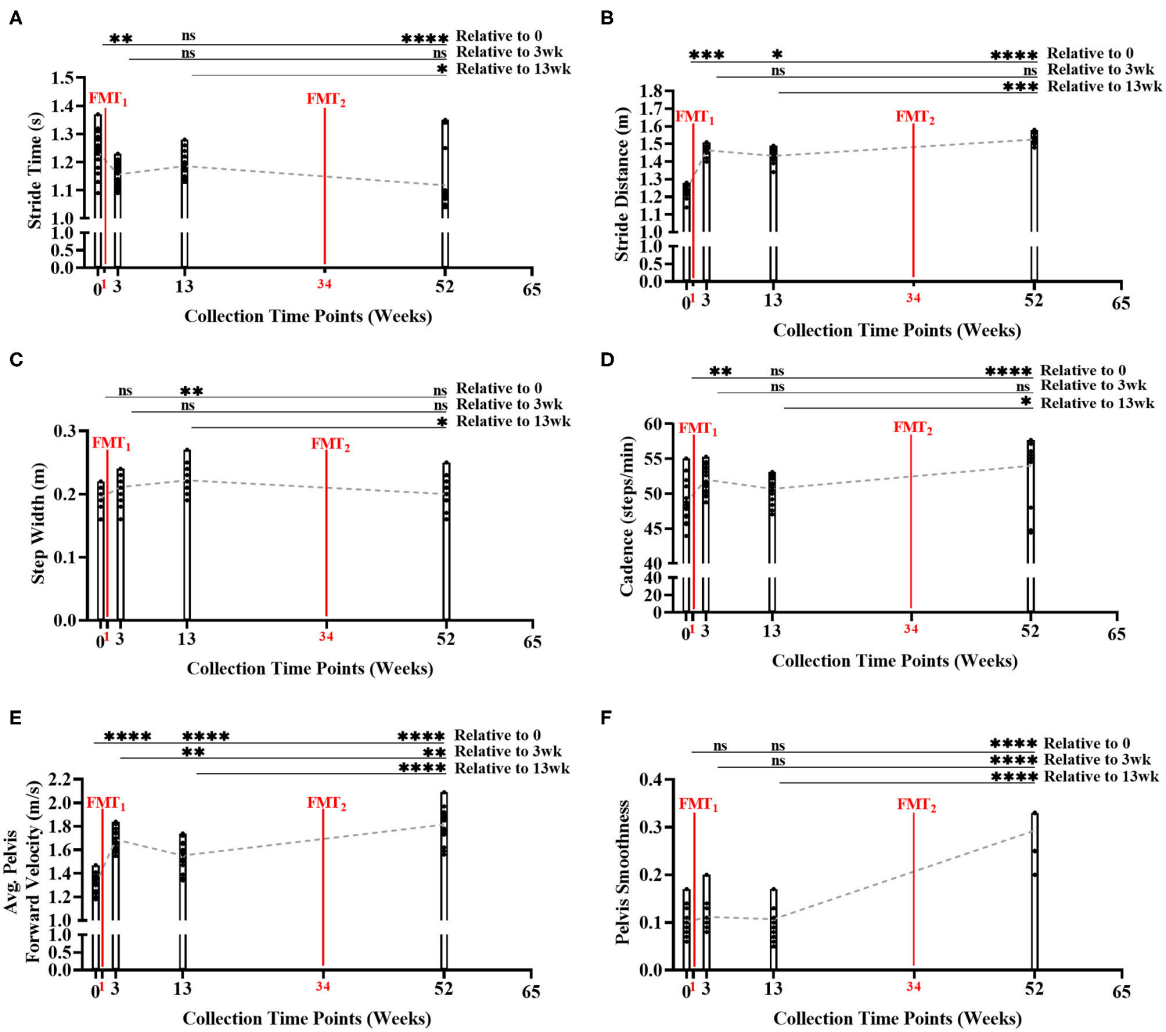
Measurement of normal gait metrics changes over time of study. Five of the six gait metrics significantly improved between baseline and end of study: **(A)** stride time (Bonferroni: *P* < 0.0001); **(B)** stride distance (Bonferroni: *P* < 0.0001); **(C)** step width (Bonferroni: *P* > 0.9999); **(D)** cadence (Bonferroni: *P* < 0.0001); **(E)** average forward velocity (Bonferroni: *P* < 0.0001); and **(F)** pelvis smoothness (Bonferroni: *P* < 0.0001). Results were summarized over four collection time points using parametric repeated measures 1-way ANOVA or non-parametric Friedman's test, and adjusted with the stringent Bonferroni or Dunn's multiple group comparison *post-hoc* tests. Directional mean trend dotted line shown across collection time points and FMT weeks. *Post-hoc* test's adjusted *P*-values: ns, no significance, * < 0.05, ** < 0.01, *** < 0.001, **** < 0.0001.

During the gait condition, the subject's normal gait metrics were primarily enhanced after each FMT, and significantly improved over time indicating improved walking and balance (Figures 3A–F, Supplementary Table 11). Stride time significantly decreased over time (Bonferroni: *P* < 0.0001) indicating subject's walking speed improved. Stride distance significantly increased over time (Bonferroni: *P* < 0.0001) suggesting subject's walking steps increased in distance. Step width did not change significantly from baseline to Week 52 (Bonferroni: *P* > 0.9999). Cadence significantly increased over time (Bonferroni: *P* < 0.0001) indicating subject's number of steps/minute increased. Average pelvis forward velocity significantly increased over time (Bonferroni: *P* < 0.0001), approximating the increased speed of the body center of mass. Pelvis smoothness significantly increased over time (Bonferroni: *P* < 0.0001), suggesting the subject's body walking motion was more fluid. Side gaze gait, alternating gaze gait, and within lab visit results in Supplementary Figures 10–12, Supplementary Tables 12–14.

Additionally, as subject's stride time decreased (i.e., walking speed improved) across time, our model again predicted beneficial SCFA functional genomic pathways (i.e., propanoate metabolism, starch and sucrose metabolism, vitamin B6 metabolism, fatty acid metabolism, and PPAR signaling pathway) significantly increased (*R*^2^ < 0.90, *P* < 0.05) across time; as well as, inflammatory blood marker IL-6 decreased across time (Supplementary Table 22).

### 12-Item MS Walking Scale Assessment

Twelve MS walking scale scores trended toward significant improvement in the subject across time. Four of the 12 MS walking scale scores were significantly associated to the predictor of time (weeks) (Supplementary Table 15). The subject's ability to climb up/or down stairs, walking balance, walking distance, and walking smoothness significantly improved across time.

As the subject's MS walking questionnaire scale scores decreased across time (i.e., improved walking scores), our model further supported predicted anti-inflammatory and immune system beneficial gut functional genomic pathways (i.e., vitamin B6 metabolism, flavone and flavonal biosynthesis, and PPAR signaling pathway) significantly increased (*R*^2^ < 0.90, *P* < 0.05) across time (Supplementary Table 23).

### PROMIS-GI Characteristics

Four GI symptoms scales were measures across time in the subject (Supplementary Table 16). At each collection time point, the PROMIS GI t-score for the RRMS subject was interpreted as “within normal limits” or “mild.” The RRMS subject's PROMIS GI t-score never was inferred to be “moderate” or “severe.” The mean ± standard deviation (SD) across all six time points collections for each PROMIS GI variable: Belly pain (56.20 ± 8.20 = mild); bowel incontinence (42 ± 7.70 = within normal limits); constipation (53.98 ± 1.88 = within normal limits); and gas & bloating (56.33 ± 1.58 = mild).

### Baseline and Longitudinal Assessment of Dietary Outcomes

Subject had been on gluten- and dairy-free high plant-based diet starting prior to FMTs, and maintained this diet throughout the study. Indeed, subject's fecal SCFA metabolite concentrations measured high at baseline. This is primarily due to predominantly plant-based diet that subject begun 2-years prior to the study. Subject's baseline records showed consumption of 12.5 servings of vegetables, 4.26 servings of fruit, 3.32 servings of legumes, and 3 servings of nuts/seeds per day. The subject's baseline (week 0) nutrient values and the total number of servings are depicted (Supplementary Table 17, Supplementary Figure 13).

Sixteen ASA24® nutrient variables were examined across time in the subject. Overall, the majority of ASA24® nutrient variables remained stable across time, with only two variables significantly changing (Supplementary Figure 14, Supplementary Table 18). The subject total fruit intake significantly decreased (*R*^2^ = 0.81, *P* = 0.014) across time, as the subject stopped consuming fruits at 39 and 52 weeks (Supplementary Figure 14H). The decrease in fruit intake is mysterious, as opposed to collection time point 0–26 weeks. It could be a result of the collection time point date where the subject by chance skipped or intentionally did not eat fruit. Oppositely, the subject consumed significantly more (*R*^2^ = 0.76, *P* = 0.024) whole grains at 39 and 52 weeks, whereas there was zero consumption between 0 and 26 weeks (Supplementary Figure 14L). The dietary food logs indicated that the whole grains consumed were both gluten free granola bars and bread at 39 and 52 weeks. Additionally, the subject's total number of servings and food group changes over time are shown (Supplementary Figures 15, 16). Finally, the ASA24® nutrient variables were normalized to energy intake (1,000 kcal), as is a common practice in nutrition literature with results shown (Supplementary Table 19, Supplementary Figure 17). Finally, the subject's ASA24® dietary variables were compared to experimental variables and significant relationships and predictor outcomes (Supplementary Table 24).

### Food Timing Screener/Food Timing Questionnaire Characteristic Outcomes

The FTS/FTQ data suggests that the FMTs modified the meal times, bedtime/wakeup, and hours slept of the subject, allowing for additional rest on the weekend (social jet lag). The subject indicated delayed wake-up times on the free days—Saturday and Sunday, allowing for additional hours (1.5 h) of extra sleep on the weekends. By waking up later on Saturday and Sunday, the RRMS's breakfast and lunch meal times where shifted later into those 2 days. Overall, the impact of extra sleep from the FMTs on free days, could suggest beneficial health effects on the body recovering from work days (Monday thru Friday) (Supplementary Table 20).

### Safety and Tolerability

No adverse effects were noted by the subject during the clinical study from the FMTs. It should be noted that the most common risks of FMT are transient cramping (1–3 days), bloating gaseousness, altered bowel habits (constipation more than diarrhea), and low-grade fever for no more than 12–24 h. Other risks from donor stool could include transmission of infectious organisms; allergic reaction to antigens; abdominal pain; and gut-related diseases, such as obesity/metabolic syndrome, autoimmune diseases, allergic/atopic disorders, and neurological disorders (32).

## Discussion

This is the first study to examine effects of FMT interventions on a RRMS subject's fecal microbiome using shotgun metagenomics, targeted metabolomics, and an array of clinical and physiological outcomes in a year-long study. Additionally, this study provides robust microbiome sequence data, including taxonomic and functional gene content, through the use of shotgun metagenome sequencing instead of traditional 16S ribosomal RNA gene amplicon sequencing (12).

We found FMT interventions were associated with improved microbiome, specifically an increase in relative abundance of “putative” anti-inflammatory butyrate-producing bacteria in the subject's stool, particularly *Faecalibacterium prausnitzii*. During the study, FMTs were associated with significant increased serum BDNF levels, subjective/objective evidence of improved gait/walking matrices (primary patient's MS complaint), and maintained normal GI symptoms, with no episode of RRMS symptom relapses/flare-ups over 12-months follow-up. During follow-up clinic visits in June and July of 2020, the subject reported no clinically significant relapses, and was able to walk with no help and no reported falls. Substantial increases in BDNF levels were observed at time points immediately after FMT relative to those before FMT.

In examining the subject's fecal microbiome, bacterial diversity increased with a limited number of microbial parameters and taxa features significantly affected during the study. Prior studies have linked an increased diversity to a healthier microbiome (33). Similarly, higher F/B ratios have been linked to increased SCFA-producing bacteria promoting gut health (34), and an elevated P/B ratio has been reported with consumption of high-fiber foods promoting gut health (35). The increase in bacterial diversity and parameters is partly due to increased shifts in the relative abundances of butyrate-producing bacterial species *Faecalibacterium prausnitzii, Eubacterium rectale*, and *Collinsella aerofaciens*. Furthermore, Butyrate and Total Butyrate-to-SCFA ratio concentrations significantly increased after FMTs. Butyrate-producing bacteria help regulate the immune system and intestinal epithelial barrier (17, 18, 36). Stimulating butyrate-production by microbiome could improve disease course, through correction of low levels of SCFA-producers in dysbiotic microbiomes, like MS (12, 37). It is noteworthy that at each collection time point, the subject's fecal microbiota composition was not similar to the household spouse control and stool SCFA levels were higher than the spouse. The lack of similarity between the subject and spouse fecal microbiota composition and SCFA levels suggests the changes found in the subject are primarily due to FMT, and are not simply the impact of environmental factors.

Our findings that FMT had a positive impact on MS clinical features are supported by prior reports (two patient case study) (38, 39). Like MS, FMT has been successfully used in other neurological disorders, supporting the notion that intestinal microbiota plays a central role in pathogenesis of chronic inflamamtory neurological diseases (21, 40, 41). Furthermore, there are an increasing number of ongoing human clinical trials evaluating FMT in RRMS that could shed more light on this potential therapy in MS (20).

Our study had several novel features: (1) longitutinal study that showed a sustained and durable impact of FMT on fecal microbiota community and clinical features of MS; (2) state of the art shotgun metagenomics to interrogate microbiome indicating FMT positively impacted both microbiota composition and function with increased levels of SCFA; (3) we found significant and sustained increase in serum BDNF levels, after FMTs. Serum BDNF has been shown to be decreased in MS patients (23), like our subject. BDNF is important for neuronal development and health acting as a “fertilizer” for brain cells, keeping them healthy, functioning and growing (22). One possible mechanism for increased BDNF is increased abundance of butyrate-producers after FMT, because BDNF-production is inhibited by immune systemic inflammatory state (42), and butyrate has anti-inflammatory activities (18). Furthermore, we found positive correlations between serum BDNF levels and microbiome SCFA-genomic pathways increasing over time of study. Finally, (4) the objective measures of gait showed that the subject's walking and balance metrics were enhanced after FMTs, and significantly improved over the course of the study. During the gait condition, five of the six gait metrics improved at 52 weeks compared to baseline, and these increased gait metrics corresponded to improved clinical patterns and subject's self-reported MS walking scale scores. These gait metric results alleviated one of the problematic MS symptoms of walking difficulty.

These results should be taken in context of the predominantly plant-based diet of the study subject, as previous reports found western, high-fat, low-fiber diet promoted dysbiosis and neuroinflammation in MS (43), while plant-based high-fiber diets decreased MS risks (44). However, prior to the study, while on the high-fiber diet, the subject's symptoms remained troublesome and led to the decision to test FMT. Nonetheless, high-fiber diet prior to and during the study could contribute to the subject's robust response to FMT and increased relative abundance of SCFA-producers (45).

This proof-of-concept study suggests that FMT might be an emerging treatment in RRMS. This study provides evidence of importance of intestinal microbiota in pathogenesis of MS and scientific rationale to help design future randomized controlled trials to determine whether FMT can modify the intestinal microbiome to alleviate MS pathology and symptoms. Randomized, placebo-controlled trials with appropriate sample size will be needed to determine the role of FMT for short- and long-term therapy for RRMS. The major limitation of this study is that it's a single-case, open-labeled design were we had no input or control over the FMT procedure administered by the Taymount Clinic. However, longitudinal monitoring of the subject is essential for providing insight into a potential mechanistic model of the microbiome-gut-brain-axis regulation of neuroinflammation in RRMS.

## Data Availability Statement

Accession number of the repository for shotgun metagenome sequencing data: Raw FASTQ data were deposited in the NCBI Sequence Read Archive under projects PRJNA529230.

## Ethics Statement

The studies involving human participants were reviewed and approved by Rush University Medical Center (RUMC) Institutional Review Board. The patients/participants provided their written informed consent to participate in this study.

## Author Contributions

AK designed and supervised the study. PE prepared the dataset, performed the statistical analysis, analyzed the results, and wrote the manuscript. AZ supervised and collected gait metrics. HR supervised and collected diet data. AN and SG supervised microbiome and bioinformatics analysis. LF supervised clinical questionnaires. CF supervised the study and laboratory experiments. SR performed the serum enzyme-linked immunosorbent assays and analysis. BH supervised the fecal targeted SCFA metabolomics. All authors have read and approved the final manuscripts.

## Conflict of Interest

The authors declare that the research was conducted in the absence of any commercial or financial relationships that could be construed as a potential conflict of interest.
