# Supplementary material for: Single-Arm, Non-randomized, Time Series, Single-Subject Study of Fecal Microbiota Transplantation in Multiple Sclerosis
Source: Front Neurol. 2020 Sep 8;11:978. doi: 10.3389/fneur.2020.00978 (PMC7506051; doi:10.3389/fneur.2020.00978)
Supplement: Supplementary file 1 [file Data_Sheet_1.pdf]

# *Supplementary Material*

## Methods and Materials

### Study Design

This was a prospective single-arm, non-randomized, time series, single-subject study. Single-subject studies are based on repeated observations within an individual over time and are acknowledged as an important research method for generating scientific evidence about the health or behavior of an individual. This design is desirable when the available patient pool is limited and thus, it is not optimal to randomize participants to a control arm. The subject serves as his/her own control, rather than using another individual/group. These designs are used primarily to evaluate the effect of a variety of interventions in early stage clinical research development.(1)

### Limitations

Beyond the major limitations mentioned in the brief report, there are several limitations to this study. The first is the use of FMT in the United States. There is mounting demand from clinicians and patients to use FMT for potential microbiota-derived diseases, including MS. However, the United States FDA ruled that FMT is both a drug and biologic, which is not currently approved for any clinical use, besides *Clostridium Difficile Infection* (CDI).

Another limitation is small sample size (single subject study). The FMT effects reported in this study may or may not have similar outcomes on different RRMS subjects. This study only examined a single subject for one year. Additional clinical trials of larger sample size will be required to examine the potential of FMT for the treatment of MS, and to determine the long-term effects and variability between subjects.

Furthermore, this study had no parallel longitudinal observational control arm and/or current standard of care for RRMS patients who otherwise satisfied the study's inclusion criteria based on their MS phenotype, demographics, disease duration and prior use of allowable MS therapies, was recruited as a comparison to measure the primary and secondary outcome measures. Thus, only one household spouse control fecal sample was collected for observational fecal comparisons. The spouse did not provide blood.

A limitation with diet was that it was assessed for only one day at each time point and may not accurately reflect the RRMS subject's usual diet. As in all gait studies, there is an inherent error that when subjects know their walking patterns are being recorded and studied, they may change their pattern. The gait tasks used only retroreflective markers instead of a full-body motion capture gait protocol. This focused the gait metrics on foot and pelvis-derived metrics, rather than those derived from the center of mass trajectory. Gait was assessed at four collection time points, and not six collection time points, due to scheduling issues. Also, this study primarily focused on GI primary and secondary outcome measurements. The Expanded Disability Status Scale (EDSS) was not collected. Finally, future studies should consider replicate fecal sampling (at least three stool samples) per time point, as well as more collection time points with a longer time series.

**Supplementary Table 1.** Demographic information of the RRMS subject and household spouse control.

| Characteristics | RRMS Subject (n = 1) | Household Spouse Control (n = 1) |
|---|---|---|
| Sex | Male | Female |
| Race | Caucasian | Caucasian |
| Age | 48 | 48 |
| Body Mass Index (BMI) | 27.1 | 20 |
| Age of onset of disease | 46 | None |
| Disease duration (years) | 2 | None |
| Relapsing-remitting MS (RRMS) – active or remission | Active RRMS: Presence of active lesions on brain or spinal cord MRI, in the past one year prior to baseline | None |
| Rate of Relapse | • Last significant relapse reported was one month prior to baseline collection time point (Week 0). When the relapse occurred, subject lost significant function in left hand (couldn't type or hold objects) and arm (strength).<br>• Subject did not experience any relapse during time of study, and not any physical relapses after study.<br>• During follow-up clinic visits in June and July of 2020, the subject reported no clinically significant relapses, and was able to walk with no help and no reported falls. | None |
| MS Therapy | Ocrelizumab (Ocrevus) 600mg IV (since 2017) | None |
| Medications other than MS Therapy | • Albuterol Inhaler<br>• Levothyroxine 150 mcg<br>• (Flomax) 0.4 mg PO Cp24 capsule<br>• Cholecalciferol<br>• Lisinopril (Prinivil, Zestril) 10mg PO tablet<br>• Prednisone (Deltasone) 10mg PO tablet<br>• Docusate Sodium (Colace) 100mg PO capsule | None |
| Gastrointestinal Disorder Exams | Normal | Normal |
| Probiotic Use (within four weeks of FMT) | None | None |
| Antibiotic Use (within four weeks of FMT) | None | None |
| Alcohol Use | None | None |
| Smoking Use | None | None |
| Aspirin Use | None | None |
| Chronic NSAID Use | None | None |
| Liver Disease (Cirrhosis or persistently abnormal AST or ALT 2X> normal) | None | None |
| Kidney Disease (creatinine > 2.0mg/dL) | None | None |
| Uncontrolled Psychiatric Illness | None | None |

| | | |
|---|---|---|
| Clinically Active Lung Disease or Decompensated Heart Failure | None | None |
| HIV Infection | None | None |
| Transplant Recipients (other than FMT) | None | None |
| Diabetes | None | None |
| Severe Malnutrition or Obesity with BMI > 40 | None | None |
| Pregnant or Lactating Women | None | None |
| Active Infections (untreated latent or active tuberculosis, HIV, hepatitis, syphilis or other major active infection) | None | None |
| Active Symptomatic *C. Difficile* Infection | None | None |
| Active Gastrointestinal Condition being Investigated (i.e. GI bleeding, colon cancer, active GI workup) | None | None |
| History of Known or Suspected Toxic Megacolon and/or known Small Bowel Ileus, Major Gastrointestinal Surgery (e.g. significant bowel resection) within three months before FMT (note that this does not include appendectomy or cholecystectomy) | None | None |
| History of Total Colectomy or Bariatric Surgery | None | None |
| Bristol Stool Score and *Description* | Baseline: Score of 5; *Soft blobs with clear cut edges*<br>3 weeks: Score of 4; *Like a smooth, soft sausage or snake*<br>13 weeks: Score of 4; *Like a smooth, soft sausage or snake*<br>26 weeks: Score of 3; *A sausage shape with cracks in the surface*<br>39 weeks Score of 4; *Like a smooth, soft sausage or snake*<br>52 weeks: Score of 4; *Like a smooth, soft sausage or snake* | Score of 4; *Like a smooth, soft sausage or snake* |

**Fecal Microbiota Transplantation Background**

An FMT is classified as a drug and biological product by the United States FDA. Currently, the United States FDA does not approve of a FMT for the treatment of MS. Based on the subject's personal decision, and not under the medical direction and/or guidance by the RUMC doctors, the RRMS subject underwent a FMT administered outside the United States, from a human microbiome biobank called the Taymount Clinic at Bahamas Medical Center (stool received from United Kingdom Certified Stool Bank), for the treatment of MS disease. The United States FDA defines a stool bank as an establishment that collects, prepares, and stores FMT products for distribution to other medical institutions, healthcare providers, or other entities for therapeutic or clinical research.(2) Stool biobanks are growing in number globally to help optimize the therapeutic use of stool samples for various human diseases, including inflammatory bowel disease (IBD), irritable bowel syndrome (IBS), obesity, neurodevelopmental disorders, autoimmune diseases and allergic diseases.(3)

**Outcome Measures**

The RRMS subject's biological samples were examined at the following time points: before stool transplant (baseline – week 0) followed by 3, 13, 26, 39, and 52 weeks. The primary outcome analysis was fecal non-targeted shotgun metagenome sequencing and taxonomic and functional gene profiling.(4) The longitudinal study design was used to determine the impact on the individual's microbiome diversity, microbial community structure, functional genomic pathways and sustainability of change. Secondary outcome measurements included analysis of fecal targeted SCFA metabolomics, serum biomarkers of systemic inflammation and brain-derived neurotrophic factor (BDNF), gait metric activity, and validated clinical questionnaires for MS walking scales scores, gastrointestinal symptoms, 24-hour recall diet and timing of eating and sleep.

Two FMT interventions occurred during the 52-week study: 1st FMT implant occurred between Baseline-week 0 and 3 weeks (Taymount Clinic Nassau Bahamas); 2nd FMT implant occurred between 26 and 39 weeks (at-home FMT booster). Additionally, the spouse of the RRMS subject, volunteered to donate one stool sample (baseline-week 0) to be used for the purposes of a secondary observational standard of control, for the fecal experiments. All procedures performed in the study involving human participants were in accordance with the ethical standards of the RUMC Institutional research committee and with the 1964 Helsinki declaration and its later amendments or comparable ethical standards.

We selected to do our post FMT evaluation at Week 3, following the first FMT, in order to give enough time for potential changes in microbiome to impact the systemic/neuro-inflammation and motor function motor changes, like gait. We performed subsequent examinations at Weeks 13 and 26 for short and mid-term assessment for the first FMT. Week 39 evaluated the post effects of the second FMT. Finally, we examined Week 52 for long term assessment for both the first and second FMT. These samples/data were collected to see if the FMT affected and/or alleviated RRMS symptoms, and whether any observed improvements were sustained in this proof-of-concept study. The study design is shown in (**Figure S1**).

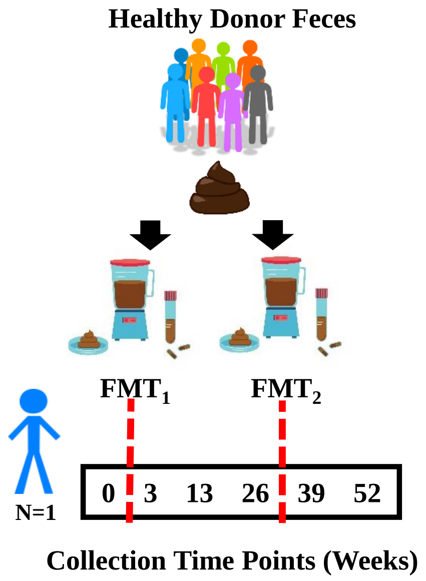


**Supplementary Figure 1.** Study design. Up to six collection time points: 0 (Baseline), 3, 13, 26, 39, and 52 weeks. Two fecal microbiota transplantations administered to RRMS subject ($FMT_1$: Taymount Clinic; $FMT_2$: Take-Home Booster).

## Fecal Collection

Fecal samples were self-collected at home, using an anaerobic home collection kit (BD Gaspak, Becton Dickinson and Company, Sparks, MD). Fecal samples were frozen at the time of collection until it was brought to the RUMC GI Laboratory. Fecal samples were stored at -80°C until analysis. Fecal samples were only subjected to a single freeze-thaw cycle. Fecal samples were interrogated using non-targeted shotgun metagenomics to characterize bacterial diversity, community structure, and functional gene pathways of the microbiome.(4) The fecal samples were also analyzed for targeted SCFA metabolites.

## Blood Collection

The RRMS subject's blood was collected via antecubital venipuncture into a BD (Becton, Dickinson and Company, NJ USA) vacutainer tube with clot activator and gel for serum separation, 10mL, red top (#367820) at each collection time point. Serum tube was inverted five times upon collection, to ensure mixing of clot activator with blood. In an upright position, at room temperature, incubation blood clotting time was for 30 minutes. Whole blood was then centrifuged at 4˚C, 3150 rpm, 15 minutes, with no brake, to separate blood from serum. Serum was aspirated from blood tube, taking care not to disturb the cell layer or transfer any cells, aliquoted into cryovials, and stored at -80°C until the time of analysis. Blood samples were only subjected to a single freeze-thaw cycle.

## Data Availability

Raw sequence data (FASTQ files) were deposited in the National Center for Biotechnology Information (NCBI) Sequence Read Archive (SRA), under the BioProject identifier PRJNA529230.

**Taxonomic Profiling**

Raw reads were mapped to the NCBI nucleotide (nt) database using Centrifuge.(5, 6) A least common ancestor algorithm was used to determine taxonomic annotations for each read. Taxonomic annotations were summarized across all reads to create counts per taxon. Raw counts were normalized to percentages for relative abundance. Four kingdoms were examined: bacteria, virus, archaea, and fungi. Each fecal sample was rarefied (bacteria: 1.2 million sequences/sample; fungi: 11,000 sequences/sample; virus: 3,500 sequences/sample; archaea: 3,500 sequences/sample).

**Functional Profiling**

Raw reads were aligned to the SwissProt protein database using DIAMOND.(7, 8) Functional gene annotations for each read were obtained using a consensus algorithm and then summarized across all reads to create counts per orthologous ID. Higher level summaries of orthologous functions are created using KEGG module, pathway, and BRITE hierarchical annotations.(9) Raw counts were normalized to percentages for relative abundance.

**Targeted Short-Chain-Fatty-Acids Metabolomics**

Feces were dissolved in purified water (1:5, w/v) and homogenized by vortexing and shaking by hand. The samples were centrifuged at 10,000 g at 4 °C for 10 min. Supernatants was filtered through a 0.22 μm membrane for SCFA content analysis. The SCFA analysis was conducted by gas chromatography (GC), as described by Kaur et al.(10) One hundred microliter of a mixture containing 50 mM 4-methyl-valeric acid, 5% meta-phosphoric acid, and 1.56 mg/mL of copper sulfate was added to 400 μL of filtered fecal solution. Then 1 μL of sample was injected to an Agilent 7890A GC (Agilent) equipped with a fused silica capillary column (Nukol, Supelco nr 40369-03A, 30 m × 0.25 mm, id 0.25 μm, Palo Alto, CA, USA). Helium was used as carrier gas at a flow rate of 1.2 mL/min. The GC conditions used were as follows: injection volume (1 μL), split ratio (1:100), the oven temperature was set at 185 °C. All measurements were performed in triplicates per time point.

**Inflammation and Neuronal Activation Measurements**

The RRMS subject provided blood serum samples for six collection time points, plus an extra seventh time point: baseline-week 0, 3, 13, 26, 39, 52, and 65 weeks. Enzyme-Linked Immunosorbent Assays (ELISA) were employed using only the RRMS subject's collected time point blood serum samples: interleukin-6 (IL-6) [cytokine marker that measures acute phase reaction, inflammation, hematopoiesis, bone metabolism, and cancer progression]; interleukin-8 (IL-8) [chemokine marker that measures inflammation where it promotes neutrophil infiltration and activation]; interleukin-17 (IL-17) [crucial effector cytokine with potent proinflammatory effects that induces the expression of other proinflammatory cytokines like tumor necrosis factor-α and chemokines, attracts neutrophilic leukocytes, and enhances the maturation of dendritic cells]; tumor necrosis factor alpha (TNF-α) [marker that plays a central role in inflammation, apoptosis, and immune system development]; and the Brain-Derived Neurotrophic Factor (BDNF) [marker protein that regulates many aspects of neuronal development and function in the nervous system]. The ELISA kits used were human IL-6 (HS600B; R&D Systems), human IL-8 (HS800; R&D Systems), human IL-17 (HS170; R&D Systems), human TNF-alpha (HSTA00E; R&D Systems), and human

free BDNF (DBD00; Hycult Biotech). All kits were used according to the manufacture's protocol. All measurements were performed in duplicates per time point.

## Gait Metric Activity

The RRMS subject performed walking tasks at a self-selected speed at each of four time point collections: baseline-week 0, 3, 13, and 52 weeks. The walkway was approximately 6 m, which allowed approximately two or three steady-state strides each time the RRMS subject walked through the lab. The walking tasks included: (1) walking normally ("gait") (2) walking while looking to the right or left (each gaze sustained throughout 6 m; "side gaze gait"), and (3) walking while alternating gaze right and left in a pedestrian manner ("alternating gaze gait"). During walking, retroreflective markers were placed bilaterally on the RRMS subject's pelvis (anterior superior iliac spine and posterior superior iliac spine) and feet (calcaneus, 5th metatarsophalangeal joint, and 1st metatarsophalangeal joint). These markers were selected to assess balance by measuring traditional foot-derived gait metrics and the smoothness of the RRMS subject's pelvis motion (as an approximation of his center of mass motion). Optical motion capture cameras measured the position of these markers in 3D space (100fps; Optitrack, Corvallis, OR, USA). A total of 18 data points per time point were measured for "gait". A total of six data points per time point were measured for both "side gaze gait" and "alternating gaze gait".

## Gait Metric Analysis

Traditional foot-derived gait parameters and pelvis smoothness were calculated using custom code in MATLAB (Mathworks, Novi, MI, USA) during steady-state strides. The data analyses were blinded from the researcher such that the lab visit number was masked during calculations and statistical analyses (researcher only had access to pelvis and foot marker 3D locations).

The following gait metrics were analyzed during the middle two strides (between consecutive left foot heel strikes) of the approximately 6 m walkway: stride time, stride distance, cadence, step width, average pelvis forward speed, and pelvis smoothness. Stride time (seconds) is the duration between right heel strikes.(11, 12) Stride distance (meters) is the length of a straight line between the positions of the left heel at each heel strike.(11, 13, 14) Cadence is the number of steps per minute and included for clinical comparison, though it is calculated from stride time.(15, 16) Step width (meters) is the mediolateral (side-to-side) distance between the first left heel strike and next right heel strike. Clinically, a wide step width is indicative of widening the base of support to facilitate balance.(17, 18) Average pelvis forward speed (meters per second) was calculated by measuring the distance travelled by the pelvis center of mass in the anterior (forward) direction during each stride divided by stride time. Pelvis smoothness was defined as the reciprocal of the number of peaks (or fluctuations) in the pelvis horizontal speed.(19, 20) Therefore, if there were a greater number of fluctuations in the pelvis horizontal speed, the pelvis smoothness metric was lower than if there were few fluctuations in horizontal speed.(21, 22) (Though this method of calculating pelvis smoothness is simple and discretized, it is not influenced by the magnitude of the speed as are other smoothness metrics).

## Clinical Questionnaires

### 12-Item Multiple Sclerosis Walking Scale.

The RRMS subject completed the 12-item MS walking scale for all six collection time points. The 12-item MS walking scale (MSWS-12) is a validated, patient self-reported outcome measure of the impact of MS on the individual's walking ability.(23) Higher scores indicate a greater impact from MS on walking than lower scores. Scale range from 1 (no impact) to 5 (high impact). A total of twelve questions were asked. Individual item scores are summed to achieve a total score that ranges from 12 to 60. This total score is then transformed to achieve a range of 0–100, with higher scores reflecting a greater impact of MS on walking.

### Patient-Reported Outcomes Measurements Information System Gastrointestinal Symptoms Scale.

The RRMS subject completed the validated NIH Patient-Reported Outcomes Measurements Information System (PROMIS) gastrointestinal symptom scale for all six collection time points. The NIH PROMIS uses eight GI symptom scales that are used for clinical care and research across the full range of GI disorders.(24) Only four GI symptoms scales were measured across time for this study. Belly pain (six questions), bowel incontinence (four questions), constipation (9 questions), and gas & bloating (12 questions) were answered by the RRMS subject. Higher score denoted more GI symptoms. Lower scores denoted less GI symptoms. Scores range from 20 (low) to 80 (high). A score of 50 denoted as the general population.

## Dietary and Sleep Assessments

### Automated Self-Administered 24-Hour Recall.

The RRMS subject completed the Automated Self-Administered 24-hour (ASA24®) recall for all six collection time points. The ASA24® recall is a valid and reliable web-based tool developed by the National Cancer Institute to capture 24-hour dietary intake.(25-27) The RRMS subject reported each food item that they consumed in the last 24 hours using the gold standard, automated multiple-pass method (AMPM).(25) The Computer-Assisted Self-Interviewing (CASI) methodology is used to guide the respondent through multiple steps of recalls that include reporting each meal or snack or any other time that food or beverage were consumed, a comprehensive list of foods and drinks consumed, and finally a detail step that includes quantity of food consumed, any forgotten foods, and a final review. The criterion validity of the ASA24® recalls is supported by high agreement (~80%) with traditional interviewer-administered recalls and comparable energy intake estimates between ASA24® recalls and AMPM in healthy men and women.(28, 29) The ASA24® recall was used to identify dietary intake consistency to determine its influence on gut microbiota and secondary outcome measurements.

### Food Timing Screener/Food Timing Questionnaire.

The RRMS subject completed the 24-hour recall Food Timing Screener (FTS) and Food Timing Questionnaire (FTQ) for all six collection time points. The FTQ and FTS measure food and timing of eating and sleeping habits on two types of days: the days an individual goes to work (normally a week day), and the days an individual has more free time (normally a weekend).(30) FTS is a structured food demographics questionnaire developed to assess food and sleep timing. The questionnaire consists of questions asking the RRMS subject's eating and sleeping habits on work days and non-work days. Questions include the time of the main meal during work and non-work days, time of last meal before bed, consistency of dinner within work and non-work days, and consistency of breakfast, lunch, and dinner between work and non-work days. FTQ consisted of questions of which days did the RRMS subject worked or rested, as well as what time did the RRMS subject usually eat meals or snacks each day. The RRMS subject's hours awake, hours asleep and social jet lag were determined.

## Statistical Analysis

### Alpha, Beta and Functional Diversities.

Analyses of alpha diversity (α-diversity) indices (within-sample), beta diversity (β-diversity) (between-sample), and biological functional genomic pathways were examined within feces across the six collection time points: baseline-week 0, 3, 13, 26, 39, and 52 weeks. Spouse fecal sample (baseline-week 0) was used as an observational standard of control. Alpha diversity indices (i.e., Shannon, Simpson, richness, and evenness) were generated from rarefied sequences using the package 'vegan' implemented in the R programming environment.(31) Alpha-diversity indices were calculated such as: Shannon index (H'=-∑ sum(Pi/log(Pi)) = the relative abundance and evenness of each species present), Simpson's index (D=∑(Pi2)) = characterizes species diversity in a community), richness (total number of different species in a sample/community), and Pielou's evenness (J'=H'/log(S)) = how evenly the samples in a community are distributed among the different species).

Alpha and β-diversities were examined at the taxonomic levels of phylum, genus and species. Significant differences for α-diversity indices (i.e., bacteria, archaea, fungi, virus), β-diversity relative abundance of individual taxa (i.e., bacteria, archaea, fungi, virus), and the relative abundance of functional genomic pathway results were summarized over six collection time points using the linear model R/Bioconductor software package *limma*,(32) and adjusted with the stringent Bonferroni post-hoc test. The *limma* model uses the simple Bayesian model by borrowing information from the ensemble of variables to aid with inference about each individual variable. This creates a model that is able to detect different time trends across the study's six collection time points, using time as a continuous independent variable. This analysis is ideal for experiments with a small number of samples, does not require multiple replicates, nor is it necessary for two groups to be observed at identical time points. The *limma* software has been used successfully in thousands of published biological studies using data from a variety of genomic technologies.

Additionally, Pearson correlations (log10 transformed) associations and linear regression predictions between individual taxa (i.e., bacteria, archaea, fungi, virus), functional

genomic pathways and either experimental or clinical variables were examined over time. The R, $R^2$ and P-values are reported.

**Short-Chain-Fatty-Acids Metabolomics Data.**

Fecal targeted SCFA metabolite concentration changes over the six collection time points for acetate (mM/kg), propionate (mM/kg), butyrate (mM/kg), total SCFA (mM/kg), and total Butyrate-to-total SCFA ratio (mM/kg) were examined in the RRMS subject. Parametric repeated measures One-way ANOVA, based on Shapiro Wilk normality test of residuals and Levene homogeneity of variance, provided a P-value for each test. Post-hoc test of Bonferroni provided an adjusted P-value, along with each collection visit's mean average comparison. Each collection point had three measurements. Spouse fecal sample (baseline-week 0) used as an observational standard of control. Additionally, Pearson correlations (log10 transformed) associations and linear regression predictions between SCFA metabolite concentrations and either experimental or clinical variables were examined over time. The R, $R^2$ and P-values are reported.

**Blood Serum Biomarker Data.**

Measurement of blood serum biomarker changes over seven collection time points for BDNF (ng/ml), IL-6 (pg/ml), IL-8 (pg/ml), and TNF-α (pg/ml), were examined in the RRMS subject. Parametric repeated measures One-way ANOVA, based on Shapiro Wilk normality test of residuals and Levene homogeneity of variance, provided a P-value for each test. Post-hoc test of Bonferroni provided an adjusted P-value, along with each collection visit's mean average comparison. Each collection point had two measurements. Additionally, Pearson correlations (log10 transformed) associations and linear regression predictions between serum blood biomarkers and either experimental or clinical variables were examined over time. The R, $R^2$ and P-values are reported.

**Gait Metrics Data.**

The primary goal was to compare normal walking across the four collection time points: baseline-week 0, 3, 13, and 52 weeks. During this analysis, 18 steady-state "gait" strides per collection time point were compared across the four lab visits. Similarly, the comparison of each "side gaze gait" and "alternating gaze gait" tasks across the four lab visits, but only included six steady-state strides per collection time point. During a secondary set of comparisons across the three walking tasks performed within each lab visit (i.e., gait vs. side gaze gait vs. alternating gaze gait), metrics were compared using six steady-state strides (i.e., six dataset points per group).

To determine if the six gait metrics significantly changed over the course of time, parametric repeated measures One-way ANOVA or non-parametric Friedman's test, based on Shapiro Wilk normality test of residuals and Levene homogeneity of variance, provided a P-value for each test. Post-hoc test of Bonferroni (parametric) or Dunn's multiple comparison test (non-parametric) provided an adjusted P-value, along with each visit time's mean average comparison.

Statistical results during each week's collection across gait metrics comparisons used an omnibus Kruskal-Wallis test to determine chi-square value and P-value. When this test was significant at $\alpha=0.05$ level, post-hoc Mean Rank tests were performed to determine P-value and confidence interval. The non-parametric Kruskal-Wallis test assumed that each group compared was independent.

Pearson correlation was used to determine which gait metrics were highly correlated to each other across time (baseline-week 0, 3, 13, 52 weeks), in order to reduce the large number of gait variables. Three gait metrics were found to encompass 100% of all 18 gait metric measurements: stride time, pelvis smoothness & step width, with collection time points (0, 3, 13, and 52 weeks) visualized using a Principle Coordinates Analysis (PCA) plot (**Figure S2**). These three gait metrics were than used for further Pearson correlations (log10 transformed) associations and linear regression predictions between either experimental or clinical variables examined over time. The R, $R^2$ and P-values are reported.

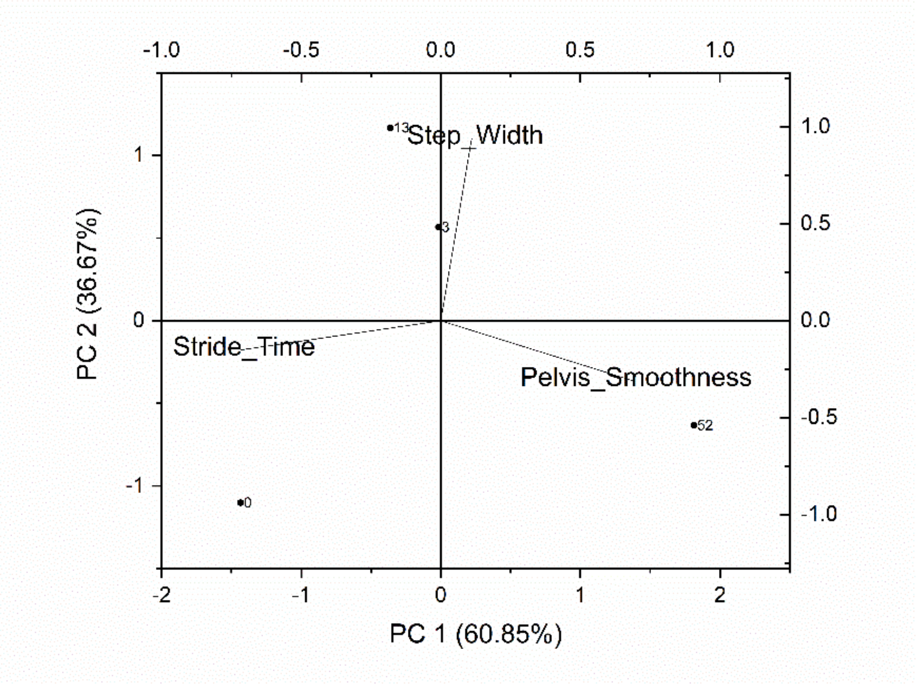


**Supplemental Figure 2.** Principle Coordinates Analysis (PCA) of gait metrics. The PCA indicates the three gait metrics that encompass all 18 gait metrics measurements: stride time, pelvis smoothness & step width, with collection time points (0, 3, 13, and 52 weeks).

**Multiple Sclerosis Walking Scale Scores.**

Linear regression relationships between MS Walking Scale Questionnaire variables were examined across six collection time points, in the RRMS subject. Dependent variable was walking score metric. Independent variable was time (weeks). The slope, $R^2$ and P-values are reported. Additionally, Pearson correlations (log10 transformed) associations and linear regression predictions between MS walking scale scores and either experimental or clinical variables were examined over time. The R, $R^2$ and P-values are reported.

**PROMIS-GI Data.**

The average scores for the general population and the RRMS subject were examined across six collection time points. A score of 50 = mean of general population reference sample. By design, all PROMIS scales are normalized to a score of 50 and standard deviation of 10 in the general population using a T-metric. Higher score denotes more GI symptoms. Lower score denotes less GI symptoms. Additionally, Pearson correlations (log10 transformed) associations and linear regression predictions between PROMIS scores and either experimental or clinical variables were examined over time. The R, $R^2$ and P-values are reported.

**ASA24® Nutrients and Baseline Data.**

Linear regression relationships between ASA24® nutrients were examined across six collection time points, in the RRMS subject. Dependent variable was ASA24® nutrient variables. Independent variable was time (weeks). The slope, $R^2$ and P-values are reported. Additionally, the ASA24® nutrient data was normalized to energy (kcalories) intake. The linear regression relationships between ASA24® nutrition (per 1000 kcal) variables across six visits are also shown. Furthermore, baseline-week 0 total number of servings data is reported. Finally, Pearson correlations (log10 transformed) associations and linear regression predictions between ASA24® nutrients and either experimental or clinical variables were examined over time. The R, $R^2$ and P-values are reported.

As serving size differed based on individual foods within category, servings were determined by looking at foods eaten that day of the first recall (baseline). Radial graphs were used to show baseline number of servings, as well as the number of servings over time. A bar graph indicated the food group changes over time.

**FTQ/FTS Data.**

Significant rhythm trend correlation and regression relationships between food timing screener/food timing questionnaire data across six collection time points, were examined in the RRMS subject. The R, $R^2$, P-value, Cohen’s d, and effect size (calculated using mean and standard deviation between variables) are reported. Cohen’s d and effect size (ES-r) measures the magnitude of a treatment effect: 0.2 = small effect; 0.5 = moderate effect; 0.8 or > = large effect.

## RESULTS

### Longitudinal Analysis of Fecal Shotgun Metagenomics

To assess overall differences in fecal microbial diversity, community structure, and functional profiling in the RRMS subject across time, calculated measures of α-diversity and metagenomes of taxa and functional genomic pathways were examined. Please note that only stringent corrected Bonferroni P-values (*P*<0.05) are reported. For additional statistical results, the data tables also depict less stringent *P*<0.05 different trends across time points.

At the taxonomic level of phylum, the RRMS subject had significant movements (Bonferroni: P<0.05) in the relative abundances across time for bacteria phyla: Firmicutes, Actinobacteria, Proteobacteria, and Bacteroidetes; archaea phylum: Euryarchaeota; fungi phyla: Ascomycota and Basidiomycota; hierarchy of virus: Unidentified Phage, Caudovirales, and Herpesvirales (**Table S2, Figure S3A-D**). Additionally, these phyla percent relative abundances (>1%) at each fecal collection time point, along with the observational standard spouse control, are shown for bacteria, virus, fungi and archaea (**Figure S4A-D**). The ratios of Firmicutes-to-Bacteroidetes (F/B) and Prevotellaceae-to-Bacteroidaceae (P/B) both had a significant increased shift from baseline and across time (**Table S2)**.

**Supplementary Table 2.** At the taxonomic level of phylum, *limma* powers differential relative abundance analyses across time for bacteria, archaea, fungi and virus in the RRMS FMT subject.

| Phylum or Hierarchy | Average across time points | LFC | B-Stats | *P*-value | Bonferroni *P*-value | F | F *P*-value |
|---|---|---|---|---|---|---|---|
| Phylum Bacteria | | | | | | | |
| Firmicutes | 19.4682 | 0.5551 | 3.6103 | **0.0008** | **0.0069** | 13.0339 | 0.0003 |
| Proteobacteria | 17.2637 | 0.4859 | 3.1599 | **0.0028** | **0.0254** | 9.9852 | 0.0016 |
| Actinobacteria | 16.7868 | 0.4863 | 3.1627 | **0.0028** | **0.0252** | 10.0025 | 0.0016 |
| Bacteroidetes | 16.7257 | 0.4696 | 3.0542 | **0.0038** | **0.0340** | 9.3283 | 0.0023 |
| Cyanobacteria | 11.3601 | 0.3218 | 2.0932 | **0.0420** | 0.3780 | 4.3816 | 0.0363 |
| Spirochaetes | 10.5796 | 0.3003 | 1.9531 | 0.0570 | 0.5134 | 3.8146 | 0.0508 |
| Deinococcus-Thermus | 10.1938 | 0.2919 | 1.8986 | 0.0640 | 0.5764 | 3.6045 | 0.0576 |
| Verrucomicrobia | 11.0301 | 0.2906 | 1.8898 | 0.0652 | 0.5871 | 3.5714 | 0.0588 |
| Tenericutes | 10.1400 | 0.2880 | 1.8732 | 0.0675 | 0.6078 | 3.5091 | 0.0610 |
| Bacteria Ratios | | | | | | | |
| Firmicutes-to-Bacteroidetes (phylum level) | 2.7425 | 0.0854 | 2.6468 | **0.0244** | **0.0488** | 7.0058 | 0.0081 |
| Prevotellaceae-to-Bacteroidaceae (family level) | 3.4804 | 0.1086 | 3.363 | **0.0071** | **0.0143** | 11.314 | 0.0007 |
| Phylum Archaea | | | | | | | |
| Euryarchaeota | 11.7081 | 0.3329 | 3.5212 | **0.0031** | **0.0093** | 12.3986 | 0.0008 |
| Crenarchaeota | 6.2456 | 0.1747 | 1.9386 | 0.0716 | 0.2148 | 3.7581 | 0.0567 |
| Thaumarchaeota | 5.8198 | 0.1606 | 1.7858 | 0.0944 | 0.2831 | 3.1890 | 0.0786 |
| Phylum Fungi | | | | | | | |
| Ascomycota | 13.0209 | 0.3703 | 3.7409 | **0.0008** | **0.0046** | 13.9946 | 0.0007 |
| Basidiomycota | 11.1940 | 0.3170 | 3.3296 | **0.0023** | **0.0139** | 11.0864 | 0.0021 |
| Mucoromycota | 7.4430 | 0.2102 | 2.3689 | **0.0245** | 0.1469 | 5.6115 | 0.0237 |
| Microsporidia | 6.0201 | 0.1711 | 1.9716 | 0.0579 | 0.3476 | 3.8871 | 0.0569 |
| Chytridiomycota | 5.6617 | 0.1624 | 1.8831 | 0.0694 | 0.4165 | 3.5462 | 0.0683 |
| Fungi;Other | 5.4613 | 0.1561 | 1.8135 | 0.0798 | 0.4787 | 3.2886 | 0.0786 |
| Hierarchy of Virus | | | | | | | |
| s_Unidentified Phage | 10.9101 | 0.3122 | 4.2402 | **0.0006** | **0.0211** | 17.9793 | 0.0006 |
| o_Caudovirales | 9.3943 | 0.2624 | 3.8848 | **0.0013** | **0.0445** | 15.0916 | 0.0013 |

| | | | | | | | |
|---|---|---|---|---|---|---|---|
| o_Herpesvirales | 7.5042 | 0.2171 | 3.8695 | **0.0014** | **0.0460** | 14.9731 | 0.0014 |
| f_Mimiviridae | 5.9101 | 0.1781 | 3.7154 | **0.0019** | 0.0636 | 13.8040 | 0.0019 |
| f_Retroviridae | 5.8083 | 0.1713 | 3.5612 | **0.0026** | 0.0882 | 12.6824 | 0.0026 |
| f_Poxviridae | 4.8668 | 0.1432 | 3.2093 | **0.0055** | 0.1854 | 10.2994 | 0.0055 |
| f_Microviridae | 3.8642 | 0.1293 | 3.0399 | **0.0078** | 0.2646 | 9.2408 | 0.0078 |
| s_Streptococcus phage 20617 | 3.4632 | 0.1179 | 3.0227 | **0.0081** | 0.2743 | 9.1364 | 0.0081 |
| g_Pandoravirus | 3.7013 | 0.1176 | 2.9529 | **0.0093** | 0.3172 | 8.7196 | 0.0093 |
| f_Flaviviridae | 4.0524 | 0.1181 | 2.7989 | **0.0128** | 0.4366 | 7.8340 | 0.0128 |
| f_Reoviridae | 3.5300 | 0.1100 | 2.7878 | **0.0131** | 0.4467 | 7.7720 | 0.0131 |
| f_Phycodnaviridae | 5.0305 | 0.1336 | 2.7693 | **0.0137** | 0.4641 | 7.6691 | 0.0137 |
| o_Nidovirales | 3.3356 | 0.1068 | 2.7602 | **0.0139** | 0.4729 | 7.6187 | 0.0139 |
| o_Picornavirales | 4.2960 | 0.1201 | 2.7494 | **0.0142** | 0.4835 | 7.5593 | 0.0142 |
| f_Baculoviridae | 4.0397 | 0.1159 | 2.7370 | **0.0146** | 0.4960 | 7.4913 | 0.0146 |
| o_Bunyavirales | 3.4578 | 0.1068 | 2.6664 | **0.0169** | 0.5733 | 7.1096 | 0.0169 |
| o_Mononegavirales | 3.3053 | 0.1025 | 2.6187 | **0.0186** | 0.6319 | 6.8577 | 0.0186 |
| f_Orthomyxoviridae | 3.3077 | 0.1022 | 2.6044 | **0.0191** | 0.6506 | 6.7827 | 0.0191 |
| s_Bifidobacterium phage Bbif-1 | 3.1548 | 0.1004 | 2.5827 | **0.0200** | 0.6800 | 6.6701 | 0.0200 |
| f_Potyviridae | 3.3616 | 0.1005 | 2.5320 | **0.0222** | 0.7534 | 6.4109 | 0.0222 |
| f_Iridoviridae | 3.5309 | 0.1003 | 2.4550 | **0.0259** | 0.8797 | 6.0269 | 0.0259 |
| f_Adenoviridae | 2.9389 | 0.0910 | 2.3865 | **0.0297** | 1.0000 | 5.6953 | 0.0297 |
| f_Polydnaviridae | 3.0369 | 0.0838 | 2.1127 | 0.0506 | 1.0000 | 4.4636 | 0.0506 |
| o_Tymovirales | 2.4281 | 0.0763 | 2.0593 | 0.0561 | 1.0000 | 4.2406 | 0.0561 |
| LFC = log fold change. *limma* software R program. Log2 fold change calculated for each time point. Weeks 0, 3, 13, 26, 39, 52 were examined across six time points. Taxa abundance filtered at (>0.1%) for analysis. Hierarch taxonomic levels: o = order, f = family s = species | | | | | | | |

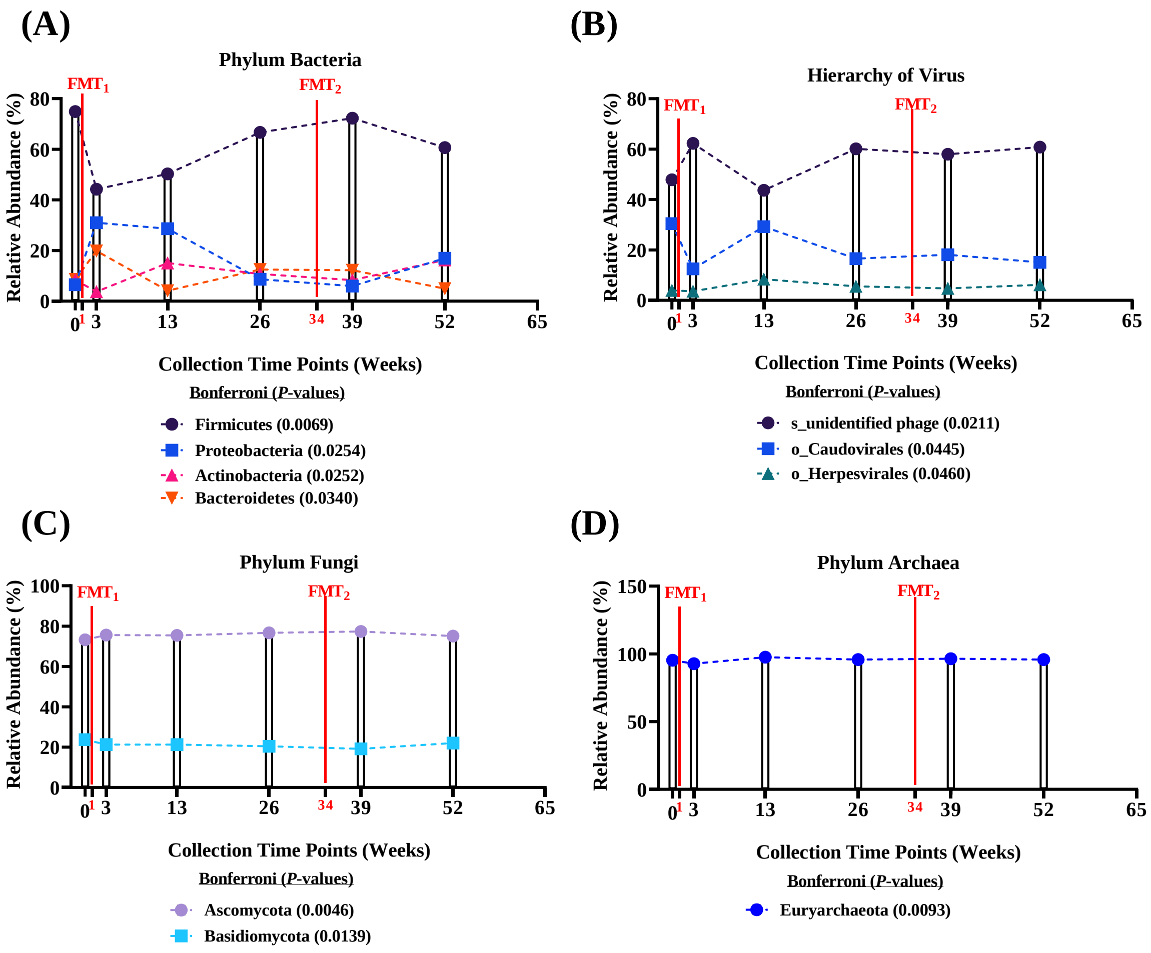


**Supplementary Figure 3.** Phyla significant movements across time of study. Differential shifts in significant percent relative abundances across time for phylum (A) bacteria, (B) virus, (C) fungi, and (D) archaea fecal microbiota of the RRMS subject's six collections time points (0, 3, 13, 26, 39, 52) in weeks. Significant Bonferroni P-values indicated. Directional mean trend dotted line shown across collection time points and FMT weeks.

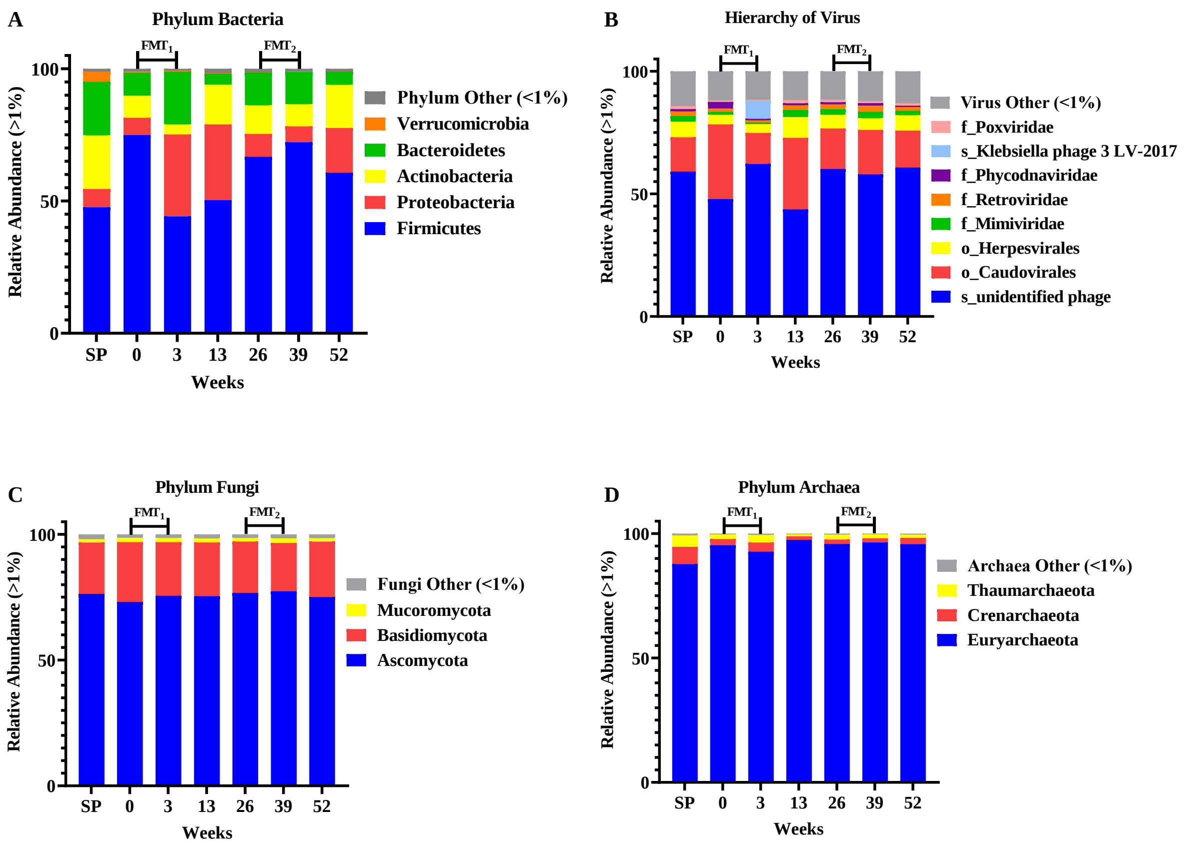


**Supplementary Figure 4.** Phylum taxonomic level microbiota profiles across time of study. Stacked column plots depicting the percent relative abundance (>1%) of phylum (A) bacteria, (B) virus, (C) fungi, and (D) archaea fecal microbiota of the household control spouse (SP) and the RRMS subject's six collections time points (0, 3, 13, 26, 39, 52) weeks. The first FMT ($FMT_1$) was between 0 and 3 weeks; second FMT ($FMT_2$) was between 26 and 39 weeks.

At the taxonomic level of species, the relative abundances of the RRMS subject were examined to determine which individual species (bacteria, viruses, archaea, and fungi) were affected by the FMT across all six fecal collection time points. The RRMS subject's overall fecal species microbial composition (bacteria, virus, archaea, and fungi) trajectory changes across time are depicted in (**Figure S5A-D**). Additionally, the RRMS subject's individual taxa species percent relative abundances (>1%), along with the observational standard household spouse control (SP), are shown across time for bacteria, virus, fungi and archaea (**Figure S6A-D**). The significant and trending species percent relative abundances within the RRMS subject across time are depicted for bacteria, virus, fungi, and archaea (**Figure S7A-D**).

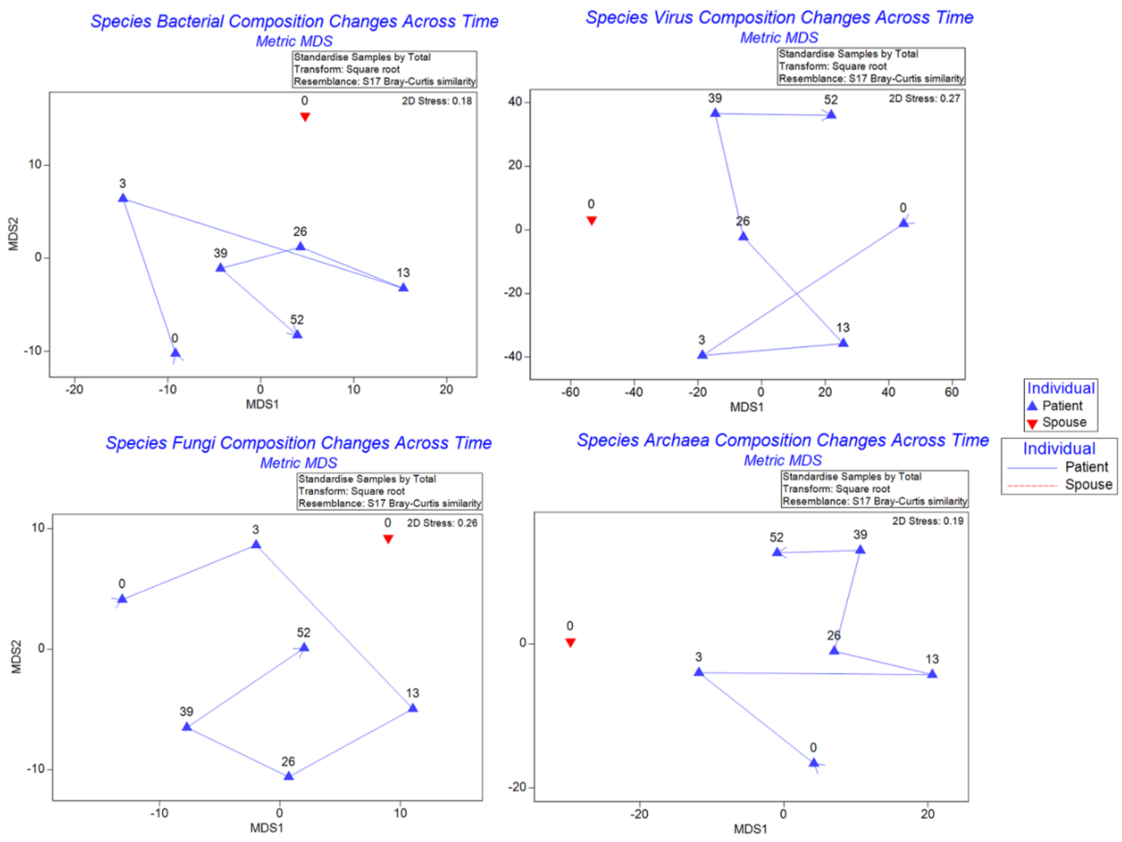


**Supplementary Figure 5.** Species overall composition trajectory changes across time of study. Metric-multidimensional scaling (mMDS) plots depicting the overall (A) bacteria, (B) virus, (C) fungi, and (D) archaea composition course changes across time, in the fecal microbiota of the household control spouse and the RRMS subject's six collections time points (0, 3, 13, 26, 39, 52) weeks. The first FMT ($FMT_1$) was between 0 and 3 weeks; second FMT ($FMT_2$) was between 26 and 39 weeks.

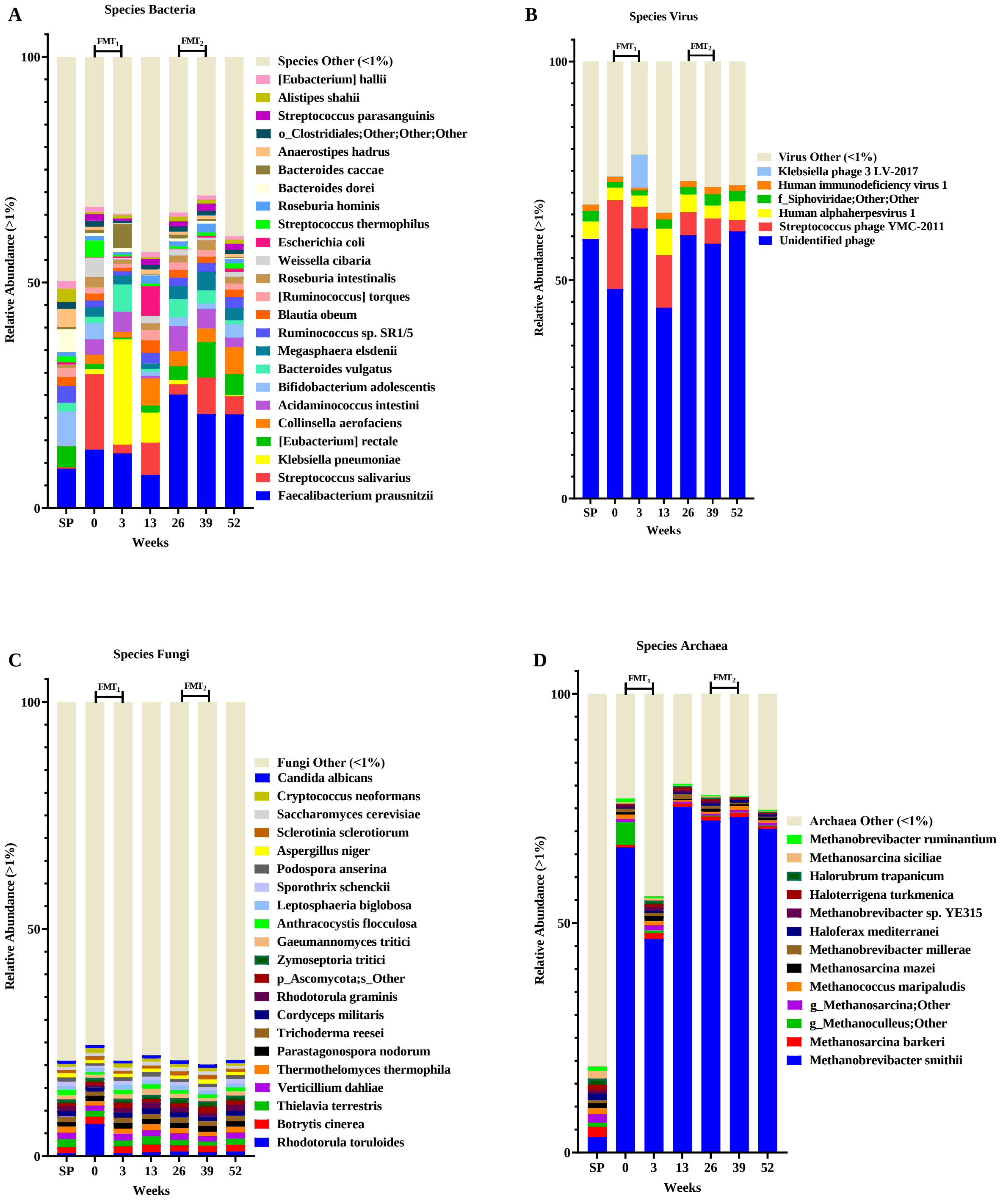


**Supplementary Figure 6.** Species taxonomic level microbiota profiles across time of study. Stacked column plots depicting the percent relative abundance (>1%) of species (A) bacteria, (B) virus, (C) fungi, and (D) archaea in the fecal microbiota of the household control spouse (SP) and the RRMS subject's six collections time points (0, 3, 13, 26, 39, 52) weeks. The first FMT ($FMT_1$) was between 0 and 3 weeks; second FMT ($FMT_2$) was between 26 and 39 weeks.

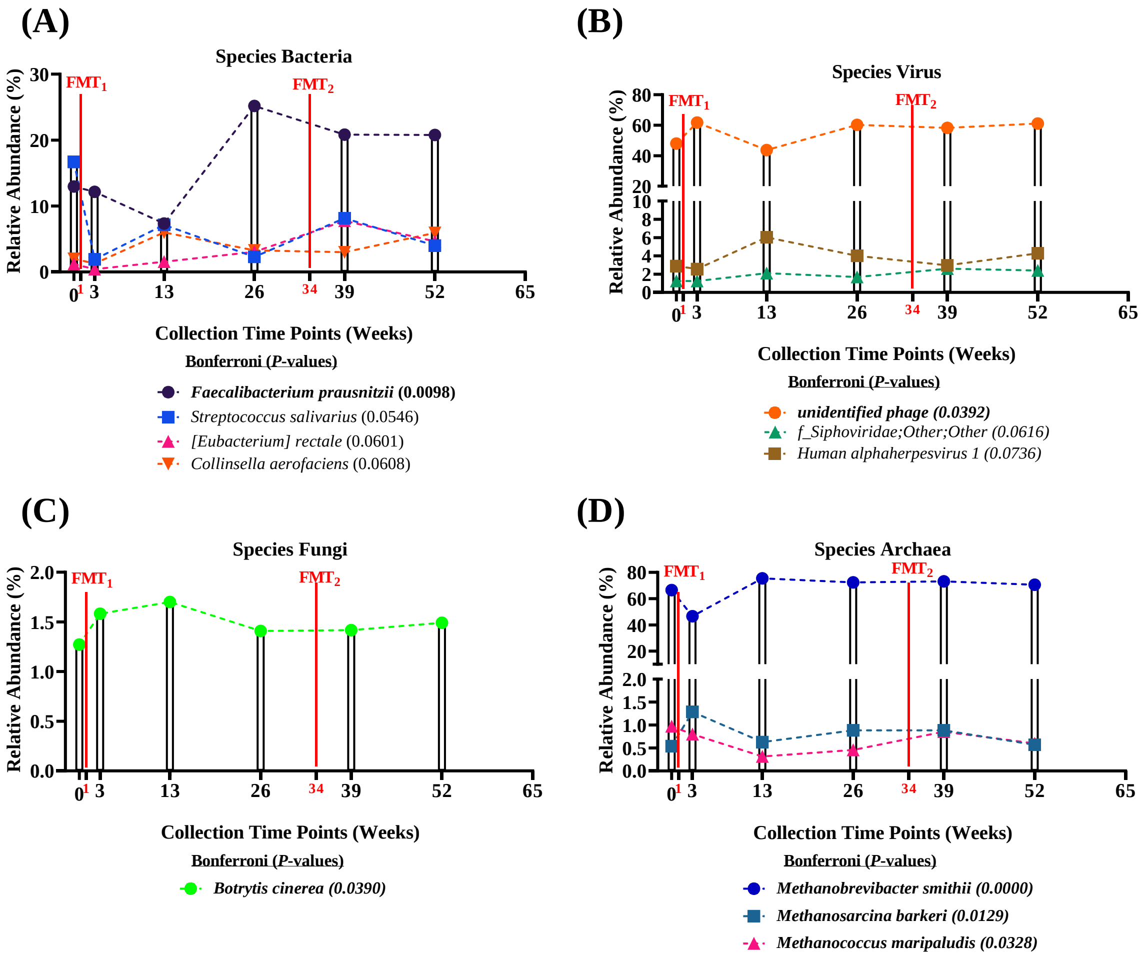


**Supplementary Figure 7.** Species significant movements across time of study. Differential shifts in significant percent relative abundances across time for species (A) bacteria, (B) virus, (C) fungi, and (D) archaea fecal microbiota of the RRMS subject's six collections time points (0, 3, 13, 26, 39, 52) weeks. Significant and trending Bonferroni *P*-values indicated. Directional mean trend dotted line shown across collection time points and FMT weeks.

Out of all individual bacterial taxa examined, only the butyrate-producing anti-inflammatory species *Faecalibacterium prausnitzii* (Bonferroni: *P*=0.0098), belonging to the phylum Firmicutes, had a significant increased relative abundance across the continuous variable of time (**Table S3, Figure S7A**). Bacterial species trending towards significance across time indicated higher relative abundances of butyrate-producing *Collinsella aerofaciens* (Bonferroni: *P*=0.0601) and *Eubacterium rectale* (Bonferroni: *P*=0.0608) in the RRMS subject. The relative abundance of species *Streptococcus salivarius* (Bonferroni *P*=0.0546) decreased from baseline, but then increased across time in the RRMS subject (**Figure S7A**). It should be noted, that following the first FMT ($FMT_1$), increased percent relative abundances of gram negative species *Klebsiella pneumoniae* at 3 and 13 weeks, as well as *Escherichia coli* at week 13 were discovered (**Figure S6A**). The RRMS subject did

not experience any adverse effects regarding intestinal problems at these two collection time points upon clinical review.

**Supplementary Table 3.** At the taxonomic level of species, *limma* powers differential abundance analyses across time for bacteria in the RRMS FMT subject.

| Bacterial Species | Average across time points | LFC | B-Stats | *P*-value | Bonferroni *P*-value | F | F *P*-value |
|---|---|---|---|---|---|---|---|
| *Faecalibacterium prausnitzii* | 17.4820 | 0.5063 | 3.8936 | **0.0001** | **0.0098** | 15.1602 | 0.0001 |
| *Streptococcus salivarius* | 15.8911 | 0.4475 | 3.4417 | **0.0006** | 0.0546 | 11.8451 | 0.0006 |
| *Eubacterium rectale* | 14.6208 | 0.4440 | 3.4149 | **0.0007** | 0.0601 | 11.6614 | 0.0006 |
| *Collinsella aerofaciens* | 15.1868 | 0.4436 | 3.4117 | **0.0007** | 0.0608 | 11.6396 | 0.0006 |
| *Megasphaera elsdenii* | 14.7800 | 0.4286 | 3.2964 | **0.0011** | 0.0912 | 10.8662 | 0.0010 |
| *Acidaminococcus intestine* | 15.0120 | 0.4277 | 3.2896 | **0.0011** | 0.0934 | 10.8215 | 0.0010 |
| *Ruminococcus sp. SR1/5* | 14.3672 | 0.4155 | 3.1958 | **0.0015** | 0.1287 | 10.2134 | 0.0014 |
| *Bifidobacterium adolescentis* | 13.8516 | 0.4081 | 3.1390 | **0.0018** | 0.1558 | 9.8534 | 0.0017 |
| *Roseburia intestinalis* | 14.2385 | 0.4065 | 3.1261 | **0.0019** | 0.1627 | 9.7725 | 0.0018 |
| *Bacteroides vulgatus* | 14.4707 | 0.4059 | 3.1217 | **0.0019** | 0.1651 | 9.7450 | 0.0018 |
| *Blautia obeum* | 14.1571 | 0.4044 | 3.1099 | **0.0020** | 0.1717 | 9.6715 | 0.0019 |
| *[Ruminococcus] torques* | 14.0591 | 0.4012 | 3.0858 | **0.0022** | 0.1858 | 9.5224 | 0.0020 |
| *Bifidobacterium bifidum* | 13.2080 | 0.3995 | 3.0724 | **0.0023** | 0.1942 | 9.4397 | 0.0021 |
| *Streptococcus parasanguinis* | 13.7559 | 0.3967 | 3.0512 | **0.0024** | 0.2082 | 9.3099 | 0.0023 |
| *Roseburia hominis* | 13.7006 | 0.3947 | 3.0353 | **0.0025** | 0.2192 | 9.2130 | 0.0024 |
| *o_Clostridiales; Other;Other; Other* | 13.5184 | 0.3852 | 2.9629 | **0.0032** | 0.2766 | 8.7789 | 0.0030 |
| *Weissella cibaria* | 13.6614 | 0.3818 | 2.9365 | **0.0035** | 0.3008 | 8.6230 | 0.0033 |
| *Alistipes shahii* | 12.9703 | 0.3788 | 2.9134 | **0.0038** | 0.3235 | 8.4879 | 0.0036 |
| *Streptococcus thermophiles* | 13.3998 | 0.3774 | 2.9023 | **0.0039** | 0.3349 | 8.4234 | 0.0037 |
| *[Eubacterium] hallii* | 13.1828 | 0.3763 | 2.8942 | **0.0040** | 0.3435 | 8.3766 | 0.0038 |
| *Ruminococcus bicirculans* | 13.3523 | 0.3727 | 2.8663 | **0.0044** | 0.3747 | 8.2158 | 0.0042 |
| *Anaerostipes hadrus* | 12.8256 | 0.3711 | 2.8541 | **0.0045** | 0.3891 | 8.1460 | 0.0043 |
| *Coprococcus catus* | 12.8940 | 0.3704 | 2.8490 | **0.0046** | 0.3953 | 8.1167 | 0.0044 |
| *f_Lachnospiraceae ;Other;Other* | 12.6173 | 0.3628 | 2.7904 | **0.0055** | 0.4729 | 7.7864 | 0.0053 |

| | | | | | | | |
|---|---|---|---|---|---|---|---|
| *[Eubacterium] eligens* | 12.8998 | 0.3614 | 2.7793 | **0.0057** | 0.4891 | 7.7244 | 0.0054 |
| *Butyrate-producing bacterium SS3/4* | 12.4038 | 0.3577 | 2.7508 | **0.0062** | 0.5329 | 7.5668 | 0.0059 |
| *Escherichia coli* | 12.2183 | 0.3550 | 2.7300 | **0.0066** | 0.5671 | 7.4528 | 0.0063 |
| *Bifidobacterium longum* | 11.2629 | 0.3439 | 2.6450 | **0.0085** | 0.7283 | 6.9958 | 0.0082 |
| *Bacteroides caccae* | 12.8255 | 0.3431 | 2.6388 | **0.0086** | 0.7415 | 6.9632 | 0.0083 |
| *Bacteroides dorei* | 12.4010 | 0.3428 | 2.6365 | **0.0087** | 0.7464 | 6.9512 | 0.0084 |
| *p_Firmicutes; Other;Other; Other;Other; Other* | 12.0542 | 0.3428 | 2.6363 | **0.0087** | 0.7469 | 6.9502 | 0.0084 |
| *Desulfovibrio piger* | 11.6287 | 0.3419 | 2.6299 | **0.0088** | 0.7609 | 6.9163 | 0.0085 |
| *Coprococcus sp. ART55/1* | 11.6875 | 0.3416 | 2.6272 | **0.0089** | 0.7668 | 6.9022 | 0.0086 |
| *g_Bacteroides; Other* | 12.2760 | 0.3408 | 2.6215 | **0.0091** | 0.7796 | 6.8721 | 0.0088 |
| *Odoribacter splanchnicus* | 11.8764 | 0.3400 | 2.6149 | **0.0092** | 0.7945 | 6.8378 | 0.0089 |
| *p_Proteobacteria; Other;Other; Other;Other; Other* | 11.7692 | 0.3365 | 2.5877 | **0.0100** | 0.8590 | 6.6963 | 0.0097 |
| *[Eubacterium] siraeum* | 11.6291 | 0.3358 | 2.5823 | **0.0101** | 0.8723 | 6.6684 | 0.0098 |
| *Bacteroides ovatus* | 12.0265 | 0.3339 | 2.5684 | **0.0106** | 0.9075 | 6.5968 | 0.0102 |
| *Intestinimonas butyriciproducens* | 11.6206 | 0.3313 | 2.5484 | **0.0112** | 0.9605 | 6.4942 | 0.0108 |
| *Bacteroides fragilis* | 11.7604 | 0.3302 | 2.5397 | **0.0114** | 0.9843 | 6.4501 | 0.0111 |
| *Klebsiella pneumoniae* | 13.5083 | 0.3270 | 2.5150 | **0.0123** | 1.0000 | 6.3252 | 0.0119 |
| *Flavonifractor plautii* | 11.3463 | 0.3261 | 2.5079 | **0.0125** | 1.0000 | 6.2897 | 0.0121 |
| *g_Streptococcus; Other* | 11.6146 | 0.3252 | 2.5010 | **0.0128** | 1.0000 | 6.2551 | 0.0124 |
| *o_Bacteroidales; Other;Other; Other* | 11.6287 | 0.3249 | 2.4988 | **0.0128** | 1.0000 | 6.2442 | 0.0125 |
| *Bacteroides cellulosilyticus* | 11.2440 | 0.3245 | 2.4960 | **0.0129** | 1.0000 | 6.2299 | 0.0126 |
| *Clostridioides difficile* | 11.2239 | 0.3235 | 2.4878 | **0.0132** | 1.0000 | 6.1890 | 0.0129 |
| *[Clostridium] bolteae* | 11.2670 | 0.3217 | 2.4740 | **0.0137** | 1.0000 | 6.1206 | 0.0134 |
| *Faecalitalea cylindroides* | 11.2585 | 0.3201 | 2.4622 | **0.0142** | 1.0000 | 6.0624 | 0.0138 |
| *Bacteroides thetaiotaomicron* | 11.4936 | 0.3178 | 2.4441 | **0.0149** | 1.0000 | 5.9734 | 0.0145 |
| *[Clostridium] saccharolyticum* | 11.0059 | 0.3171 | 2.4385 | **0.0152** | 1.0000 | 5.9463 | 0.0147 |

| | | | | | | | |
|---|---|---|---|---|---|---|---|
| *Alistipes finegoldii* | 11.2678 | 0.3160 | 2.4306 | **0.0155** | 1.0000 | 5.9078 | 0.0151 |
| *Lachnoclostridium phocaeense* | 11.0474 | 0.3159 | 2.4294 | **0.0155** | 1.0000 | 5.9018 | 0.0151 |
| *Blautia hansenii* | 11.0573 | 0.3158 | 2.4285 | **0.0156** | 1.0000 | 5.8975 | 0.0152 |
| *c_Actinobacteria; Other;Other; Other; Other* | 10.9243 | 0.3144 | 2.4177 | **0.0160** | 1.0000 | 5.8453 | 0.0156 |
| *Bacteroides xylanisolvens* | 11.0683 | 0.3119 | 2.3986 | **0.0169** | 1.0000 | 5.7533 | 0.0165 |
| *Streptococcus mutans* | 11.6814 | 0.3102 | 2.3861 | **0.0175** | 1.0000 | 5.6935 | 0.0170 |
| *f_Enterobacteriac ea;Other;Other* | 11.3015 | 0.3082 | 2.3702 | **0.0182** | 1.0000 | 5.6176 | 0.0178 |
| *Streptococcus anginosus* | 10.6579 | 0.3073 | 2.3634 | **0.0186** | 1.0000 | 5.5858 | 0.0181 |
| *g_Enterobacter; Other* | 10.4115 | 0.3065 | 2.3577 | **0.0188** | 1.0000 | 5.5586 | 0.0184 |
| *g_Blautia;Other* | 10.6019 | 0.3047 | 2.3437 | **0.0195** | 1.0000 | 5.4930 | 0.0191 |
| *g_Parabacteroides ;Other* | 10.9017 | 0.3023 | 2.3248 | **0.0205** | 1.0000 | 5.4049 | 0.0201 |
| *Bacteroides caecimuris* | 10.8622 | 0.3014 | 2.3178 | **0.0209** | 1.0000 | 5.3722 | 0.0205 |
| *Oscillibacter valericigenes* | 10.4317 | 0.2994 | 2.3030 | **0.0218** | 1.0000 | 5.3037 | 0.0213 |
| *Klebsiella oxytoca* | 9.2889 | 0.2988 | 2.2983 | **0.0220** | 1.0000 | 5.2822 | 0.0215 |
| *Romboutsia ilealis* | 10.2165 | 0.2980 | 2.2920 | **0.0224** | 1.0000 | 5.2532 | 0.0219 |
| *Eggerthella lenta* | 10.2427 | 0.2979 | 2.2911 | **0.0224** | 1.0000 | 5.2489 | 0.0220 |
| *g_Bifidobacterium ;Other* | 10.1200 | 0.2955 | 2.2724 | **0.0236** | 1.0000 | 5.1640 | 0.0231 |
| *g_Klebsiella; Other* | 11.0541 | 0.2928 | 2.2516 | **0.0248** | 1.0000 | 5.0698 | 0.0243 |
| *g_Streptomyces; Other* | 10.1454 | 0.2917 | 2.2437 | **0.0254** | 1.0000 | 5.0341 | 0.0249 |
| *g_Pseudomonas; Other* | 10.0908 | 0.2909 | 2.2372 | **0.0258** | 1.0000 | 5.0051 | 0.0253 |
| *Ruminococcus champanellensis* | 10.2316 | 0.2905 | 2.2340 | **0.0260** | 1.0000 | 4.9906 | 0.0255 |
| *s_Prevotella intermedia* | 10.2194 | 0.2904 | 2.2334 | **0.0260** | 1.0000 | 4.9883 | 0.0255 |
| *g_Burkholderia; Other* | 10.0665 | 0.2894 | 2.2255 | **0.0266** | 1.0000 | 4.9529 | 0.0260 |
| *Prevotella ruminicola* | 10.0044 | 0.2859 | 2.1989 | **0.0284** | 1.0000 | 4.8353 | 0.0279 |
| *Streptococcus gordonii* | 10.0240 | 0.2838 | 2.1825 | **0.0296** | 1.0000 | 4.7632 | 0.0291 |
| *c_Gammaproteob acteria;Other; Other;Other; Other* | 10.0952 | 0.2832 | 2.1782 | **0.0299** | 1.0000 | 4.7447 | 0.0294 |
| *Enterobacter cloacae* | 9.4693 | 0.2828 | 2.1751 | **0.0302** | 1.0000 | 4.7311 | 0.0296 |

| | | | | | | | |
|---|---|---|---|---|---|---|---|
| *Parabacteroides sp. CT06* | 10.2644 | 0.2818 | 2.1676 | **0.0307** | 1.0000 | 4.6984 | 0.0302 |
| *o_Lactobacillales; Other;Other; Other* | 10.1888 | 0.2808 | 2.1596 | **0.0314** | 1.0000 | 4.6641 | 0.0308 |
| *Barnesiella viscericola* | 10.0751 | 0.2790 | 2.1459 | **0.0324** | 1.0000 | 4.6050 | 0.0319 |
| *Parabacteroides distasonis* | 10.1730 | 0.2774 | 2.1335 | **0.0335** | 1.0000 | 4.5518 | 0.0329 |
| *o_Enterobacterale ;Other;Other;Othe r* | 9.7087 | 0.2647 | 2.0356 | **0.0424** | 1.0000 | 4.1435 | 0.0418 |
| *Akkermansia muciniphila* | 9.9361 | 0.2465 | 1.8957 | 0.0587 | 1.0000 | 3.5938 | 0.0580 |
| LFC = log fold change. *limma* software R program. Log2 fold change calculated for each time point. Weeks 0, 3, 13, 26, 39, 52 were examined across six time points. Bacterial abundance filtered at (>0.1%) for analysis. Taxonomic annotations: p = phylum; o = order; c = class; f = family; g = genus. | | | | | | | |

Furthermore, the percent relative abundances of individual bacterial species observational changes for the RRMS subject week 0 (baseline) against week 52 (end of study) taxa profiles (>1%) are indicated (**Figure S8A**). Also, the bacterial species observational changes for the household spouse control compared to the RRMS subject week 52 are shown (**Figure S8B**).

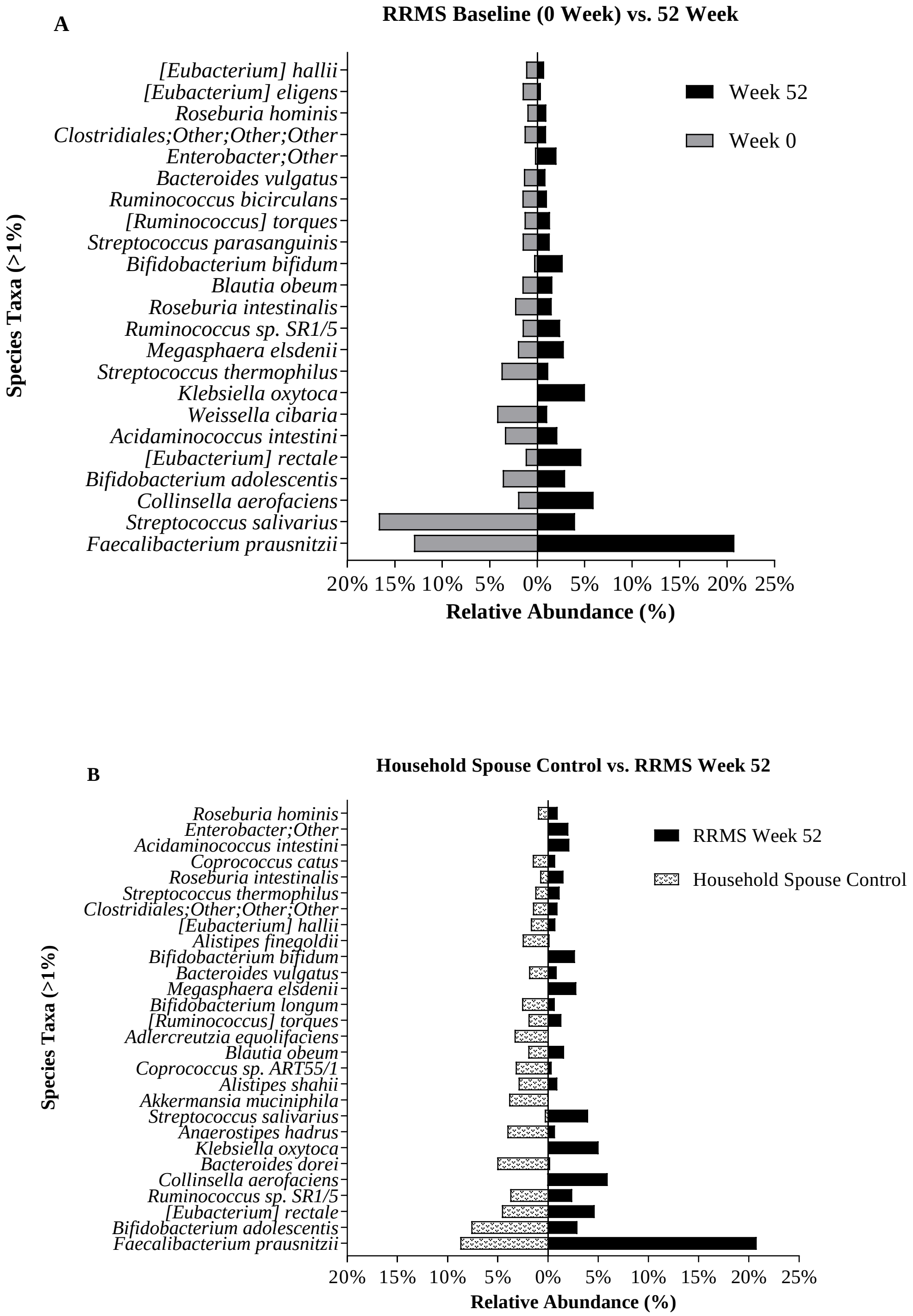


**Supplementary Figure 8.** Bacterial profiles of household spouse control and RRMS subject's baseline and end of study samples. Bar plots depict observational changes of the percent relative abundance (>1%) in species bacteria in the fecal microbiota between (A) RRMS week 0 (baseline) vs. week 52 (end of study); and (B) household spouse control and the RRMS week 52 collection time points.

For cross-reference to previously published RRMS scientific microbiota data, significant different trends in relative abundances across time, at the taxonomic level of genus, are depicted for taxa annotation comparisons to 16S ribosomal RNA (rRNA) sequencing data (**Table S4**). Genus *Faecalibacterium* (Bonferroni *P*=0.0105) had a significant increased relative abundance across time. Genus *Streptococcus* (Bonferroni *P*=0.0281) relative abundance decreased from baseline and then significantly increased across the continuous variable of time.

**Supplementary Table 4**. At the taxonomic level of genus, *limma* powers differential abundance analyses across time for bacteria in the RRMS FMT subject.

| Bacterial Genera | Average across time points | LFC | B-Stats | *P*-value | Bonferroni *P*-value | F | F *P*-value |
|---|---|---|---|---|---|---|---|
| *Faecalibacterium* | 17.4807 | 0.5062 | 3.8296 | **0.0002** | **0.0105** | 14.6656 | 0.0001 |
| *Streptococcus* | 16.7052 | 0.4719 | 3.5701 | **0.0004** | **0.0281** | 12.7454 | 0.0004 |
| *Klebsiella* | 14.6116 | 0.3898 | 2.9492 | **0.0034** | 0.2349 | 8.6975 | 0.0032 |
| *Bacteroides* | 15.9399 | 0.4439 | 3.3584 | **0.0009** | 0.0601 | 11.2787 | 0.0008 |
| *Collinsella* | 15.1856 | 0.4435 | 3.3553 | **0.0009** | 0.0608 | 11.2581 | 0.0008 |
| *Bifidobacterium* | 15.0046 | 0.4410 | 3.3362 | **0.0009** | 0.0650 | 11.1302 | 0.0008 |
| *Acidaminococcus* | 15.0391 | 0.4285 | 3.2415 | **0.0013** | 0.0900 | 10.5076 | 0.0012 |
| *Blautia* | 15.2678 | 0.4358 | 3.2968 | **0.0011** | 0.0745 | 10.8687 | 0.0010 |
| *Eubacterium* | 14.6249 | 0.4440 | 3.3592 | **0.0009** | 0.0600 | 11.2841 | 0.0008 |
| *Ruminococcus* | 15.1265 | 0.4312 | 3.2619 | **0.0012** | 0.0840 | 10.6402 | 0.0011 |
| *Roseburia* | 15.0414 | 0.4303 | 3.2554 | **0.0012** | 0.0859 | 10.5977 | 0.0011 |
| *Megasphaera* | 14.7804 | 0.4286 | 3.2422 | **0.0013** | 0.0898 | 10.5119 | 0.0012 |
| *Weissella* | 13.9309 | 0.3888 | 2.9415 | **0.0035** | 0.2406 | 8.6522 | 0.0033 |
| *Eubacterium* | 14.1888 | 0.4015 | 3.0374 | **0.0026** | 0.1772 | 9.2258 | 0.0024 |
| *Escherichia* | 12.2846 | 0.3564 | 2.6965 | **0.0074** | 0.5072 | 7.2712 | 0.0070 |
| *Enterococcus* | 11.6336 | 0.3020 | 2.2843 | **0.0230** | 1.0000 | 5.2180 | 0.0224 |
| *o_Clostridiales; Other;Other* | 13.5144 | 0.3851 | 2.9130 | **0.0038** | 0.2631 | 8.4857 | 0.0036 |
| *Coprococcus* | 13.4371 | 0.3878 | 2.9334 | **0.0036** | 0.2468 | 8.6051 | 0.0034 |
| *Alistipes* | 13.4294 | 0.3881 | 2.9356 | **0.0036** | 0.2451 | 8.6179 | 0.0033 |
| *Lachnoclostridium* | 13.1724 | 0.3761 | 2.8454 | **0.0047** | 0.3244 | 8.0962 | 0.0044 |
| *Enterobacter* | 11.6089 | 0.3407 | 2.5777 | **0.0104** | 0.7148 | 6.6446 | 0.0099 |
| *Anaerostipes* | 12.8322 | 0.3712 | 2.8081 | **0.0053** | 0.3635 | 7.8852 | 0.0050 |
| *Prevotella* | 12.7731 | 0.3637 | 2.7515 | **0.0062** | 0.4309 | 7.5709 | 0.0059 |
| *f_Enterobacteriaceae; Other* | 11.2933 | 0.3080 | 2.3299 | **0.0204** | 1.0000 | 5.4287 | 0.0198 |
| *f_Lachnospiraceae; Other* | 12.6101 | 0.3629 | 2.7456 | **0.0064** | 0.4386 | 7.5384 | 0.0060 |
| *Streptomyces* | 12.5090 | 0.3590 | 2.7156 | **0.0069** | 0.4795 | 7.3744 | 0.0066 |
| *Parabacteroides* | 12.0986 | 0.3348 | 2.5329 | **0.0118** | 0.8111 | 6.4155 | 0.0113 |
| *Unclassified Clostridiales* | 12.4035 | 0.3579 | 2.7073 | **0.0071** | 0.4913 | 7.3295 | 0.0068 |
| *Pseudomonas* | 12.3698 | 0.3549 | 2.6851 | **0.0076** | 0.5245 | 7.2096 | 0.0073 |
| *Clostridium* | 12.2965 | 0.3498 | 2.6459 | **0.0085** | 0.5879 | 7.0009 | 0.0081 |

| | | | | | | | |
|---|---|---|---|---|---|---|---|
| *Lactobacillus* | 12.1548 | 0.3426 | 2.5914 | **0.0100** | 0.6875 | 6.7154 | 0.0096 |
| *Ruminiclostridium* | 12.1144 | 0.3489 | 2.6395 | **0.0087** | 0.5989 | 6.9668 | 0.0083 |
| *Desulfovibrio* | 12.1126 | 0.3535 | 2.6739 | **0.0079** | 0.5420 | 7.1495 | 0.0075 |
| *p_Firmicutes;Other; Other;Other;Other* | 12.0624 | 0.3431 | 2.5954 | **0.0099** | 0.6797 | 6.7363 | 0.0094 |
| *Odoribacter* | 11.8601 | 0.3392 | 2.5663 | **0.0107** | 0.7384 | 6.5858 | 0.0103 |
| *Bacillus* | 11.8917 | 0.3385 | 2.5611 | **0.0109** | 0.7492 | 6.5593 | 0.0104 |
| *p_Proteobacteria; Other;Other;Other; Other* | 11.7541 | 0.3356 | 2.5390 | **0.0116** | 0.7974 | 6.4464 | 0.0111 |
| *Selenomonas* | 11.6575 | 0.3455 | 2.6140 | **0.0093** | 0.6444 | 6.8332 | 0.0089 |
| *Burkholderia* | 11.7139 | 0.3361 | 2.5429 | **0.0114** | 0.7886 | 6.4666 | 0.0110 |
| *Paenibacillus* | 11.7675 | 0.3376 | 2.5538 | **0.0111** | 0.7648 | 6.5220 | 0.0107 |
| *o_Bacteroidales; Other;Other* | 11.6237 | 0.3246 | 2.4556 | **0.0146** | 1.0000 | 6.0301 | 0.0141 |
| *Pediococcus* | 9.8071 | 0.2436 | 1.8432 | **0.0662** | 1.0000 | 3.3973 | 0.0653 |
| *Intestinimonas* | 11.6205 | 0.3317 | 2.5089 | **0.0126** | 0.8671 | 6.2948 | 0.0121 |
| *Flavonifractor* | 11.3516 | 0.3264 | 2.4694 | **0.0140** | 0.9672 | 6.0980 | 0.0135 |
| *Faecalitalea* | 11.2720 | 0.3207 | 2.4262 | **0.0158** | 1.0000 | 5.8865 | 0.0153 |
| *Clostridioides* | 11.2344 | 0.3238 | 2.4496 | **0.0148** | 1.0000 | 6.0008 | 0.0143 |
| *Shigella* | 7.6784 | 0.2139 | 1.6181 | **0.1066** | 1.0000 | 2.6181 | 0.1056 |
| *Gordonibacter* | 11.0176 | 0.3220 | 2.4362 | **0.0153** | 1.0000 | 5.9351 | 0.0148 |
| *Citrobacter* | 10.0237 | 0.3052 | 2.3089 | **0.0215** | 1.0000 | 5.3312 | 0.0209 |
| *Mycobacterium* | 10.9801 | 0.3136 | 2.3725 | **0.0182** | 1.0000 | 5.6287 | 0.0177 |
| *Olsenella* | 10.8982 | 0.3227 | 2.4416 | **0.0151** | 1.0000 | 5.9613 | 0.0146 |
| *Akkermansia* | 10.1093 | 0.2535 | 1.9176 | **0.0560** | 1.0000 | 3.6771 | 0.0552 |
| *c_Actinobacteria; Other;Other;Other* | 10.9129 | 0.3139 | 2.3749 | **0.0181** | 1.0000 | 5.6401 | 0.0176 |
| *Corynebacterium* | 10.6071 | 0.3048 | 2.3060 | **0.0217** | 1.0000 | 5.3175 | 0.0211 |
| *Eggerthella* | 10.4716 | 0.3042 | 2.3015 | **0.0220** | 1.0000 | 5.2968 | 0.0214 |
| *Romboutsia* | 10.2234 | 0.2979 | 2.2538 | **0.0248** | 1.0000 | 5.0794 | 0.0242 |
| *o_Lactobacillales; Other;Other* | 10.1758 | 0.2803 | 2.1204 | **0.0347** | 1.0000 | 4.4963 | 0.0340 |
| *Leuconostoc* | 10.1839 | 0.2983 | 2.2563 | **0.0247** | 1.0000 | 5.0907 | 0.0241 |
| *Butyrivibrio* | 10.5123 | 0.3036 | 2.2967 | **0.0222** | 1.0000 | 5.2748 | 0.0216 |
| *Oscillibacter* | 10.4307 | 0.2993 | 2.2642 | **0.0242** | 1.0000 | 5.1267 | 0.0236 |
| *Lactococcus* | 9.7249 | 0.2557 | 1.9341 | **0.0539** | 1.0000 | 3.7409 | 0.0531 |
| *Ralstonia* | 10.2351 | 0.2867 | 2.1692 | **0.0307** | 1.0000 | 4.7054 | 0.0301 |
| *Actinomyces* | 10.2106 | 0.3002 | 2.2713 | **0.0237** | 1.0000 | 5.1586 | 0.0231 |
| *Staphylococcus* | 10.3234 | 0.2948 | 2.2304 | **0.0264** | 1.0000 | 4.9747 | 0.0257 |
| *o_Enterobacterales; Other;Other* | 9.7014 | 0.2641 | 1.9978 | **0.0465** | 1.0000 | 3.9914 | 0.0457 |
| *Campylobacter* | 10.1419 | 0.2855 | 2.1598 | **0.0315** | 1.0000 | 4.6649 | 0.0308 |
| *c_Gammaproteobacteri a;Other;Other;Other* | 10.0875 | 0.2835 | 2.1444 | **0.0327** | 1.0000 | 4.5984 | 0.0320 |

| | | | | | | | |
|---|---|---|---|---|---|---|---|
| *Barnesiella* | 10.1166 | 0.2810 | 2.1256 | **0.0343** | 1.0000 | 4.5180 | 0.0335 |
| *Bradyrhizobium* | 10.0772 | 0.2896 | 2.1911 | **0.0291** | 1.0000 | 4.8010 | 0.0284 |
| LFC = log fold change. *limma* software R program. Log2 fold change calculated for each time point. Weeks 0, 3, 13, 26, 39, 52 were examined across six time points. Bacterial abundance filtered at (>0.1%) for analysis. Taxonomic annotations: p = phylum; o = order; c = class; f = family. | | | | | | | |

Similar to species bacteria, only one virus species identified as *Unidentified phage* (Bonferroni: *P*=0.0392), significantly increased across time in the RRMS subject (**Table S5, Figure S7B**). Species viruses trending towards significance across time indicated higher relative abundances of *Siphoviridae;Other;Other* (Bonferroni: *P*=0.0616) and *Human alphaherpesvirus 1* (Bonferroni: *P*=0.0736) (**Figure S7B**). It should be noted, that following $FMT_1$, an increased percent relative abundance of bacteriophage virus *Klebsiella phage 3 LV-2017* at 3 weeks was shown (**Figure S6B**). Interestingly, this is in accordance with the high percent relative abundance of the gram negative bacterial species *Klebsiella pneumoniae* at 3 weeks (**Figure S6A**).

**Supplementary Table 5.** At the taxonomic level of species, *limma* powers differential abundance analyses across time for viruses in the RRMS FMT subject.

| Virus Species | Average across time points | LFC | B-Stats | *P*-value | Bonferroni *P*-value | F | F *P*-value |
|---|---|---|---|---|---|---|---|
| *Unidentified phage* | 10.9119 | 0.3123 | 4.2160 | **0.0008** | **0.0392** | 17.7745 | 0.0008 |
| *f_Siphoviridae;Other; Other* | 5.9852 | 0.1786 | 3.9862 | **0.0013** | 0.0616 | 15.8900 | 0.0013 |
| *Human alphaherpesvirus-1* | 6.9883 | 0.2013 | 3.8964 | **0.0016** | 0.0736 | 15.1823 | 0.0016 |
| *Human immunodeficiency virus-1* | 5.4182 | 0.1602 | 3.7452 | **0.0021** | 0.0995 | 14.0266 | 0.0021 |
| *Streptococcus phage 20617* | 3.6431 | 0.1204 | 3.4955 | **0.0035** | 0.1639 | 12.2186 | 0.0035 |
| *f_Myoviridae;Other;Other* | 4.4968 | 0.1333 | 3.4358 | **0.0039** | 0.1848 | 11.8044 | 0.0039 |
| *Streptococcus phage YMC-2011* | 7.8899 | 0.2070 | 3.3206 | **0.0050** | 0.2327 | 11.0267 | 0.0050 |
| *Bifidobacterium phage Bbif-1* | 2.6912 | 0.1014 | 3.3028 | **0.0051** | 0.2412 | 10.9082 | 0.0051 |
| *f_Mimiviridae;Other; Other* | 3.7518 | 0.1127 | 3.1803 | **0.0066** | 0.3083 | 10.1143 | 0.0066 |
| *Gokushovirus WZ-2015a* | 3.3633 | 0.1106 | 3.0904 | **0.0079** | 0.3691 | 9.5509 | 0.0079 |
| *Streptococcus phage 5093* | 2.7358 | 0.0997 | 3.0364 | **0.0087** | 0.4111 | 9.2199 | 0.0087 |
| *Megavirus chiliensis* | 3.0640 | 0.0976 | 3.0043 | **0.0093** | 0.4384 | 9.0257 | 0.0093 |
| *Influenza A virus* | 2.6295 | 0.0890 | 2.8712 | **0.0122** | 0.5714 | 8.2439 | 0.0122 |
| *Bubaline alphaherpesvirus-1* | 2.6457 | 0.0859 | 2.7702 | **0.0149** | 0.6980 | 7.6742 | 0.0149 |
| *Moumouvirus* | 2.5765 | 0.0818 | 2.6062 | **0.0205** | 0.9639 | 6.7923 | 0.0205 |
| *Pandoravirus inopinatum* | 2.2426 | 0.0770 | 2.5659 | **0.0222** | 1.0000 | 6.5840 | 0.0222 |
| *Hepacivirus C* | 2.7122 | 0.0815 | 2.5517 | **0.0228** | 1.0000 | 6.5113 | 0.0228 |
| *Hokovirus HKV1* | 2.4486 | 0.0801 | 2.5159 | **0.0245** | 1.0000 | 6.3299 | 0.0245 |
| *Klosneuvirus KNV1* | 2.4962 | 0.0787 | 2.5055 | **0.0250** | 1.0000 | 6.2777 | 0.0250 |
| *Streptococcus phage P7132* | 1.2335 | 0.0718 | 2.4987 | **0.0253** | 1.0000 | 6.2436 | 0.0253 |
| *Catovirus CTV1* | 2.1386 | 0.0758 | 2.4837 | **0.0260** | 1.0000 | 6.1689 | 0.0260 |

| | | | | | | | |
|---|---|---|---|---|---|---|---|
| *Simian immunodeficiency virus* | 1.7925 | 0.0679 | 2.3895 | **0.0312** | 1.0000 | 5.7097 | 0.0312 |
| *Rotavirus A* | 2.4096 | 0.0730 | 2.3732 | **0.0322** | 1.0000 | 5.6320 | 0.0322 |
| *Vibrio phage JSF17* | 3.1336 | 0.0849 | 2.3357 | **0.0346** | 1.0000 | 5.4557 | 0.0346 |
| *o_Caudovirales;Other; Other;Other* | 2.1025 | 0.0688 | 2.2393 | **0.0416** | 1.0000 | 5.0146 | 0.0416 |
| *Pandoravirus salinus* | 2.2233 | 0.0676 | 2.2162 | **0.0435** | 1.0000 | 4.9117 | 0.0435 |
| *Pandoravirus dulcis* | 1.9099 | 0.0630 | 2.0766 | 0.0564 | 1.0000 | 4.3121 | 0.0564 |

LFC = log fold change. *limma* software R program. Log2 fold change calculated for each time point. Weeks 0, 3, 13, 26, 39, 52 were examined across six time points. Virus abundance filtered at (>0.1%) for analysis. Taxonomic level classifications: o = order, f = family, g = genus.

One fungal species *Botrytis cinereal* (Bonferroni: P=0.0390) had significant increased movements across time (**Table S6, Figure S7C**). Of note, fungi species *Rhodotorula toruloides* was highly abundant at week 0 (baseline), in the RRMS subject. Following $FMT_1$, the relative abundance of *Rhodotorula toruloides* decreased from baseline (**Figure S6C**).

**Supplementary Table 6.** At the taxonomic level of species, *limma* powers differential abundance analyses across time for fungi in the RRMS FMT subject.

| Fungi Species | Average across time points | LFC | B-Stats | *P*-value | Bonferroni *P*-value | F | F *P*-value |
|---|---|---|---|---|---|---|---|
| *Botrytis cinerea* | 7.3807 | 0.2085 | 3.7994 | **0.0002** | **0.0390** | 14.4355 | 0.0001 |
| *Verticillium dahliae* | 7.1690 | 0.2037 | 3.7117 | **0.0002** | 0.0552 | 13.7765 | 0.0002 |
| *Thielavia terrestris* | 7.1439 | 0.2010 | 3.6618 | **0.0003** | 0.0670 | 13.4088 | 0.0003 |
| *Parastagonospora nodorum* | 7.0409 | 0.2005 | 3.6524 | **0.0003** | 0.0695 | 13.3400 | 0.0003 |
| *Trichoderma reesei* | 6.9125 | 0.1984 | 3.6142 | **0.0003** | 0.0804 | 13.0623 | 0.0003 |
| *Thermothelomyces thermophila* | 6.9569 | 0.1981 | 3.6084 | **0.0003** | 0.0822 | 13.0208 | 0.0003 |
| *Cordyceps militaris* | 6.8945 | 0.1960 | 3.5717 | **0.0004** | 0.0945 | 12.7569 | 0.0004 |
| *Rhodotorula graminis* | 6.6826 | 0.1957 | 3.5659 | **0.0004** | 0.0966 | 12.7153 | 0.0004 |
| *p_Ascomycota;Other;Other;Other;Other;Other* | 6.7809 | 0.1953 | 3.5579 | **0.0004** | 0.0996 | 12.6588 | 0.0004 |
| *Zymoseptoria tritici* | 6.7460 | 0.1952 | 3.5556 | **0.0004** | 0.1005 | 12.6421 | 0.0004 |
| *Rhodotorula toruloides* | 7.1089 | 0.1904 | 3.4695 | **0.0005** | 0.1385 | 12.0372 | 0.0005 |
| *Gaeumannomyces tritici* | 6.6467 | 0.1881 | 3.4265 | **0.0006** | 0.1621 | 11.7406 | 0.0006 |
| *Leptosphaeria biglobosa* | 6.6359 | 0.1868 | 3.4032 | **0.0007** | 0.1764 | 11.5818 | 0.0007 |
| *Sporothrix schenckii* | 6.4036 | 0.1867 | 3.4016 | **0.0007** | 0.1775 | 11.5707 | 0.0007 |
| *Anthracocystis flocculosa* | 6.5081 | 0.1867 | 3.4011 | **0.0007** | 0.1778 | 11.5677 | 0.0007 |
| *Sclerotinia sclerotiorum* | 6.4385 | 0.1839 | 3.3500 | **0.0008** | 0.2137 | 11.2227 | 0.0008 |
| *Podospora anserina* | 6.4134 | 0.1833 | 3.3392 | **0.0009** | 0.2222 | 11.1500 | 0.0008 |
| *Aspergillus niger* | 6.4100 | 0.1832 | 3.3374 | **0.0009** | 0.2235 | 11.1385 | 0.0008 |
| *Saccharomyces cerevisiae* | 6.3827 | 0.1805 | 3.2882 | **0.0010** | 0.2661 | 10.8124 | 0.0010 |
| *Candida albicans* | 6.2577 | 0.1797 | 3.2740 | **0.0011** | 0.2798 | 10.7189 | 0.0011 |
| *Diplodia corticola* | 6.1348 | 0.1792 | 3.2653 | **0.0011** | 0.2885 | 10.6619 | 0.0011 |

| | | | | | | | |
|---|---|---|---|---|---|---|---|
| *Saccharomyces paradoxus* | 6.1534 | 0.1771 | 3.2263 | **0.0013** | 0.3304 | 10.4093 | 0.0013 |
| *Cryptococcus neoformans* | 6.4048 | 0.1768 | 3.2208 | **0.0013** | 0.3367 | 10.3738 | 0.0013 |
| *Trichosporon asahii* | 6.0809 | 0.1763 | 3.2125 | **0.0013** | 0.3466 | 10.3202 | 0.0013 |
| *Colletotrichum higginsianum* | 6.1073 | 0.1759 | 3.2043 | **0.0014** | 0.3565 | 10.2675 | 0.0014 |
| *Sporisorium reilianum* | 6.0564 | 0.1754 | 3.1957 | **0.0014** | 0.3672 | 10.2128 | 0.0014 |
| *Agaricus bisporus* | 6.1271 | 0.1751 | 3.1900 | **0.0015** | 0.3745 | 10.1758 | 0.0014 |
| *Leptosphaeria maculans* | 6.1826 | 0.1750 | 3.1882 | **0.0015** | 0.3768 | 10.1646 | 0.0014 |
| *Moesziomyces antarcticus* | 5.9040 | 0.1746 | 3.1818 | **0.0015** | 0.3852 | 10.1236 | 0.0015 |
| *Purpureocillium lilacinum* | 5.9325 | 0.1745 | 3.1786 | **0.0015** | 0.3894 | 10.1037 | 0.0015 |
| *Cutaneotrichosporon oleaginosum* | 5.9509 | 0.1738 | 3.1669 | **0.0016** | 0.4053 | 10.0293 | 0.0015 |
| *Magnaporthe oryzae* | 6.0221 | 0.1734 | 3.1599 | **0.0016** | 0.4150 | 9.9853 | 0.0016 |
| *Trametes versicolor* | 5.9613 | 0.1734 | 3.1596 | **0.0016** | 0.4156 | 9.9828 | 0.0016 |
| *Candida dubliniensis* | 6.0174 | 0.1726 | 3.1450 | **0.0017** | 0.4366 | 9.8911 | 0.0017 |
| *Lichtheimia ramosa* | 6.0287 | 0.1725 | 3.1433 | **0.0017** | 0.4392 | 9.8802 | 0.0017 |
| *Isaria fumosorosea* | 5.9921 | 0.1722 | 3.1371 | **0.0017** | 0.4484 | 9.8415 | 0.0017 |
| *Schizophyllum commune* | 6.0022 | 0.1716 | 3.1262 | **0.0018** | 0.4654 | 9.7728 | 0.0018 |
| *Tetrapisispora blattae* | 6.2190 | 0.1714 | 3.1232 | **0.0018** | 0.4700 | 9.7544 | 0.0018 |
| *Aspergillus nidulans* | 6.0250 | 0.1712 | 3.1191 | **0.0019** | 0.4767 | 9.7285 | 0.0018 |
| *Talaromyces pinophilus* | 5.9343 | 0.1692 | 3.0832 | **0.0021** | 0.5375 | 9.5063 | 0.0020 |
| *Penicillium rubens* | 5.8924 | 0.1689 | 3.0775 | **0.0021** | 0.5477 | 9.4713 | 0.0021 |
| *[Candida] intermedia* | 5.9345 | 0.1689 | 3.0771 | **0.0021** | 0.5486 | 9.4684 | 0.0021 |
| *Fusarium fujikuroi* | 5.8149 | 0.1682 | 3.0637 | **0.0022** | 0.5735 | 9.3864 | 0.0022 |
| *Yarrowia lipolytica* | 5.6934 | 0.1659 | 3.0233 | **0.0025** | 0.6553 | 9.1403 | 0.0025 |
| *Colletotrichum orchidophilum* | 5.6976 | 0.1659 | 3.0221 | **0.0026** | 0.6579 | 9.1330 | 0.0025 |
| *Saccharomycopsis* | 5.7774 | 0.1656 | 3.0169 | **0.0026** | 0.6691 | 9.1019 | 0.0026 |
| *Naumovozyma dairenensis* | 5.8868 | 0.1650 | 3.0063 | **0.0027** | 0.6929 | 9.0376 | 0.0026 |
| *Phialophora attae* | 5.4987 | 0.1640 | 2.9883 | **0.0029** | 0.7346 | 8.9302 | 0.0028 |
| *Colletotrichum graminicola* | 5.6669 | 0.1638 | 2.9841 | **0.0029** | 0.7448 | 8.9048 | 0.0028 |
| *Grosmannia clavigera* | 5.6026 | 0.1630 | 2.9706 | **0.0030** | 0.7782 | 8.8242 | 0.0030 |
| *Trametes hirsuta* | 5.7119 | 0.1626 | 2.9631 | **0.0031** | 0.7970 | 8.7801 | 0.0030 |
| *g_Fusarium;Other* | 5.7136 | 0.1626 | 2.9627 | **0.0031** | 0.7981 | 8.7776 | 0.0030 |
| *Aspergillus aculeatus* | 5.6938 | 0.1625 | 2.9607 | **0.0031** | 0.8033 | 8.7657 | 0.0031 |
| *Wickerhamomyces ciferrii* | 5.7185 | 0.1623 | 2.9567 | **0.0032** | 0.8137 | 8.7423 | 0.0031 |
| *Neurospora crassa* | 5.8027 | 0.1615 | 2.9425 | **0.0033** | 0.8516 | 8.6585 | 0.0033 |
| *Coniophora puteana* | 5.6652 | 0.1612 | 2.9363 | **0.0034** | 0.8688 | 8.6220 | 0.0033 |
| *Paraphaeosphaeria sporulosa* | 5.5712 | 0.1605 | 2.9250 | **0.0035** | 0.9008 | 8.5556 | 0.0034 |
| *Saccharomycopsis fibuligera x Saccharomycopsis cf. fibuligera* | 5.5666 | 0.1589 | 2.8960 | **0.0038** | 0.9879 | 8.3866 | 0.0038 |
| *Punctularia strigosozonata* | 5.5413 | 0.1586 | 2.8901 | **0.0039** | 1.0000 | 8.3527 | 0.0039 |
| *Aspergillus oryzae* | 5.4465 | 0.1586 | 2.8900 | **0.0039** | 1.0000 | 8.3523 | 0.0039 |
| *Schizosaccharomyces pombe* | 5.6022 | 0.1586 | 2.8892 | **0.0039** | 1.0000 | 8.3473 | 0.0039 |
| *Beauveria bassiana* | 5.4573 | 0.1585 | 2.8886 | **0.0039** | 1.0000 | 8.3441 | 0.0039 |

| | | | | | | | |
|---|---|---|---|---|---|---|---|
| *Xanthophyllomyces dendrorhous* | 5.4717 | 0.1581 | 2.8813 | **0.0040** | 1.0000 | 8.3021 | 0.0040 |
| *k_Fungi;Other;Other;Other;Other;Other;Other* | 5.4922 | 0.1580 | 2.8781 | **0.0041** | 1.0000 | 8.2833 | 0.0040 |
| *[Nectria] haematococca* | 5.4438 | 0.1576 | 2.8707 | **0.0042** | 1.0000 | 8.2407 | 0.0041 |
| *Chaetomium thermophilum* | 5.3389 | 0.1573 | 2.8664 | **0.0042** | 1.0000 | 8.2160 | 0.0042 |
| *Ascoidea rubescens* | 5.6307 | 0.1573 | 2.8658 | **0.0042** | 1.0000 | 8.2128 | 0.0042 |
| *Sporisorium scitamineum* | 5.5584 | 0.1566 | 2.8540 | **0.0044** | 1.0000 | 8.1455 | 0.0043 |
| *[Candida] glabrata* | 5.5276 | 0.1563 | 2.8472 | **0.0045** | 1.0000 | 8.1066 | 0.0044 |
| *Aspergillus terreus* | 5.3517 | 0.1561 | 2.8448 | **0.0045** | 1.0000 | 8.0928 | 0.0044 |
| *Puccinia graminis* | 5.4627 | 0.1561 | 2.8433 | **0.0045** | 1.0000 | 8.0844 | 0.0045 |
| *Penicilliopsis zonata* | 5.2980 | 0.1557 | 2.8377 | **0.0046** | 1.0000 | 8.0523 | 0.0045 |
| *Candida tropicalis* | 5.4135 | 0.1555 | 2.8327 | **0.0047** | 1.0000 | 8.0243 | 0.0046 |
| *Fusarium verticillioides* | 5.4208 | 0.1554 | 2.8322 | **0.0047** | 1.0000 | 8.0212 | 0.0046 |
| *Malassezia sympodialis* | 5.3562 | 0.1553 | 2.8300 | **0.0047** | 1.0000 | 8.0091 | 0.0047 |
| *Melampsora larici-populina* | 5.5203 | 0.1553 | 2.8287 | **0.0047** | 1.0000 | 8.0015 | 0.0047 |
| *Pestalotiopsis fici* | 5.3785 | 0.1552 | 2.8275 | **0.0048** | 1.0000 | 7.9948 | 0.0047 |
| *Millerozyma farinosa* | 5.4657 | 0.1551 | 2.8257 | **0.0048** | 1.0000 | 7.9849 | 0.0047 |
| *Phycomyces blakesleeanus* | 5.4953 | 0.1549 | 2.8227 | **0.0048** | 1.0000 | 7.9677 | 0.0048 |
| *Kluyveromyces marxianus* | 5.6946 | 0.1547 | 2.8193 | **0.0049** | 1.0000 | 7.9483 | 0.0048 |
| *g_Aspergillus;Other* | 5.3912 | 0.1539 | 2.8037 | **0.0051** | 1.0000 | 7.8607 | 0.0051 |
| *Trichoderma virens* | 5.2558 | 0.1533 | 2.7925 | **0.0053** | 1.0000 | 7.7982 | 0.0052 |
| *Chaetomium globosum* | 5.4907 | 0.1533 | 2.7922 | **0.0053** | 1.0000 | 7.7964 | 0.0052 |
| *Marssonina brunnea* | 5.4373 | 0.1529 | 2.7865 | **0.0054** | 1.0000 | 7.7646 | 0.0053 |
| *c_Sordariomycetes;Other;Other;Other;Other* | 5.2855 | 0.1527 | 2.7823 | **0.0055** | 1.0000 | 7.7412 | 0.0054 |
| *Rasamsonia emersonii* | 5.3238 | 0.1527 | 2.7818 | **0.0055** | 1.0000 | 7.7382 | 0.0054 |
| *Tilletiaria anomala* | 5.3799 | 0.1525 | 2.7779 | **0.0056** | 1.0000 | 7.7165 | 0.0055 |
| *Sugiyamaella lignohabitans* | 5.0867 | 0.1523 | 2.7748 | **0.0056** | 1.0000 | 7.6995 | 0.0055 |
| *Tetrapisispora phaffii* | 5.4835 | 0.1523 | 2.7740 | **0.0056** | 1.0000 | 7.6952 | 0.0055 |
| *Debaryomyces hansenii* | 5.3825 | 0.1520 | 2.7702 | **0.0057** | 1.0000 | 7.6737 | 0.0056 |
| *Baudoinia panamericana* | 5.1500 | 0.1520 | 2.7699 | **0.0057** | 1.0000 | 7.6722 | 0.0056 |
| *Cladophialophora immunda* | 5.1670 | 0.1519 | 2.7684 | **0.0057** | 1.0000 | 7.6642 | 0.0056 |
| *Phialocephala scopiformis* | 5.3889 | 0.1508 | 2.7473 | **0.0061** | 1.0000 | 7.5476 | 0.0060 |
| *Lobosporangium transversale* | 5.3594 | 0.1506 | 2.7447 | **0.0061** | 1.0000 | 7.5335 | 0.0061 |
| *Pochonia chlamydosporia* | 5.2951 | 0.1503 | 2.7384 | **0.0063** | 1.0000 | 7.4986 | 0.0062 |
| *Zygosaccharomyces parabailii* | 5.2309 | 0.1501 | 2.7344 | **0.0063** | 1.0000 | 7.4769 | 0.0062 |
| *Penicillium arizonense* | 5.1128 | 0.1499 | 2.7319 | **0.0064** | 1.0000 | 7.4632 | 0.0063 |
| *Kazachstania africana* | 4.9444 | 0.1497 | 2.7283 | **0.0065** | 1.0000 | 7.4438 | 0.0064 |
| *Dichomitus squalens* | 5.3177 | 0.1493 | 2.7210 | **0.0066** | 1.0000 | 7.4037 | 0.0065 |
| *Heterobasidion irregulare* | 5.3650 | 0.1490 | 2.7153 | **0.0067** | 1.0000 | 7.3728 | 0.0066 |
| *Sphaerulina musiva* | 5.2047 | 0.1489 | 2.7134 | **0.0067** | 1.0000 | 7.3626 | 0.0067 |
| *Fonsecaea erecta* | 5.0338 | 0.1489 | 2.7120 | **0.0068** | 1.0000 | 7.3552 | 0.0067 |

| | | | | | | | |
|---|---|---|---|---|---|---|---|
| *g_Leptosphaeria;Other* | 5.1379 | 0.1487 | 2.7087 | **0.0068** | 1.0000 | 7.3369 | 0.0068 |
| *Fusarium oxysporum* | 5.3274 | 0.1485 | 2.7065 | **0.0069** | 1.0000 | 7.3251 | 0.0068 |
| *Sordaria macrospora* | 5.1820 | 0.1485 | 2.7055 | **0.0069** | 1.0000 | 7.3199 | 0.0068 |
| *Talaromyces stipitatus* | 5.1720 | 0.1484 | 2.7042 | **0.0069** | 1.0000 | 7.3128 | 0.0068 |
| *Melanopsichium pennsylvanicum* | 5.2714 | 0.1481 | 2.6975 | **0.0071** | 1.0000 | 7.2766 | 0.0070 |
| *Gloeophyllum trabeum* | 5.0801 | 0.1477 | 2.6919 | **0.0072** | 1.0000 | 7.2464 | 0.0071 |
| *Naumovozyma castellii* | 5.3123 | 0.1476 | 2.6883 | **0.0073** | 1.0000 | 7.2272 | 0.0072 |
| *Exophiala xenobiotica* | 5.1169 | 0.1471 | 2.6802 | **0.0075** | 1.0000 | 7.1836 | 0.0074 |
| *Aureobasidium subglaciale* | 5.0617 | 0.1463 | 2.6651 | **0.0078** | 1.0000 | 7.1030 | 0.0077 |
| *Setosphaeria turcica* | 5.2378 | 0.1462 | 2.6646 | **0.0078** | 1.0000 | 7.0999 | 0.0077 |
| *Alternaria alternata* | 5.0826 | 0.1462 | 2.6636 | **0.0078** | 1.0000 | 7.0950 | 0.0077 |
| *Cryptococcus gattii VGI* | 5.2366 | 0.1459 | 2.6587 | **0.0079** | 1.0000 | 7.0685 | 0.0078 |
| *Cyphellophora europaea* | 5.1203 | 0.1456 | 2.6523 | **0.0081** | 1.0000 | 7.0348 | 0.0080 |
| *Phaeoacremonium minimum* | 5.0031 | 0.1455 | 2.6509 | **0.0081** | 1.0000 | 7.0274 | 0.0080 |
| *Schizosaccharomyces octosporus* | 4.9802 | 0.1454 | 2.6489 | **0.0082** | 1.0000 | 7.0164 | 0.0081 |
| *Pseudozyma hubeiensis* | 5.0632 | 0.1454 | 2.6485 | **0.0082** | 1.0000 | 7.0144 | 0.0081 |
| *g_Fonsecaea;Other* | 5.1059 | 0.1452 | 2.6459 | **0.0082** | 1.0000 | 7.0010 | 0.0081 |
| *Pseudogymnoascus verrucosus* | 4.9897 | 0.1448 | 2.6376 | **0.0085** | 1.0000 | 6.9570 | 0.0083 |
| *Trichoderma atroviride* | 5.1098 | 0.1447 | 2.6366 | **0.0085** | 1.0000 | 6.9518 | 0.0084 |
| *Pseudocercospora fijiensis* | 5.1678 | 0.1443 | 2.6299 | **0.0086** | 1.0000 | 6.9163 | 0.0085 |
| *Kalmanozyma brasiliensis* | 4.9756 | 0.1441 | 2.6260 | **0.0087** | 1.0000 | 6.8958 | 0.0086 |
| *Phanerochaete carnosa* | 5.1281 | 0.1439 | 2.6213 | **0.0089** | 1.0000 | 6.8711 | 0.0088 |
| *g_Bipolaris;Other* | 5.0573 | 0.1438 | 2.6203 | **0.0089** | 1.0000 | 6.8659 | 0.0088 |
| *Scheffersomyces stipitis* | 5.1617 | 0.1437 | 2.6175 | **0.0090** | 1.0000 | 6.8512 | 0.0089 |
| *Ustilago bromivora* | 5.0847 | 0.1436 | 2.6156 | **0.0090** | 1.0000 | 6.8413 | 0.0089 |
| *Schizosaccharomyces cryophilus* | 4.8851 | 0.1433 | 2.6113 | **0.0091** | 1.0000 | 6.8187 | 0.0090 |
| *Candida orthopsilosis* | 4.9206 | 0.1430 | 2.6052 | **0.0093** | 1.0000 | 6.7873 | 0.0092 |
| *Exophiala oligosperma* | 4.9876 | 0.1428 | 2.6022 | **0.0094** | 1.0000 | 6.7712 | 0.0093 |
| *Aspergillus glaucus* | 5.0842 | 0.1428 | 2.6010 | **0.0094** | 1.0000 | 6.7654 | 0.0093 |
| *Verticillium alfalfae* | 5.0766 | 0.1427 | 2.5994 | **0.0094** | 1.0000 | 6.7571 | 0.0093 |
| *Kwoniella pini* | 4.9580 | 0.1426 | 2.5985 | **0.0095** | 1.0000 | 6.7520 | 0.0094 |
| *Coniosporium apollinis* | 4.9917 | 0.1420 | 2.5875 | **0.0098** | 1.0000 | 6.6951 | 0.0097 |
| *Hyphopichia burtonii* | 5.0423 | 0.1415 | 2.5779 | **0.0101** | 1.0000 | 6.6457 | 0.0099 |
| *Eremothecium gossypii* | 4.6305 | 0.1412 | 2.5731 | **0.0102** | 1.0000 | 6.6210 | 0.0101 |
| *Fonsecaea multimorphosa* | 4.9322 | 0.1411 | 2.5705 | **0.0103** | 1.0000 | 6.6074 | 0.0102 |
| *g_Metarhizium;Other* | 4.7592 | 0.1410 | 2.5698 | **0.0103** | 1.0000 | 6.6041 | 0.0102 |
| *Capronia coronata* | 4.8983 | 0.1410 | 2.5695 | **0.0103** | 1.0000 | 6.6026 | 0.0102 |
| *Komagataella phaffii* | 4.9096 | 0.1409 | 2.5668 | **0.0104** | 1.0000 | 6.5887 | 0.0103 |
| *Xylona heveae* | 4.9206 | 0.1408 | 2.5650 | **0.0104** | 1.0000 | 6.5793 | 0.0103 |
| *Verruconis gallopava* | 4.9507 | 0.1405 | 2.5607 | **0.0106** | 1.0000 | 6.5572 | 0.0104 |
| *Candida parapsilosis* | 4.9990 | 0.1405 | 2.5602 | **0.0106** | 1.0000 | 6.5545 | 0.0105 |

| | | | | | | | | |
|---|---|---|---|---|---|---|---|---|
| *Zygosaccharomyces rouxii* | 5.0402 | 0.1397 | 2.5453 | **0.0110** | 1.0000 | 6.4787 | 0.0109 |
| *Kluyveromyces lactis* | 4.9863 | 0.1395 | 2.5424 | **0.0111** | 1.0000 | 6.4640 | 0.0110 |
| *Coprinopsis cinerea* | 4.8538 | 0.1394 | 2.5399 | **0.0112** | 1.0000 | 6.4509 | 0.0111 |
| *Spathaspora passalidarum* | 4.9710 | 0.1391 | 2.5348 | **0.0114** | 1.0000 | 6.4254 | 0.0113 |
| *Cladophialophora carrionii* | 4.7943 | 0.1391 | 2.5335 | **0.0114** | 1.0000 | 6.4188 | 0.0113 |
| *Eremothecium cymbalariae* | 4.7529 | 0.1390 | 2.5329 | **0.0114** | 1.0000 | 6.4157 | 0.0113 |
| *Fusarium graminearum* | 5.0099 | 0.1387 | 2.5264 | **0.0116** | 1.0000 | 6.3829 | 0.0115 |
| *Aspergillus clavatus* | 4.8648 | 0.1385 | 2.5237 | **0.0117** | 1.0000 | 6.3693 | 0.0116 |
| *Exophiala mesophila* | 5.0644 | 0.1385 | 2.5233 | **0.0117** | 1.0000 | 6.3672 | 0.0116 |
| *Stereum hirsutum* | 4.8681 | 0.1383 | 2.5194 | **0.0119** | 1.0000 | 6.3473 | 0.0118 |
| *Pichia membranifaciens* | 4.5937 | 0.1374 | 2.5025 | **0.0125** | 1.0000 | 6.2623 | 0.0123 |
| *Torulaspora delbrueckii* | 4.6723 | 0.1369 | 2.4939 | **0.0128** | 1.0000 | 6.2194 | 0.0126 |
| *Vanderwaltozyma polyspora* | 4.8333 | 0.1364 | 2.4857 | **0.0131** | 1.0000 | 6.1787 | 0.0129 |
| *Exophiala spinifera* | 4.7678 | 0.1358 | 2.4751 | **0.0134** | 1.0000 | 6.1260 | 0.0133 |
| *Eremothecium sinecaudum* | 4.6926 | 0.1357 | 2.4727 | **0.0135** | 1.0000 | 6.1142 | 0.0134 |
| *Lachancea thermotolerans* | 4.5738 | 0.1354 | 2.4669 | **0.0138** | 1.0000 | 6.0855 | 0.0136 |
| *Capronia epimyces* | 4.8305 | 0.1351 | 2.4609 | **0.0140** | 1.0000 | 6.0560 | 0.0139 |
| *Exophiala dermatitidis* | 4.7695 | 0.1351 | 2.4607 | **0.0140** | 1.0000 | 6.0549 | 0.0139 |
| *Ustilago maydis* | 4.4903 | 0.1350 | 2.4595 | **0.0140** | 1.0000 | 6.0490 | 0.0139 |
| *Talaromyces marneffei* | 4.8445 | 0.1345 | 2.4511 | **0.0144** | 1.0000 | 6.0079 | 0.0142 |
| *Laccaria bicolor* | 4.8493 | 0.1344 | 2.4483 | **0.0145** | 1.0000 | 5.9939 | 0.0144 |
| *Wickerhamomyces anomalus* | 4.8406 | 0.1339 | 2.4404 | **0.0148** | 1.0000 | 5.9554 | 0.0147 |
| *Fibroporia radiculosa* | 4.4733 | 0.1339 | 2.4404 | **0.0148** | 1.0000 | 5.9554 | 0.0147 |
| *Pneumocystis jirovecii* | 4.7702 | 0.1339 | 2.4395 | **0.0148** | 1.0000 | 5.9510 | 0.0147 |
| *Babjeviella inositovora* | 4.4533 | 0.1336 | 2.4338 | **0.0151** | 1.0000 | 5.9232 | 0.0149 |
| *Metschnikowia bicuspidata* | 4.7447 | 0.1331 | 2.4259 | **0.0154** | 1.0000 | 5.8850 | 0.0153 |
| *Cladophialophora yegresii* | 4.4756 | 0.1331 | 2.4248 | **0.0155** | 1.0000 | 5.8797 | 0.0153 |
| *Kwoniella dejecticola* | 4.6684 | 0.1330 | 2.4238 | **0.0155** | 1.0000 | 5.8748 | 0.0154 |
| *Endocarpon pusillum* | 4.5058 | 0.1324 | 2.4124 | **0.0160** | 1.0000 | 5.8197 | 0.0158 |
| *Blastomyces gilchristii* | 4.7355 | 0.1321 | 2.4073 | **0.0162** | 1.0000 | 5.7951 | 0.0161 |
| *Rhinocladiella mackenziei* | 4.7004 | 0.1319 | 2.4040 | **0.0164** | 1.0000 | 5.7790 | 0.0162 |
| *Spizellomyces punctatus* | 4.6587 | 0.1319 | 2.4030 | **0.0164** | 1.0000 | 5.7744 | 0.0163 |
| *Meyerozyma guilliermondii* | 4.3159 | 0.1318 | 2.4008 | **0.0165** | 1.0000 | 5.7637 | 0.0164 |
| *Glarea lozoyensis* | 4.7407 | 0.1317 | 2.3993 | **0.0166** | 1.0000 | 5.7566 | 0.0164 |
| *Pyrenophora tritici-repentis* | 4.6417 | 0.1309 | 2.3847 | **0.0172** | 1.0000 | 5.6868 | 0.0171 |
| *Talaromyces atroroseus* | 4.4597 | 0.1305 | 2.3781 | **0.0175** | 1.0000 | 5.6554 | 0.0174 |
| *Kazachstania naganishii* | 4.6077 | 0.1302 | 2.3726 | **0.0178** | 1.0000 | 5.6293 | 0.0177 |
| *Nannizzia gypsea* | 4.5650 | 0.1302 | 2.3720 | **0.0178** | 1.0000 | 5.6264 | 0.0177 |
| *Aspergillus bombycis* | 4.4003 | 0.1300 | 2.3688 | **0.0180** | 1.0000 | 5.6113 | 0.0178 |
| *Bipolaris maydis* | 4.6466 | 0.1299 | 2.3675 | **0.0181** | 1.0000 | 5.6051 | 0.0179 |
| *Scedosporium apiospermum* | 4.6638 | 0.1299 | 2.3661 | **0.0181** | 1.0000 | 5.5986 | 0.0180 |
| *Penicillium expansum* | 4.5344 | 0.1292 | 2.3543 | **0.0187** | 1.0000 | 5.5429 | 0.0186 |

| | | | | | | | |
|---|---|---|---|---|---|---|---|
| *g_Cladophialophora;Other* | 4.3083 | 0.1283 | 2.3374 | **0.0196** | 1.0000 | 5.4635 | 0.0194 |
| *Neurospora tetrasperma* | 4.3735 | 0.1280 | 2.3323 | **0.0198** | 1.0000 | 5.4394 | 0.0197 |
| *Cladophialophora psammophila* | 4.2597 | 0.1276 | 2.3248 | **0.0202** | 1.0000 | 5.4048 | 0.0201 |
| *Fomitiporia mediterranea* | 4.4846 | 0.1271 | 2.3159 | **0.0207** | 1.0000 | 5.3634 | 0.0206 |
| *Metarhizium acridum* | 4.4994 | 0.1267 | 2.3080 | **0.0212** | 1.0000 | 5.3268 | 0.0210 |
| *Pneumocystis carinii* | 4.4591 | 0.1261 | 2.2982 | **0.0217** | 1.0000 | 5.2816 | 0.0216 |
| *Bipolaris sorokiniana* | 4.2878 | 0.1258 | 2.2924 | **0.0220** | 1.0000 | 5.2551 | 0.0219 |
| *Exophiala aquamarina* | 4.6599 | 0.1256 | 2.2882 | **0.0223** | 1.0000 | 5.2358 | 0.0221 |
| *Pichia kudriavzevii* | 4.2684 | 0.1255 | 2.2857 | **0.0224** | 1.0000 | 5.2246 | 0.0223 |
| *Batrachochytrium dendrobatidis* | 4.4182 | 0.1254 | 2.2852 | **0.0225** | 1.0000 | 5.2221 | 0.0223 |
| *Pneumocystis murina* | 4.4193 | 0.1252 | 2.2814 | **0.0227** | 1.0000 | 5.2046 | 0.0225 |
| *g_Verticillium;Other* | 4.3882 | 0.1252 | 2.2811 | **0.0227** | 1.0000 | 5.2034 | 0.0225 |
| *Debaryomyces fabryi* | 4.1585 | 0.1249 | 2.2756 | **0.0230** | 1.0000 | 5.1785 | 0.0229 |
| *Wallemia mellicola* | 4.3882 | 0.1247 | 2.2718 | **0.0233** | 1.0000 | 5.1610 | 0.0231 |
| *Saccharomycetaceae sp. Ashbya aceri* | 4.4003 | 0.1247 | 2.2715 | **0.0233** | 1.0000 | 5.1596 | 0.0231 |
| *Malassezia pachydermatis* | 4.0470 | 0.1241 | 2.2618 | **0.0239** | 1.0000 | 5.1156 | 0.0237 |
| *Histoplasma capsulatum* | 4.1664 | 0.1240 | 2.2586 | **0.0241** | 1.0000 | 5.1015 | 0.0239 |
| *Cryptococcus amylolentus* | 4.3283 | 0.1233 | 2.2473 | **0.0248** | 1.0000 | 5.0502 | 0.0246 |
| *Aspergillus fischeri* | 4.3236 | 0.1233 | 2.2473 | **0.0248** | 1.0000 | 5.0502 | 0.0246 |
| *Aureobasidium namibiae* | 4.2787 | 0.1233 | 2.2461 | **0.0249** | 1.0000 | 5.0450 | 0.0247 |
| *Uncinocarpus reesii* | 4.4980 | 0.1233 | 2.2459 | **0.0249** | 1.0000 | 5.0441 | 0.0247 |
| *Ogataea polymorpha* | 4.1608 | 0.1229 | 2.2390 | **0.0253** | 1.0000 | 5.0129 | 0.0252 |
| *Kuraishia capsulata* | 4.2856 | 0.1228 | 2.2376 | **0.0254** | 1.0000 | 5.0069 | 0.0252 |
| *Lodderomyces elongisporus* | 4.3747 | 0.1228 | 2.2375 | **0.0254** | 1.0000 | 5.0062 | 0.0253 |
| *Tremella mesenterica* | 4.1837 | 0.1224 | 2.2295 | **0.0260** | 1.0000 | 4.9708 | 0.0258 |
| *Fusarium pseudograminearum* | 4.0821 | 0.1223 | 2.2283 | **0.0260** | 1.0000 | 4.9651 | 0.0259 |
| *Serpula lacrymans* | 4.5978 | 0.1222 | 2.2258 | **0.0262** | 1.0000 | 4.9544 | 0.0260 |
| *Cladophialophora bantiana* | 4.2013 | 0.1217 | 2.2174 | **0.0268** | 1.0000 | 4.9168 | 0.0266 |
| *Suhomyces tanzawaensis* | 4.3779 | 0.1215 | 2.2138 | **0.0270** | 1.0000 | 4.9009 | 0.0268 |
| *Arthrobotrys oligospora* | 4.4221 | 0.1214 | 2.2115 | **0.0272** | 1.0000 | 4.8905 | 0.0270 |
| *Zygosaccharomyces bailii* | 4.1603 | 0.1205 | 2.1956 | **0.0283** | 1.0000 | 4.8205 | 0.0281 |
| *o_Saccharomycetales;Other; Other;Other* | 4.2578 | 0.1205 | 2.1953 | **0.0283** | 1.0000 | 4.8194 | 0.0281 |
| *Cyberlindnera fabianii* | 4.2380 | 0.1203 | 2.1918 | **0.0286** | 1.0000 | 4.8038 | 0.0284 |
| *Aspergillus fumigatus* | 4.1414 | 0.1183 | 2.1545 | **0.0314** | 1.0000 | 4.6417 | 0.0312 |
| *Saitoella complicata* | 3.9335 | 0.1181 | 2.1526 | **0.0315** | 1.0000 | 4.6337 | 0.0314 |
| *Mixia osmundae* | 4.2000 | 0.1180 | 2.1502 | **0.0317** | 1.0000 | 4.6236 | 0.0315 |
| *Saccharomycopsis fibuligera* | 4.0400 | 0.1176 | 2.1426 | **0.0323** | 1.0000 | 4.5909 | 0.0321 |
| *[Candida] tenuis* | 4.1375 | 0.1171 | 2.1336 | **0.0331** | 1.0000 | 4.5524 | 0.0329 |
| *Fusarium culmorum* | 4.3147 | 0.1164 | 2.1213 | **0.0341** | 1.0000 | 4.4998 | 0.0339 |
| *Kwoniella mangrovensis* | 4.3752 | 0.1163 | 2.1190 | **0.0343** | 1.0000 | 4.4902 | 0.0341 |

| | | | | | | | |
|---|---|---|---|---|---|---|---|
| *Schizosaccharomyces japonicus* | 4.3314 | 0.1161 | 2.1158 | **0.0345** | 1.0000 | 4.4768 | 0.0344 |
| *Fonsecaea nubica* | 4.7968 | 0.1154 | 2.1025 | **0.0357** | 1.0000 | 4.4203 | 0.0355 |
| *Microsporum canis* | 4.0577 | 0.1141 | 2.0796 | **0.0378** | 1.0000 | 4.3249 | 0.0376 |
| *Trichoderma gamsii* | 3.7308 | 0.1131 | 2.0601 | **0.0396** | 1.0000 | 4.2440 | 0.0394 |
| *Kwoniella bestiolae* | 4.1052 | 0.1131 | 2.0600 | **0.0396** | 1.0000 | 4.2436 | 0.0394 |
| *g_Coccidioides;Other* | 3.9752 | 0.1130 | 2.0583 | **0.0398** | 1.0000 | 4.2366 | 0.0396 |
| *Kockovaella imperatae* | 4.0662 | 0.1125 | 2.0491 | **0.0407** | 1.0000 | 4.1989 | 0.0405 |
| *Tuber melanosporum* | 4.0785 | 0.1105 | 2.0133 | **0.0443** | 1.0000 | 4.0532 | 0.0441 |
| *Penicillium digitatum* | 3.8134 | 0.1103 | 2.0104 | **0.0446** | 1.0000 | 4.0417 | 0.0444 |
| *Saccharomyces eubayanus* | 3.8433 | 0.1096 | 1.9975 | **0.0460** | 1.0000 | 3.9899 | 0.0458 |
| *Aspergillus nomius* | 4.1684 | 0.1094 | 1.9936 | **0.0464** | 1.0000 | 3.9745 | 0.0462 |
| *Wallemia ichthyophaga* | 3.7740 | 0.1094 | 1.9924 | **0.0465** | 1.0000 | 3.9696 | 0.0463 |
| *g_Trichophyton;Other* | 3.8443 | 0.1089 | 1.9845 | **0.0474** | 1.0000 | 3.9383 | 0.0472 |
| *Tsuchiyaea wingfieldii* | 3.7678 | 0.1087 | 1.9800 | **0.0479** | 1.0000 | 3.9206 | 0.0477 |
| *Bipolaris oryzae* | 3.7962 | 0.1083 | 1.9736 | **0.0486** | 1.0000 | 3.8952 | 0.0484 |
| *Phakopsora pachyrhizi* | 3.9408 | 0.1077 | 1.9620 | 0.0500 | 1.0000 | 3.8494 | 0.0498 |
| *Cyberlindnera jadinii* | 3.9776 | 0.1076 | 1.9609 | 0.0501 | 1.0000 | 3.8450 | 0.0499 |
| *Paracoccidioides brasiliensis* | 3.6869 | 0.1071 | 1.9506 | 0.0513 | 1.0000 | 3.8050 | 0.0511 |
| *g_Neurospora;Other* | 3.8177 | 0.1062 | 1.9357 | 0.0531 | 1.0000 | 3.7471 | 0.0529 |
| *Komagataella pastoris* | 4.0002 | 0.1062 | 1.9340 | 0.0533 | 1.0000 | 3.7405 | 0.0531 |
| *Metarhizium brunneum* | 3.5680 | 0.1048 | 1.9103 | 0.0563 | 1.0000 | 3.6491 | 0.0561 |
| *Lachancea lanzarotensis* | 3.7180 | 0.1047 | 1.9080 | 0.0566 | 1.0000 | 3.6404 | 0.0564 |
| *g_Zygosaccharomyces;Other* | 3.4649 | 0.1044 | 1.9017 | 0.0574 | 1.0000 | 3.6164 | 0.0572 |

LFC = log fold change. *limma* software R program. Log2 fold change calculated for each time point. Weeks 0, 3, 13, 26, 39, 52 were examined across six time points. Fungi abundance filtered at (>0.1%) for analysis. Taxonomic annotations: p = phylum; o = order; c = class; f = family; g = genus.

Differential relative abundance analyses over time for archaea species indicated significant increased shifts for *Methanobrevibacter smithii* (Bonferroni: *P*=0.0000), *Methanosarcina barkeri* (Bonferroni: *P*=0.01290); decreased from baseline, but increased movement across time in *Methanococcus maripaludis* (Bonferroni: *P*=0.03280) (**Table S7, Figure S7D**). Of note, the striking differences in methanogen-producing bacteria are very apparent, when compared to the observational household spouse control (**Figure S6D**). It's been known that MS patients have increased relative abundances of methanogen bacteria in their gut microbial environment.(33)

**Supplementary Table 7. At** the taxonomic level of species, *limma* powers differential abundance analyses across time for archaea in the RRMS FMT subject.

| Archaea Species | Average across time points | LFC | B-Stats | *P*-value | Bonferroni *P*-value | F | F *P*-value |
|---|---|---|---|---|---|---|---|
| *Methanobrevibacter smithii* | 11.1871 | 0.3207 | 9.4117 | **0.0000** | **0.0000** | 88.5804 | 0.0000 |
| *Methanosarcina barkeri* | 4.7383 | 0.1328 | 3.8959 | **0.0001** | **0.0129** | 15.1777 | 0.0001 |
| *Methanococcus maripaludis* | 4.4422 | 0.1246 | 3.6567 | **0.0003** | **0.0328** | 13.3715 | 0.0003 |
| *Methanosarcina mazei* | 4.1890 | 0.1154 | 3.3874 | **0.0008** | 0.0888 | 11.4744 | 0.0007 |
| *g_Methanosarcina;Other* | 4.2746 | 0.1154 | 3.3858 | **0.0008** | 0.0893 | 11.4638 | 0.0007 |
| *Haloferax mediterranei* | 3.8115 | 0.1138 | 3.3398 | **0.0009** | 0.1052 | 11.1542 | 0.0008 |
| *Methanobrevibacter millerae* | 4.2925 | 0.1059 | 3.1084 | **0.0020** | 0.2327 | 9.6621 | 0.0019 |
| *Methanocella paludicola* | 3.3663 | 0.0960 | 2.8176 | **0.0050** | 0.5900 | 7.9391 | 0.0048 |
| *c_Halobacteria;Other; Other;Other;Other* | 3.3911 | 0.0959 | 2.8138 | **0.0051** | 0.5971 | 7.9172 | 0.0049 |
| *Methanobrevibacter sp. YE315* | 3.9008 | 0.0952 | 2.7937 | **0.0054** | 0.6348 | 7.8050 | 0.0052 |
| *Methanobrevibacter olleyae* | 3.7104 | 0.0946 | 2.7771 | **0.0057** | 0.6677 | 7.7124 | 0.0055 |
| *Halorubrum lacusprofundi* | 2.9764 | 0.0944 | 2.7694 | **0.0058** | 0.6836 | 7.6693 | 0.0056 |
| *Natrinema pellirubrum* | 3.2640 | 0.0937 | 2.7503 | **0.0061** | 0.7241 | 7.5642 | 0.0060 |
| *Haloterrigena turkmenica* | 3.6109 | 0.0933 | 2.7388 | **0.0064** | 0.7496 | 7.5011 | 0.0062 |
| *Haloterrigena daqingensis* | 2.8400 | 0.0927 | 2.7213 | **0.0067** | 0.7899 | 7.4057 | 0.0065 |
| *Halobacterium sp. DL1* | 2.9115 | 0.0917 | 2.6915 | **0.0073** | 0.8632 | 7.2441 | 0.0071 |
| *Methanocorpusculum labreanum* | 3.3073 | 0.0915 | 2.6847 | **0.0075** | 0.8806 | 7.2078 | 0.0073 |
| *Halorubrum trapanicum* | 3.1177 | 0.0907 | 2.6629 | **0.0080** | 0.9390 | 7.0913 | 0.0077 |
| *Halopiger xanaduensis* | 3.1474 | 0.0888 | 2.6073 | **0.0094** | 1.0000 | 6.7978 | 0.0091 |
| *g_Thermococcus;Other* | 2.9598 | 0.0886 | 2.5988 | **0.0096** | 1.0000 | 6.7539 | 0.0094 |
| *Halobacterium hubeiense* | 2.9912 | 0.0885 | 2.5972 | **0.0096** | 1.0000 | 6.7454 | 0.0094 |
| *methanogenic archaeon ISO4-H5* | 3.1537 | 0.0884 | 2.5944 | **0.0097** | 1.0000 | 6.7308 | 0.0095 |
| *Haloferax volcanii* | 3.0295 | 0.0872 | 2.5599 | **0.0107** | 1.0000 | 6.5532 | 0.0105 |
| *Methanobrevibacter sp. AbM4* | 3.1801 | 0.0867 | 2.5443 | **0.0112** | 1.0000 | 6.4736 | 0.0109 |
| *Halorhabdus utahensis* | 2.8681 | 0.0867 | 2.5438 | **0.0112** | 1.0000 | 6.4708 | 0.0110 |
| *Methanoregula formicica* | 2.4919 | 0.0854 | 2.5073 | **0.0124** | 1.0000 | 6.2864 | 0.0122 |
| *Methanocella arvoryzae* | 2.9739 | 0.0851 | 2.4963 | **0.0128** | 1.0000 | 6.2317 | 0.0125 |
| *Natrinema sp. J7-2* | 2.7419 | 0.0850 | 2.4935 | **0.0129** | 1.0000 | 6.2176 | 0.0126 |
| *Haloferax gibbonsii* | 2.6912 | 0.0841 | 2.4679 | **0.0139** | 1.0000 | 6.0903 | 0.0136 |
| *Halorientalis sp. IM1011* | 2.5089 | 0.0829 | 2.4339 | **0.0152** | 1.0000 | 5.9238 | 0.0149 |

| | | | | | | | |
|---|---|---|---|---|---|---|---|
| *Methanobacterium formicicum* | 3.1703 | 0.0828 | 2.4290 | **0.0154** | 1.0000 | 5.8998 | 0.0151 |
| *Halobiforma lacisalsi* | 3.0393 | 0.0822 | 2.4125 | **0.0161** | 1.0000 | 5.8203 | 0.0158 |
| *Sulfolobus islandicus* | 2.7844 | 0.0821 | 2.4099 | **0.0163** | 1.0000 | 5.8077 | 0.0160 |
| *Methanosarcina acetivorans* | 2.8306 | 0.0802 | 2.3531 | **0.0189** | 1.0000 | 5.5372 | 0.0186 |
| *Methanobrevibacter ruminantium* | 3.4453 | 0.0802 | 2.3524 | **0.0190** | 1.0000 | 5.5337 | 0.0187 |
| *Thermoplasmatales archaeon BRNA1* | 3.0789 | 0.0788 | 2.3126 | **0.0211** | 1.0000 | 5.3482 | 0.0207 |
| *Halovivax ruber* | 2.5343 | 0.0769 | 2.2577 | **0.0243** | 1.0000 | 5.0971 | 0.0240 |
| *Methanoculleus marisnigri* | 2.5850 | 0.0769 | 2.2571 | **0.0244** | 1.0000 | 5.0944 | 0.0240 |
| *Natrialbaceae archaeon JW/NM-HA 15* | 2.5869 | 0.0766 | 2.2483 | **0.0249** | 1.0000 | 5.0550 | 0.0246 |
| *Cenarchaeum symbiosum* | 2.2165 | 0.0760 | 2.2289 | **0.0262** | 1.0000 | 4.9681 | 0.0258 |
| *Methanolacinia petrolearia* | 2.8324 | 0.0758 | 2.2258 | **0.0264** | 1.0000 | 4.9543 | 0.0260 |
| *Methanosarcina siciliae* | 3.0253 | 0.0754 | 2.2123 | **0.0273** | 1.0000 | 4.8942 | 0.0269 |
| *Methanobacterium paludis* | 2.7381 | 0.0750 | 2.2024 | **0.0280** | 1.0000 | 4.8505 | 0.0276 |
| *Methanosarcina sp. MTP4* | 2.6035 | 0.0748 | 2.1963 | **0.0285** | 1.0000 | 4.8237 | 0.0281 |
| *Candidatus Methanomethylophilus alvus* | 2.2604 | 0.0738 | 2.1667 | **0.0307** | 1.0000 | 4.6945 | 0.0303 |
| *Methanotorris igneus* | 2.2604 | 0.0736 | 2.1597 | **0.0312** | 1.0000 | 4.6645 | 0.0308 |
| *Natronobacterium gregoryi* | 2.4153 | 0.0732 | 2.1472 | **0.0322** | 1.0000 | 4.6106 | 0.0318 |
| *Halogeometricum borinquense* | 2.7426 | 0.0726 | 2.1305 | **0.0335** | 1.0000 | 4.5390 | 0.0331 |
| *Methanococcus voltae* | 2.2331 | 0.0722 | 2.1179 | **0.0346** | 1.0000 | 4.4853 | 0.0342 |
| *Methanosphaera stadtmanae* | 2.7165 | 0.0717 | 2.1033 | **0.0359** | 1.0000 | 4.4239 | 0.0354 |
| *Natronococcus occultus* | 2.4679 | 0.0715 | 2.0994 | **0.0362** | 1.0000 | 4.4074 | 0.0358 |
| *g_Haloarcula;Other* | 2.4436 | 0.0713 | 2.0929 | **0.0368** | 1.0000 | 4.3803 | 0.0364 |
| *g_Methanoculleus;Other* | 3.4221 | 0.0694 | 2.0352 | **0.0423** | 1.0000 | 4.1421 | 0.0418 |
| *Acidianus manzaensis* | 2.1356 | 0.0692 | 2.0307 | **0.0427** | 1.0000 | 4.1236 | 0.0423 |
| *Halomicrobium mukohataei* | 2.9624 | 0.0689 | 2.0226 | **0.0436** | 1.0000 | 4.0911 | 0.0431 |
| *Methanohalobium evestigatum* | 2.6340 | 0.0673 | 1.9758 | **0.0486** | 1.0000 | 3.9038 | 0.0482 |
| *Halopenitus persicus* | 2.5869 | 0.0659 | 1.9345 | 0.0535 | 1.0000 | 3.7422 | 0.0531 |
| *halophilic archaeon True-ADL* | 2.0499 | 0.0657 | 1.9280 | 0.0543 | 1.0000 | 3.7170 | 0.0539 |
| *Methanococcoides methylutens* | 2.6869 | 0.0657 | 1.9266 | 0.0545 | 1.0000 | 3.7118 | 0.0540 |
| *Thermococcus barophilus* | 2.3900 | 0.0654 | 1.9198 | 0.0554 | 1.0000 | 3.6856 | 0.0549 |

| | | | | | | | |
|---|---|---|---|---|---|---|---|
| *Salinarchaeum sp. Harcht-Bsk1* | 2.6205 | 0.0645 | 1.8929 | 0.0589 | 1.0000 | 3.5832 | 0.0584 |
| LFC = log fold change. *limma* software R program. Log2 fold change calculated for each time point. Weeks 0, 3, 13, 26, 39, 52 were examined across six time points. Archaea abundance filtered at (>0.1%) for analysis. Taxonomic level classifications: p = phylum, family, g = genus. | | | | | | | |

Finally, the differential relative abundance over time for functional genomic pathways in the RRMS FMT subject was explored. Five non-targeted functional gene pathways had significant increased shifts across the continuous variable of time (**Table S8)**. Functional genomic pathways trending towards significance across time indicated higher relative abundances of Biosynthesis of amino acids (ko01230: Bonferroni: *P*=0.0562) and Methane metabolism (ko00680: Bonferroni: *P*=0.0643).

**Supplementary Table 8.** *Limma* powers differential relative abundance of functional gene pathway analyses across time in the RRMS FMT subject.

| Functional Gene Pathways | Average across time points | LFC | B-Stats | *P*-value | Bonferroni *P*-value | F | F *P*-value |
|---|---|---|---|---|---|---|---|
| ko01100 Metabolic pathways | 18.3288 | 0.5252 | 4.5586 | **0.0000** | **0.0020** | 20.7813 | 0.0000 |
| ko01120 Microbial metabolism in diverse environments | 16.9261 | 0.4855 | 4.2145 | **0.0000** | **0.0098** | 17.7622 | 0.0000 |
| ko01110 Biosynthesis of secondary metabolites | 16.8592 | 0.4835 | 4.1971 | **0.0000** | **0.0105** | 17.6159 | 0.0000 |
| ko01130 Biosynthesis of antibiotics | 16.2102 | 0.4651 | 4.0372 | **0.0001** | **0.0209** | 16.2987 | 0.0001 |
| ko01200 Carbon metabolism | 15.8651 | 0.4552 | 3.9508 | **0.0001** | **0.0301** | 15.6090 | 0.0001 |
| ko01230 Biosynthesis of amino acids | 15.2406 | 0.4375 | 3.7972 | **0.0002** | 0.0562 | 14.4186 | 0.0001 |
| ko00680 Methane metabolism | 15.1014 | 0.4336 | 3.7634 | **0.0002** | 0.0643 | 14.1634 | 0.0002 |
| ko00260 Glycine serine and threonine metabolism | 14.7990 | 0.4258 | 3.6958 | **0.0002** | 0.0839 | 13.6590 | 0.0002 |
| ko00190 Oxidative phosphorylation | 14.6544 | 0.4209 | 3.6536 | **0.0003** | 0.0989 | 13.3489 | 0.0003 |
| ko00280 Valine leucine and isoleucine degradation | 14.4121 | 0.4141 | 3.5945 | **0.0003** | 0.1241 | 12.9204 | 0.0003 |
| ko00620 Pyruvate metabolism | 14.3951 | 0.4130 | 3.5849 | **0.0003** | 0.1287 | 12.8515 | 0.0003 |
| ko01220 Degradation of aromatic compounds | 14.3297 | 0.4123 | 3.5785 | **0.0004** | 0.1318 | 12.8058 | 0.0003 |
| ko04142 Lysosome | 14.2125 | 0.4088 | 3.5485 | **0.0004** | 0.1477 | 12.5921 | 0.0004 |
| ko00230 Purine metabolism | 14.2484 | 0.4085 | 3.5462 | **0.0004** | 0.1490 | 12.5757 | 0.0004 |
| ko00630 Glyoxylate and dicarboxylate metabolism | 14.2038 | 0.4077 | 3.5390 | **0.0004** | 0.1531 | 12.5246 | 0.0004 |
| ko00640 Propanoate metabolism | 14.0385 | 0.4033 | 3.5011 | **0.0005** | 0.1764 | 12.2577 | 0.0005 |
| ko00330 Arginine and proline metabolism | 13.9012 | 0.4002 | 3.4738 | **0.0005** | 0.1952 | 12.0676 | 0.0005 |
| ko00010 Glycolysis Gluconeogenesis | 13.8771 | 0.3995 | 3.4673 | **0.0005** | 0.2000 | 12.0224 | 0.0005 |
| ko00531 Glycosaminoglycan degradation | 13.8492 | 0.3991 | 3.4644 | **0.0005** | 0.2021 | 12.0022 | 0.0005 |
| ko00350 Tyrosine metabolism | 13.8503 | 0.3990 | 3.4638 | **0.0005** | 0.2026 | 11.9977 | 0.0005 |

| | | | | | | | |
|---|---|---|---|---|---|---|---|
| ko00020 Citrate cycle TCA cycle | 13.8580 | 0.3980 | 3.4549 | **0.0006** | 0.2094 | 11.9361 | 0.0006 |
| ko00500 Starch and sucrose metabolism | 13.8340 | 0.3980 | 3.4543 | **0.0006** | 0.2098 | 11.9323 | 0.0006 |
| ko00720 Carbon fixation pathways in prokaryotes | 13.8441 | 0.3979 | 3.4542 | **0.0006** | 0.2099 | 11.9314 | 0.0006 |
| ko00564 Glycerophospholipid metabolism | 13.7863 | 0.3965 | 3.4415 | **0.0006** | 0.2199 | 11.8441 | 0.0006 |
| ko00910 Nitrogen metabolism | 13.7667 | 0.3947 | 3.4261 | **0.0006** | 0.2327 | 11.7381 | 0.0006 |
| ko00380 Tryptophan metabolism | 13.5936 | 0.3924 | 3.4063 | **0.0007** | 0.2502 | 11.6028 | 0.0007 |
| ko05010 Alzheimer disease | 13.6225 | 0.3914 | 3.3978 | **0.0007** | 0.2580 | 11.5451 | 0.0007 |
| ko01212 Fatty acid metabolism | 13.5394 | 0.3892 | 3.3781 | **0.0007** | 0.2772 | 11.4113 | 0.0007 |
| ko00650 Butanoate metabolism | 13.5463 | 0.3889 | 3.3762 | **0.0008** | 0.2791 | 11.3985 | 0.0007 |
| ko00040 Pentose and glucuronate interconversions | 13.4624 | 0.3879 | 3.3672 | **0.0008** | 0.2882 | 11.3380 | 0.0008 |
| ko00240 Pyrimidine metabolism | 13.5055 | 0.3877 | 3.3655 | **0.0008** | 0.2900 | 11.3267 | 0.0008 |
| ko00362 Benzoate degradation | 13.4421 | 0.3871 | 3.3602 | **0.0008** | 0.2956 | 11.2907 | 0.0008 |
| ko00520 Amino sugar and nucleotide sugar metabolism | 13.4185 | 0.3863 | 3.3532 | **0.0008** | 0.3031 | 11.2440 | 0.0008 |
| ko04714 Thermogenesis | 13.4289 | 0.3863 | 3.3528 | **0.0008** | 0.3035 | 11.2414 | 0.0008 |
| ko05016 Huntington disease | 13.4117 | 0.3849 | 3.3414 | **0.0009** | 0.3162 | 11.1651 | 0.0008 |
| ko05012 Parkinson disease | 13.3707 | 0.3841 | 3.3345 | **0.0009** | 0.3241 | 11.1190 | 0.0009 |
| ko04310 Wnt signaling pathway | 13.3372 | 0.3837 | 3.3306 | **0.0009** | 0.3287 | 11.0928 | 0.0009 |
| ko00071 Fatty acid degradation | 13.2722 | 0.3825 | 3.3200 | **0.0009** | 0.3414 | 11.0222 | 0.0009 |
| ko04932 Nonalcoholic fatty liver disease NAFLD | 13.2950 | 0.3823 | 3.3181 | **0.0009** | 0.3437 | 11.0097 | 0.0009 |
| ko01210 2Oxocarboxylic acid metabolism | 13.2665 | 0.3819 | 3.3147 | **0.0009** | 0.3478 | 10.9875 | 0.0009 |
| ko00270 Cysteine and methionine metabolism | 13.2698 | 0.3818 | 3.3142 | **0.0009** | 0.3485 | 10.9839 | 0.0009 |
| ko00561 Glycerolipid metabolism | 13.2169 | 0.3799 | 3.2979 | **0.0010** | 0.3692 | 10.8763 | 0.0010 |
| ko04934 Cushing syndrome | 13.2335 | 0.3799 | 3.2974 | **0.0010** | 0.3698 | 10.8730 | 0.0010 |
| ko00410 beta Alanine metabolism | 13.1874 | 0.3797 | 3.2958 | **0.0010** | 0.3720 | 10.8621 | 0.0010 |
| ko02020 Two component system | 13.2332 | 0.3796 | 3.2950 | **0.0010** | 0.3730 | 10.8572 | 0.0010 |
| ko00340 Histidine metabolism | 13.1486 | 0.3794 | 3.2930 | **0.0010** | 0.3756 | 10.8442 | 0.0010 |
| ko00051 Fructose and mannose metabolism | 13.1568 | 0.3790 | 3.2895 | **0.0010** | 0.3804 | 10.8206 | 0.0010 |
| ko00250 Alanine aspartate and glutamate metabolism | 13.2275 | 0.3787 | 3.2869 | **0.0010** | 0.3838 | 10.8039 | 0.0010 |
| ko00300 Lysine biosynthesis | 13.0577 | 0.3763 | 3.2660 | **0.0011** | 0.4131 | 10.6669 | 0.0011 |
| ko00140 Steroid hormone biosynthesis | 13.0791 | 0.3759 | 3.2632 | **0.0011** | 0.4172 | 10.6487 | 0.0011 |
| ko00360 Phenylalanine metabolism | 13.0507 | 0.3757 | 3.2609 | **0.0011** | 0.4205 | 10.6338 | 0.0011 |
| ko00400 Phenylalanine tyrosine and tryptophan biosynthesis | 13.0795 | 0.3753 | 3.2581 | **0.0011** | 0.4248 | 10.6149 | 0.0011 |
| ko05200 Pathways in cancer | 13.0325 | 0.3733 | 3.2406 | **0.0012** | 0.4516 | 10.5014 | 0.0012 |
| ko00624 Polycyclic aromatic hydrocarbon degradation | 12.9528 | 0.3730 | 3.2377 | **0.0012** | 0.4562 | 10.4826 | 0.0012 |
| ko00310 Lysine degradation | 12.9658 | 0.3726 | 3.2342 | **0.0012** | 0.4617 | 10.4600 | 0.0012 |

| | | | | | | | |
|---|---|---|---|---|---|---|---|
| ko00590 Arachidonic acid metabolism | 12.9072 | 0.3723 | 3.2315 | **0.0013** | 0.4661 | 10.4426 | 0.0012 |
| ko00710 Carbon fixation in photosynthetic organisms | 12.9007 | 0.3711 | 3.2215 | **0.0013** | 0.4826 | 10.3781 | 0.0013 |
| ko04146 Peroxisome | 12.8966 | 0.3709 | 3.2197 | **0.0013** | 0.4855 | 10.3667 | 0.0013 |
| ko04916 Melanogenesis | 12.8781 | 0.3702 | 3.2131 | **0.0013** | 0.4968 | 10.3240 | 0.0013 |
| ko04150 mTOR signaling pathway | 12.7503 | 0.3672 | 3.1878 | **0.0015** | 0.5421 | 10.1622 | 0.0014 |
| ko05166 Human Tcell leukemia virus 1 infection | 12.7437 | 0.3664 | 3.1800 | **0.0015** | 0.5569 | 10.1125 | 0.0015 |
| ko00053 Ascorbate and aldarate metabolism | 12.6964 | 0.3657 | 3.1740 | **0.0015** | 0.5685 | 10.0742 | 0.0015 |
| ko03010 Ribosome | 12.7437 | 0.3655 | 3.1726 | **0.0015** | 0.5712 | 10.0654 | 0.0015 |
| ko00052 Galactose metabolism | 12.7102 | 0.3655 | 3.1723 | **0.0015** | 0.5718 | 10.0635 | 0.0015 |
| ko05165 Human papillomavirus infection | 12.6749 | 0.3647 | 3.1656 | **0.0016** | 0.5850 | 10.0212 | 0.0015 |
| ko00030 Pentose phosphate pathway | 12.6257 | 0.3639 | 3.1589 | **0.0016** | 0.5986 | 9.9787 | 0.0016 |
| ko04260 Cardiac muscle contraction | 12.6803 | 0.3639 | 3.1584 | **0.0016** | 0.5996 | 9.9756 | 0.0016 |
| ko04726 Serotonergic synapse | 12.6183 | 0.3638 | 3.1576 | **0.0016** | 0.6012 | 9.9706 | 0.0016 |
| ko00511 Other glycan degradation | 12.5491 | 0.3623 | 3.1449 | **0.0017** | 0.6278 | 9.8903 | 0.0017 |
| ko00980 Metabolism of xenobiotics by cytochrome P450 | 12.5194 | 0.3615 | 3.1378 | **0.0017** | 0.6432 | 9.8458 | 0.0017 |
| ko00900 Terpenoid backbone biosynthesis | 12.5716 | 0.3603 | 3.1277 | **0.0018** | 0.6655 | 9.7825 | 0.0018 |
| ko00760 Nicotinate and nicotinamide metabolism | 12.5197 | 0.3599 | 3.1243 | **0.0018** | 0.6732 | 9.7612 | 0.0018 |
| ko01523 Antifolate resistance | 12.4568 | 0.3598 | 3.1229 | **0.0018** | 0.6765 | 9.7522 | 0.0018 |
| ko00061 Fatty acid biosynthesis | 12.4162 | 0.3584 | 3.1106 | **0.0019** | 0.7050 | 9.6761 | 0.0019 |
| ko00600 Sphingolipid metabolism | 12.4672 | 0.3582 | 3.1093 | **0.0019** | 0.7082 | 9.6676 | 0.0019 |
| ko05204 Chemical carcinogenesis | 12.3677 | 0.3575 | 3.1032 | **0.0019** | 0.7229 | 9.6297 | 0.0019 |
| ko00860 Porphyrin and chlorophyll metabolism | 12.4700 | 0.3573 | 3.1014 | **0.0020** | 0.7273 | 9.6186 | 0.0019 |
| ko04750 Inflammatory mediator regulation of TRP channels | 12.3946 | 0.3573 | 3.1013 | **0.0020** | 0.7275 | 9.6181 | 0.0019 |
| ko00626 Naphthalene degradation | 12.4046 | 0.3569 | 3.0982 | **0.0020** | 0.7351 | 9.5989 | 0.0019 |
| ko04925 Aldosterone synthesis and secretion | 12.4406 | 0.3567 | 3.0960 | **0.0020** | 0.7405 | 9.5853 | 0.0020 |
| ko05205 Proteoglycans in cancer | 12.3998 | 0.3565 | 3.0949 | **0.0020** | 0.7434 | 9.5782 | 0.0020 |
| ko05225 Hepatocellular carcinoma | 12.4200 | 0.3564 | 3.0940 | **0.0020** | 0.7455 | 9.5728 | 0.0020 |
| ko00950 Isoquinoline alkaloid biosynthesis | 12.3156 | 0.3557 | 3.0879 | **0.0020** | 0.7610 | 9.5349 | 0.0020 |
| ko00627 Aminobenzoate degradation | 12.2323 | 0.3547 | 3.0788 | **0.0021** | 0.7846 | 9.4787 | 0.0021 |
| ko05224 Breast cancer | 12.3385 | 0.3544 | 3.0761 | **0.0021** | 0.7917 | 9.4621 | 0.0021 |
| ko03320 PPAR signaling pathway | 12.2926 | 0.3544 | 3.0760 | **0.0021** | 0.7919 | 9.4616 | 0.0021 |
| ko00220 Arginine biosynthesis | 12.3513 | 0.3541 | 3.0738 | **0.0021** | 0.7976 | 9.4483 | 0.0021 |
| ko05226 Gastric cancer | 12.3245 | 0.3541 | 3.0733 | **0.0021** | 0.7990 | 9.4450 | 0.0021 |
| ko00621 Dioxin degradation | 12.3062 | 0.3535 | 3.0681 | **0.0022** | 0.8131 | 9.4130 | 0.0022 |
| ko00670 One carbon pool by folate | 12.2493 | 0.3534 | 3.0677 | **0.0022** | 0.8140 | 9.4108 | 0.0022 |

| | | | | | | | |
|---|---|---|---|---|---|---|---|
| ko00790 Folate biosynthesis | 12.3251 | 0.3533 | 3.0669 | **0.0022** | 0.8163 | 9.4056 | 0.0022 |
| ko04080 Neuroactive ligandreceptor interaction | 12.3518 | 0.3531 | 3.0646 | **0.0022** | 0.8224 | 9.3918 | 0.0022 |
| ko04390 Hippo signaling pathway | 12.2528 | 0.3524 | 3.0586 | **0.0023** | 0.8389 | 9.3553 | 0.0022 |
| ko00565 Ether lipid metabolism | 12.2219 | 0.3523 | 3.0582 | **0.0023** | 0.8402 | 9.3524 | 0.0022 |
| ko05217 Basal cell carcinoma | 12.2484 | 0.3522 | 3.0576 | **0.0023** | 0.8419 | 9.3488 | 0.0022 |
| ko04550 Signaling pathways regulating pluripotency of stem cells | 12.2371 | 0.3518 | 3.0534 | **0.0023** | 0.8536 | 9.3233 | 0.0023 |
| ko04927 Cortisol synthesis and secretion | 12.1516 | 0.3489 | 3.0288 | **0.0025** | 0.9260 | 9.1734 | 0.0025 |
| ko00983 Drug metabolism other enzymes | 12.1490 | 0.3479 | 3.0197 | **0.0026** | 0.9540 | 9.1186 | 0.0025 |
| ko02060 Phosphotransferase system PTS | 12.0170 | 0.3463 | 3.0056 | **0.0027** | 0.9994 | 9.0333 | 0.0027 |
| ko00510 NGlycan biosynthesis | 12.0929 | 0.3458 | 3.0019 | **0.0027** | 1.0000 | 9.0112 | 0.0027 |
| ko04072 Phospholipase D signaling pathway | 12.0348 | 0.3456 | 3.0001 | **0.0027** | 1.0000 | 9.0006 | 0.0027 |
| ko04922 Glucagon signaling pathway | 12.0270 | 0.3456 | 2.9999 | **0.0027** | 1.0000 | 8.9992 | 0.0027 |
| ko00460 Cyanoamino acid metabolism | 12.0522 | 0.3453 | 2.9973 | **0.0028** | 1.0000 | 8.9835 | 0.0027 |
| ko00625 Chloroalkane and chloroalkene degradation | 12.0254 | 0.3449 | 2.9939 | **0.0028** | 1.0000 | 8.9633 | 0.0028 |
| ko04024 cAMP signaling pathway | 12.0047 | 0.3447 | 2.9919 | **0.0028** | 1.0000 | 8.9512 | 0.0028 |
| ko00623 Toluene degradation | 11.8388 | 0.3430 | 2.9774 | **0.0029** | 1.0000 | 8.8651 | 0.0029 |
| ko00562 Inositol phosphate metabolism | 11.8920 | 0.3425 | 2.9731 | **0.0030** | 1.0000 | 8.8393 | 0.0029 |
| ko03013 RNA transport | 11.8723 | 0.3419 | 2.9682 | **0.0030** | 1.0000 | 8.8101 | 0.0030 |
| ko00780 Biotin metabolism | 11.9311 | 0.3418 | 2.9669 | **0.0030** | 1.0000 | 8.8026 | 0.0030 |
| ko04070 Phosphatidylinositol signaling system | 11.8641 | 0.3416 | 2.9651 | **0.0031** | 1.0000 | 8.7916 | 0.0030 |
| ko00450 Selenocompound metabolism | 11.7160 | 0.3380 | 2.9339 | **0.0034** | 1.0000 | 8.6078 | 0.0033 |
| ko00290 Valine leucine and isoleucine biosynthesis | 11.6845 | 0.3367 | 2.9231 | **0.0035** | 1.0000 | 8.5443 | 0.0035 |
| ko00970 AminoacyltRNA biosynthesis | 11.6639 | 0.3366 | 2.9220 | **0.0035** | 1.0000 | 8.5381 | 0.0035 |
| ko04913 Ovarian steroidogenesis | 11.5935 | 0.3361 | 2.9177 | **0.0036** | 1.0000 | 8.5132 | 0.0035 |
| ko00770 Pantothenate and CoA biosynthesis | 11.6564 | 0.3348 | 2.9061 | **0.0037** | 1.0000 | 8.4455 | 0.0037 |
| ko00982 Drug metabolism cytochrome P450 | 11.5191 | 0.3342 | 2.9005 | **0.0038** | 1.0000 | 8.4131 | 0.0037 |
| ko04022 cGMPPKG signaling pathway | 11.7199 | 0.3340 | 2.8993 | **0.0038** | 1.0000 | 8.4058 | 0.0037 |
| ko00622 Xylene degradation | 11.4907 | 0.3338 | 2.8978 | **0.0038** | 1.0000 | 8.3971 | 0.0038 |
| ko00401 Novobiocin biosynthesis | 11.5775 | 0.3337 | 2.8967 | **0.0038** | 1.0000 | 8.3909 | 0.0038 |
| ko00740 Riboflavin metabolism | 11.5594 | 0.3330 | 2.8907 | **0.0039** | 1.0000 | 8.3562 | 0.0038 |
| ko02010 ABC transporters | 11.5629 | 0.3318 | 2.8801 | **0.0040** | 1.0000 | 8.2952 | 0.0040 |
| ko00601 Glycosphingolipid biosynthesis lacto and | 11.4776 | 0.3306 | 2.8693 | **0.0042** | 1.0000 | 8.2329 | 0.0041 |

| | | | | | | | |
|---|---|---|---|---|---|---|---|
| neolacto series | | | | | | | |
| ko02024 Quorum sensing | 11.4648 | 0.3304 | 2.8676 | **0.0042** | 1.0000 | 8.2230 | 0.0041 |
| ko04261 Adrenergic signaling in cardiomyocytes | 11.5712 | 0.3303 | 2.8672 | **0.0042** | 1.0000 | 8.2210 | 0.0041 |
| ko04020 Calcium signaling pathway | 11.5545 | 0.3301 | 2.8656 | **0.0042** | 1.0000 | 8.2114 | 0.0042 |
| ko00195 Photosynthesis | 11.5808 | 0.3300 | 2.8646 | **0.0042** | 1.0000 | 8.2057 | 0.0042 |
| ko04728 Dopaminergic synapse | 11.4671 | 0.3289 | 2.8547 | **0.0044** | 1.0000 | 8.1494 | 0.0043 |
| ko00480 Glutathione metabolism | 11.3150 | 0.3276 | 2.8439 | **0.0045** | 1.0000 | 8.0878 | 0.0045 |
| ko04723 Retrograde endocannabinoid signaling | 11.2972 | 0.3270 | 2.8380 | **0.0046** | 1.0000 | 8.0545 | 0.0045 |
| ko04014 Ras signaling pathway | 11.3297 | 0.3263 | 2.8323 | **0.0047** | 1.0000 | 8.0217 | 0.0046 |
| ko00261 Monobactam biosynthesis | 11.2291 | 0.3259 | 2.8292 | **0.0047** | 1.0000 | 8.0045 | 0.0047 |
| ko04924 Renin secretion | 11.3931 | 0.3253 | 2.8239 | **0.0048** | 1.0000 | 7.9744 | 0.0047 |
| ko05034 Alcoholism | 11.3140 | 0.3252 | 2.8232 | **0.0048** | 1.0000 | 7.9704 | 0.0048 |
| ko05418 Fluid shear stress and atherosclerosis | 11.3720 | 0.3252 | 2.8229 | **0.0048** | 1.0000 | 7.9687 | 0.0048 |
| ko05160 Hepatitis C | 11.3077 | 0.3249 | 2.8202 | **0.0049** | 1.0000 | 7.9534 | 0.0048 |
| ko00130 Ubiquinone and other terpenoidquinone biosynthesis | 11.2350 | 0.3245 | 2.8163 | **0.0049** | 1.0000 | 7.9317 | 0.0049 |
| ko00100 Steroid biosynthesis | 11.2716 | 0.3240 | 2.8123 | **0.0050** | 1.0000 | 7.9089 | 0.0049 |
| ko00072 Synthesis and degradation of ketone bodies | 11.2428 | 0.3239 | 2.8117 | **0.0050** | 1.0000 | 7.9058 | 0.0049 |
| ko04371 Apelin signaling pathway | 11.3131 | 0.3238 | 2.8103 | **0.0050** | 1.0000 | 7.8975 | 0.0050 |
| ko04912 GnRH signaling pathway | 11.2032 | 0.3231 | 2.8049 | **0.0051** | 1.0000 | 7.8673 | 0.0050 |
| ko05031 Amphetamine addiction | 11.2247 | 0.3231 | 2.8043 | **0.0051** | 1.0000 | 7.8640 | 0.0050 |
| ko04270 Vascular smooth muscle contraction | 11.2949 | 0.3227 | 2.8011 | **0.0051** | 1.0000 | 7.8463 | 0.0051 |
| ko05170 Human immunodeficiency virus 1 infection | 11.2458 | 0.3223 | 2.7973 | **0.0052** | 1.0000 | 7.8249 | 0.0052 |
| ko00643 Styrene degradation | 11.2294 | 0.3221 | 2.7957 | **0.0052** | 1.0000 | 7.8160 | 0.0052 |
| ko01040 Biosynthesis of unsaturated fatty acids | 11.0993 | 0.3216 | 2.7914 | **0.0053** | 1.0000 | 7.7917 | 0.0052 |
| ko05152 Tuberculosis | 11.2426 | 0.3214 | 2.7901 | **0.0053** | 1.0000 | 7.7849 | 0.0053 |
| ko00633 Nitrotoluene degradation | 11.1661 | 0.3209 | 2.7852 | **0.0054** | 1.0000 | 7.7573 | 0.0053 |
| ko00513 Various types of Nglycan biosynthesis | 11.2348 | 0.3208 | 2.7849 | **0.0054** | 1.0000 | 7.7558 | 0.0054 |
| ko04218 Cellular senescence | 11.1697 | 0.3205 | 2.7818 | **0.0055** | 1.0000 | 7.7385 | 0.0054 |
| ko00750 Vitamin B6 metabolism | 11.1008 | 0.3202 | 2.7793 | **0.0055** | 1.0000 | 7.7247 | 0.0054 |
| ko04724 Glutamatergic synapse | 11.0438 | 0.3197 | 2.7752 | **0.0056** | 1.0000 | 7.7020 | 0.0055 |
| ko04216 Ferroptosis | 11.0531 | 0.3196 | 2.7746 | **0.0056** | 1.0000 | 7.6986 | 0.0055 |
| ko04217 Necroptosis | 11.0886 | 0.3191 | 2.7699 | **0.0057** | 1.0000 | 7.6725 | 0.0056 |
| ko05167 Kaposi sarcomaassociated herpesvirus infection | 11.1249 | 0.3185 | 2.7650 | **0.0057** | 1.0000 | 7.6454 | 0.0057 |
| ko04066 HIF1 signaling pathway | 11.0207 | 0.3169 | 2.7505 | **0.0060** | 1.0000 | 7.5651 | 0.0060 |
| ko05164 Influenza A | 11.0349 | 0.3164 | 2.7465 | **0.0061** | 1.0000 | 7.5432 | 0.0060 |
| ko05230 Central carbon metabolism in cancer | 10.9704 | 0.3158 | 2.7412 | **0.0062** | 1.0000 | 7.5142 | 0.0061 |

| | | | | | | | |
|---|---|---|---|---|---|---|---|
| ko04744 Phototransduction | 11.0044 | 0.3158 | 2.7410 | **0.0062** | 1.0000 | 7.5133 | 0.0061 |
| ko04921 Oxytocin signaling pathway | 11.0114 | 0.3156 | 2.7392 | **0.0062** | 1.0000 | 7.5030 | 0.0062 |
| ko05163 Human cytomegalovirus infection | 11.0131 | 0.3154 | 2.7375 | **0.0062** | 1.0000 | 7.4942 | 0.0062 |
| ko03410 Base excision repair | 10.8718 | 0.3147 | 2.7313 | **0.0064** | 1.0000 | 7.4603 | 0.0063 |
| ko04666 Fc gamma Rmediated phagocytosis | 10.8708 | 0.3145 | 2.7303 | **0.0064** | 1.0000 | 7.4545 | 0.0063 |
| ko05231 Choline metabolism in cancer | 10.8680 | 0.3144 | 2.7294 | **0.0064** | 1.0000 | 7.4498 | 0.0063 |
| ko00532 Glycosaminoglycan biosynthesis chondroitin sulfate dermatan sulfate | 10.9318 | 0.3123 | 2.7109 | **0.0068** | 1.0000 | 7.3492 | 0.0067 |
| ko04210 Apoptosis | 10.9654 | 0.3122 | 2.7096 | **0.0068** | 1.0000 | 7.3420 | 0.0067 |
| ko04010 MAPK signaling pathway | 10.8466 | 0.3117 | 2.7055 | **0.0069** | 1.0000 | 7.3197 | 0.0068 |
| ko00920 Sulfur metabolism | 10.8356 | 0.3114 | 2.7034 | **0.0069** | 1.0000 | 7.3084 | 0.0069 |
| ko04931 Insulin resistance | 10.7860 | 0.3111 | 2.7004 | **0.0070** | 1.0000 | 7.2921 | 0.0069 |
| ko04976 Bile secretion | 10.9040 | 0.3110 | 2.6999 | **0.0070** | 1.0000 | 7.2892 | 0.0069 |
| ko04971 Gastric acid secretion | 10.8360 | 0.3108 | 2.6974 | **0.0071** | 1.0000 | 7.2761 | 0.0070 |
| ko05120 Epithelial cell signaling in Helicobacter pylori infection | 10.7853 | 0.3107 | 2.6971 | **0.0071** | 1.0000 | 7.2743 | 0.0070 |
| ko05323 Rheumatoid arthritis | 10.7117 | 0.3107 | 2.6970 | **0.0071** | 1.0000 | 7.2738 | 0.0070 |
| ko04928 Parathyroid hormone synthesis secretion and action | 10.6984 | 0.3105 | 2.6948 | **0.0071** | 1.0000 | 7.2621 | 0.0070 |
| ko04722 Neurotrophin signaling pathway | 10.8205 | 0.3102 | 2.6923 | **0.0072** | 1.0000 | 7.2485 | 0.0071 |
| ko04970 Salivary secretion | 10.8773 | 0.3100 | 2.6909 | **0.0072** | 1.0000 | 7.2410 | 0.0071 |
| ko04910 Insulin signaling pathway | 10.8057 | 0.3094 | 2.6858 | **0.0073** | 1.0000 | 7.2134 | 0.0072 |
| ko04145 Phagosome | 10.7494 | 0.3094 | 2.6857 | **0.0073** | 1.0000 | 7.2130 | 0.0072 |
| ko00534 Glycosaminoglycan biosynthesis heparan sulfate heparin | 10.7436 | 0.3091 | 2.6828 | **0.0074** | 1.0000 | 7.1974 | 0.0073 |
| ko05214 Glioma | 10.7554 | 0.3087 | 2.6795 | **0.0074** | 1.0000 | 7.1800 | 0.0074 |
| ko04015 Rap1 signaling pathway | 10.7519 | 0.3085 | 2.6779 | **0.0075** | 1.0000 | 7.1710 | 0.0074 |
| ko00281 Geraniol degradation | 10.6773 | 0.3082 | 2.6749 | **0.0075** | 1.0000 | 7.1553 | 0.0075 |
| ko04713 Circadian entrainment | 10.7481 | 0.3080 | 2.6736 | **0.0076** | 1.0000 | 7.1481 | 0.0075 |
| ko00361 Chlorocyclohexane and chlorobenzene degradation | 10.7699 | 0.3079 | 2.6727 | **0.0076** | 1.0000 | 7.1434 | 0.0075 |
| ko04915 Estrogen signaling pathway | 10.7458 | 0.3074 | 2.6687 | **0.0077** | 1.0000 | 7.1219 | 0.0076 |
| ko05202 Transcriptional misregulation in cancer | 10.7172 | 0.3074 | 2.6684 | **0.0077** | 1.0000 | 7.1202 | 0.0076 |
| ko00660 C5Branched dibasic acid metabolism | 10.6960 | 0.3073 | 2.6677 | **0.0077** | 1.0000 | 7.1168 | 0.0076 |
| ko00120 Primary bile acid biosynthesis | 10.6524 | 0.3070 | 2.6650 | **0.0078** | 1.0000 | 7.1022 | 0.0077 |
| ko05133 Pertussis | 10.7129 | 0.3070 | 2.6648 | **0.0078** | 1.0000 | 7.1013 | 0.0077 |
| ko05110 Vibrio cholerae infection | 10.5930 | 0.3069 | 2.6643 | **0.0078** | 1.0000 | 7.0983 | 0.0077 |
| ko00960 Tropane piperidine and pyridine alkaloid biosynthesis | 10.6420 | 0.3063 | 2.6590 | **0.0079** | 1.0000 | 7.0702 | 0.0078 |

| | | | | | | | |
|---|---|---|---|---|---|---|---|
| ko00333 Prodigiosin biosynthesis | 10.5043 | 0.3053 | 2.6503 | **0.0081** | 1.0000 | 7.0243 | 0.0080 |
| ko04721 Synaptic vesicle cycle | 10.6025 | 0.3053 | 2.6499 | **0.0081** | 1.0000 | 7.0221 | 0.0081 |
| ko04625 Ctype lectin receptor signaling pathway | 10.6021 | 0.3047 | 2.6452 | **0.0082** | 1.0000 | 6.9971 | 0.0082 |
| ko00930 Caprolactam degradation | 10.5541 | 0.3038 | 2.6371 | **0.0084** | 1.0000 | 6.9542 | 0.0084 |
| ko04114 Oocyte meiosis | 10.5554 | 0.3038 | 2.6367 | **0.0084** | 1.0000 | 6.9521 | 0.0084 |
| ko04740 Olfactory transduction | 10.5622 | 0.3033 | 2.6329 | **0.0085** | 1.0000 | 6.9324 | 0.0085 |
| ko04626 Plantpathogen interaction | 10.5455 | 0.3032 | 2.6322 | **0.0086** | 1.0000 | 6.9287 | 0.0085 |
| ko00981 Insect hormone biosynthesis | 10.4750 | 0.3029 | 2.6294 | **0.0086** | 1.0000 | 6.9138 | 0.0086 |
| ko04071 Sphingolipid signaling pathway | 10.5096 | 0.3027 | 2.6278 | **0.0087** | 1.0000 | 6.9056 | 0.0086 |
| ko00603 Glycosphingolipid biosynthesis globo and isoglobo series | 10.5212 | 0.3025 | 2.6262 | **0.0087** | 1.0000 | 6.8968 | 0.0086 |
| ko04720 Longterm potentiation | 10.5240 | 0.3025 | 2.6261 | **0.0087** | 1.0000 | 6.8962 | 0.0086 |
| ko04745 Phototransduction fly | 10.5111 | 0.3023 | 2.6237 | **0.0088** | 1.0000 | 6.8839 | 0.0087 |
| ko01053 Biosynthesis of siderophore group nonribosomal peptides | 10.4496 | 0.3022 | 2.6235 | **0.0088** | 1.0000 | 6.8827 | 0.0087 |
| ko04016 MAPK signaling pathway plant | 10.5075 | 0.3022 | 2.6227 | **0.0088** | 1.0000 | 6.8788 | 0.0087 |
| ko00062 Fatty acid elongation | 10.5207 | 0.3017 | 2.6191 | **0.0089** | 1.0000 | 6.8595 | 0.0088 |
| ko03030 DNA replication | 10.4395 | 0.3017 | 2.6186 | **0.0089** | 1.0000 | 6.8572 | 0.0088 |
| ko00940 Phenylpropanoid biosynthesis | 10.4862 | 0.3013 | 2.6149 | **0.0090** | 1.0000 | 6.8378 | 0.0089 |
| ko00965 Betalain biosynthesis | 10.3884 | 0.3009 | 2.6119 | **0.0091** | 1.0000 | 6.8221 | 0.0090 |
| ko04360 Axon guidance | 10.4406 | 0.3004 | 2.6073 | **0.0092** | 1.0000 | 6.7981 | 0.0091 |
| ko04966 Collecting duct acid secretion | 10.3426 | 0.3003 | 2.6065 | **0.0092** | 1.0000 | 6.7940 | 0.0091 |
| ko01501 betaLactam resistance | 10.4288 | 0.3002 | 2.6061 | **0.0092** | 1.0000 | 6.7919 | 0.0092 |
| ko04011 MAPK signaling pathway yeast | 10.4788 | 0.3001 | 2.6049 | **0.0093** | 1.0000 | 6.7856 | 0.0092 |
| ko03008 Ribosome biogenesis in eukaryotes | 10.5213 | 0.2998 | 2.6024 | **0.0093** | 1.0000 | 6.7724 | 0.0093 |
| ko03020 RNA polymerase | 10.4366 | 0.2994 | 2.5991 | **0.0094** | 1.0000 | 6.7552 | 0.0093 |
| ko04920 Adipocytokine signaling pathway | 10.2880 | 0.2980 | 2.5871 | **0.0098** | 1.0000 | 6.6932 | 0.0097 |
| ko00592 alphaLinolenic acid metabolism | 10.2834 | 0.2966 | 2.5744 | **0.0101** | 1.0000 | 6.6278 | 0.0100 |
| ko00830 Retinol metabolism | 10.1324 | 0.2946 | 2.5575 | **0.0106** | 1.0000 | 6.5409 | 0.0105 |
| ko04350 TGFbeta signaling pathway | 10.1761 | 0.2945 | 2.5567 | **0.0106** | 1.0000 | 6.5367 | 0.0106 |
| ko04144 Endocytosis | 10.2736 | 0.2940 | 2.5523 | **0.0108** | 1.0000 | 6.5140 | 0.0107 |
| ko04974 Protein digestion and absorption | 10.2197 | 0.2936 | 2.5485 | **0.0109** | 1.0000 | 6.4947 | 0.0108 |
| ko04614 Reninangiotensin system | 10.3522 | 0.2932 | 2.5448 | **0.0110** | 1.0000 | 6.4760 | 0.0109 |
| ko00550 Peptidoglycan biosynthesis | 10.2138 | 0.2930 | 2.5432 | **0.0111** | 1.0000 | 6.4677 | 0.0110 |

| | | | | | | | |
|---|---|---|---|---|---|---|---|
| ko04961 Endocrine and other factorregulated calcium reabsorption | 10.2808 | 0.2929 | 2.5423 | **0.0111** | 1.0000 | 6.4633 | 0.0110 |
| ko04972 Pancreatic secretion | 10.1595 | 0.2923 | 2.5376 | **0.0112** | 1.0000 | 6.4393 | 0.0112 |
| ko04152 AMPK signaling pathway | 10.2433 | 0.2922 | 2.5366 | **0.0113** | 1.0000 | 6.4345 | 0.0112 |
| ko01503 Cationic antimicrobial peptide CAMP resistance | 10.1274 | 0.2901 | 2.5182 | **0.0119** | 1.0000 | 6.3413 | 0.0118 |
| ko00730 Thiamine metabolism | 10.0787 | 0.2897 | 2.5151 | **0.0120** | 1.0000 | 6.3256 | 0.0119 |
| ko04933 AGERAGE signaling pathway in diabetic complications | 10.1831 | 0.2896 | 2.5137 | **0.0120** | 1.0000 | 6.3185 | 0.0119 |
| ko04520 Adherens junction | 9.9389 | 0.2887 | 2.5058 | **0.0123** | 1.0000 | 6.2793 | 0.0122 |
| ko05143 African trypanosomiasis | 9.9454 | 0.2885 | 2.5045 | **0.0123** | 1.0000 | 6.2727 | 0.0123 |
| ko04610 Complement and coagulation cascades | 10.0311 | 0.2879 | 2.4989 | **0.0125** | 1.0000 | 6.2446 | 0.0125 |
| ko05030 Cocaine addiction | 9.9692 | 0.2878 | 2.4984 | **0.0126** | 1.0000 | 6.2420 | 0.0125 |
| ko04111 Cell cycle yeast | 10.0059 | 0.2873 | 2.4934 | **0.0127** | 1.0000 | 6.2171 | 0.0127 |
| ko05145 Toxoplasmosis | 9.9502 | 0.2866 | 2.4878 | **0.0129** | 1.0000 | 6.1892 | 0.0129 |
| ko00903 Limonene and pinene degradation | 9.8856 | 0.2864 | 2.4861 | **0.0130** | 1.0000 | 6.1809 | 0.0129 |
| ko04068 FoxO signaling pathway | 9.8925 | 0.2863 | 2.4855 | **0.0130** | 1.0000 | 6.1776 | 0.0129 |
| ko04978 Mineral absorption | 10.0600 | 0.2849 | 2.4733 | **0.0135** | 1.0000 | 6.1170 | 0.0134 |
| ko04110 Cell cycle | 9.8905 | 0.2848 | 2.4721 | **0.0135** | 1.0000 | 6.1113 | 0.0134 |
| ko00944 Flavone and flavonol biosynthesis | 9.8216 | 0.2833 | 2.4590 | **0.0140** | 1.0000 | 6.0465 | 0.0139 |
| ko05169 EpsteinBarr virus infection | 9.9873 | 0.2832 | 2.4584 | **0.0140** | 1.0000 | 6.0438 | 0.0140 |
| ko04975 Fat digestion and absorption | 9.8233 | 0.2831 | 2.4574 | **0.0141** | 1.0000 | 6.0389 | 0.0140 |
| ko05168 Herpes simplex infection | 9.9430 | 0.2830 | 2.4562 | **0.0141** | 1.0000 | 6.0329 | 0.0140 |
| ko04979 Cholesterol metabolism | 9.7852 | 0.2827 | 2.4537 | **0.0142** | 1.0000 | 6.0204 | 0.0141 |
| ko04113 Meiosis yeast | 9.8353 | 0.2826 | 2.4528 | **0.0143** | 1.0000 | 6.0162 | 0.0142 |
| ko00591 Linoleic acid metabolism | 9.7838 | 0.2805 | 2.4345 | **0.0150** | 1.0000 | 5.9267 | 0.0149 |
| ko04664 Fc epsilon RI signaling pathway | 9.6390 | 0.2781 | 2.4140 | **0.0159** | 1.0000 | 5.8276 | 0.0158 |
| ko00906 Carotenoid biosynthesis | 9.5809 | 0.2776 | 2.4093 | **0.0161** | 1.0000 | 5.8049 | 0.0160 |
| ko03440 Homologous recombination | 9.5738 | 0.2770 | 2.4046 | **0.0163** | 1.0000 | 5.7823 | 0.0162 |
| ko00364 Fluorobenzoate degradation | 9.6091 | 0.2770 | 2.4044 | **0.0163** | 1.0000 | 5.7812 | 0.0162 |
| ko04060 Cytokinecytokine receptor interaction | 9.7688 | 0.2766 | 2.4010 | **0.0165** | 1.0000 | 5.7646 | 0.0164 |
| ko01502 Vancomycin resistance | 9.5630 | 0.2759 | 2.3946 | **0.0167** | 1.0000 | 5.7342 | 0.0166 |
| ko05203 Viral carcinogenesis | 9.5608 | 0.2752 | 2.3884 | **0.0170** | 1.0000 | 5.7045 | 0.0169 |
| ko00232 Caffeine metabolism | 9.4588 | 0.2746 | 2.3834 | **0.0173** | 1.0000 | 5.6806 | 0.0172 |
| ko04122 Sulfur relay system | 9.4698 | 0.2741 | 2.3793 | **0.0174** | 1.0000 | 5.6611 | 0.0173 |
| ko05215 Prostate cancer | 9.2776 | 0.2733 | 2.3726 | **0.0178** | 1.0000 | 5.6295 | 0.0177 |
| ko00471 DGlutamine and Dglutamate metabolism | 9.5112 | 0.2733 | 2.3723 | **0.0178** | 1.0000 | 5.6280 | 0.0177 |

| | | | | | | | |
|---|---|---|---|---|---|---|---|
| ko03430 Mismatch repair | 9.5082 | 0.2733 | 2.3723 | **0.0178** | 1.0000 | 5.6278 | 0.0177 |
| ko04064 NFkappa B signaling pathway | 9.4991 | 0.2732 | 2.3712 | **0.0178** | 1.0000 | 5.6228 | 0.0177 |
| ko00430 Taurine and hypotaurine metabolism | 9.6385 | 0.2727 | 2.3667 | **0.0180** | 1.0000 | 5.6012 | 0.0179 |
| ko03450 Nonhomologous endjoining | 9.2931 | 0.2707 | 2.3501 | **0.0189** | 1.0000 | 5.5232 | 0.0188 |
| ko04668 TNF signaling pathway | 9.3330 | 0.2695 | 2.3392 | **0.0194** | 1.0000 | 5.4721 | 0.0193 |
| ko04725 Cholinergic synapse | 9.3695 | 0.2694 | 2.3384 | **0.0195** | 1.0000 | 5.4680 | 0.0194 |
| ko04964 Proximal tubule bicarbonate reclamation | 9.4919 | 0.2692 | 2.3367 | **0.0196** | 1.0000 | 5.4603 | 0.0195 |
| ko03050 Proteasome | 9.4400 | 0.2688 | 2.3333 | **0.0197** | 1.0000 | 5.4444 | 0.0196 |
| ko00563 Glycosylphosphatidylinositol GPIanchor biosynthesis | 9.3850 | 0.2684 | 2.3296 | **0.0199** | 1.0000 | 5.4271 | 0.0198 |
| ko00540 Lipopolysaccharide biosynthesis | 9.3851 | 0.2680 | 2.3262 | **0.0201** | 1.0000 | 5.4113 | 0.0200 |
| ko04115 p53 signaling pathway | 9.3194 | 0.2677 | 2.3236 | **0.0203** | 1.0000 | 5.3990 | 0.0201 |
| ko00533 Glycosaminoglycan biosynthesis keratan sulfate | 9.2957 | 0.2667 | 2.3155 | **0.0207** | 1.0000 | 5.3613 | 0.0206 |
| ko02026 Biofilm formation Escherichia coli | 9.2178 | 0.2658 | 2.3076 | **0.0211** | 1.0000 | 5.3248 | 0.0210 |
| ko04919 Thyroid hormone signaling pathway | 9.3468 | 0.2654 | 2.3040 | **0.0213** | 1.0000 | 5.3085 | 0.0212 |
| ko05206 MicroRNAs in cancer | 9.2839 | 0.2654 | 2.3038 | **0.0213** | 1.0000 | 5.3075 | 0.0212 |
| ko02040 Flagellar assembly | 9.1795 | 0.2646 | 2.2965 | **0.0218** | 1.0000 | 5.2738 | 0.0216 |
| ko03022 Basal transcription factors | 9.2065 | 0.2636 | 2.2885 | **0.0222** | 1.0000 | 5.2374 | 0.0221 |
| ko05340 Primary immunodeficiency | 9.1828 | 0.2635 | 2.2876 | **0.0223** | 1.0000 | 5.2330 | 0.0222 |
| ko00332 Carbapenem biosynthesis | 8.9514 | 0.2633 | 2.2856 | **0.0224** | 1.0000 | 5.2240 | 0.0223 |
| ko05014 Amyotrophic lateral sclerosis ALS | 9.1190 | 0.2619 | 2.2735 | **0.0231** | 1.0000 | 5.1686 | 0.0230 |
| ko05322 Systemic lupus erythematosus | 9.1501 | 0.2619 | 2.2732 | **0.0231** | 1.0000 | 5.1675 | 0.0230 |
| ko03420 Nucleotide excision repair | 9.1025 | 0.2612 | 2.2670 | **0.0235** | 1.0000 | 5.1393 | 0.0234 |
| ko04141 Protein processing in endoplasmic reticulum | 9.0807 | 0.2592 | 2.2501 | **0.0246** | 1.0000 | 5.0630 | 0.0244 |
| ko05410 Hypertrophic cardiomyopathy HCM | 8.9619 | 0.2585 | 2.2443 | **0.0249** | 1.0000 | 5.0367 | 0.0248 |
| ko05412 Arrhythmogenic right ventricular cardiomyopathy ARVC | 8.8057 | 0.2576 | 2.2359 | **0.0255** | 1.0000 | 4.9994 | 0.0254 |
| ko04151 PI3KAkt signaling pathway | 9.0716 | 0.2573 | 2.2330 | **0.0257** | 1.0000 | 4.9864 | 0.0255 |
| ko04621 NODlike receptor signaling pathway | 9.0567 | 0.2547 | 2.2113 | **0.0271** | 1.0000 | 4.8898 | 0.0270 |
| ko05033 Nicotine addiction | 8.7518 | 0.2547 | 2.2108 | **0.0272** | 1.0000 | 4.8874 | 0.0271 |
| ko04917 Prolactin signaling pathway | 8.8757 | 0.2547 | 2.2106 | **0.0272** | 1.0000 | 4.8866 | 0.0271 |
| ko00473 DAlanine metabolism | 8.8097 | 0.2546 | 2.2100 | **0.0272** | 1.0000 | 4.8841 | 0.0271 |
| ko00512 Mucin type Oglycan biosynthesis | 8.9311 | 0.2543 | 2.2076 | **0.0274** | 1.0000 | 4.8736 | 0.0273 |

| | | | | | | | |
|---|---|---|---|---|---|---|---|
| ko05142 Chagas disease American trypanosomiasis | 8.8125 | 0.2539 | 2.2038 | **0.0277** | 1.0000 | 4.8567 | 0.0275 |
| ko04973 Carbohydrate digestion and absorption | 8.9084 | 0.2530 | 2.1961 | **0.0282** | 1.0000 | 4.8226 | 0.0281 |
| ko00901 Indole alkaloid biosynthesis | 8.5864 | 0.2527 | 2.1932 | **0.0284** | 1.0000 | 4.8101 | 0.0283 |
| ko04215 Apoptosis multiple species | 8.7631 | 0.2525 | 2.1921 | **0.0285** | 1.0000 | 4.8054 | 0.0284 |
| ko05222 Small cell lung cancer | 8.7733 | 0.2520 | 2.1876 | **0.0288** | 1.0000 | 4.7857 | 0.0287 |
| ko00642 Ethylbenzene degradation | 8.7689 | 0.2519 | 2.1869 | **0.0289** | 1.0000 | 4.7824 | 0.0288 |
| ko05223 Nonsmall cell lung cancer | 8.6414 | 0.2476 | 2.1496 | **0.0317** | 1.0000 | 4.6210 | 0.0316 |
| ko04612 Antigen processing and presentation | 8.8177 | 0.2468 | 2.1425 | **0.0323** | 1.0000 | 4.5903 | 0.0322 |
| ko04212 Longevity regulating pathway worm | 8.5362 | 0.2467 | 2.1417 | **0.0323** | 1.0000 | 4.5870 | 0.0322 |
| ko03070 Bacterial secretion system | 8.8062 | 0.2466 | 2.1405 | **0.0324** | 1.0000 | 4.5817 | 0.0323 |
| ko04138 Autophagy yeast | 8.8613 | 0.2462 | 2.1373 | **0.0327** | 1.0000 | 4.5681 | 0.0326 |
| ko04320 Dorsoventral axis formation | 8.6289 | 0.2461 | 2.1366 | **0.0328** | 1.0000 | 4.5650 | 0.0326 |
| ko04911 Insulin secretion | 8.7287 | 0.2461 | 2.1362 | **0.0328** | 1.0000 | 4.5632 | 0.0327 |
| ko04657 IL17 signaling pathway | 8.4933 | 0.2450 | 2.1263 | **0.0336** | 1.0000 | 4.5213 | 0.0335 |
| ko00941 Flavonoid biosynthesis | 8.6010 | 0.2433 | 2.1120 | **0.0348** | 1.0000 | 4.4607 | 0.0347 |
| ko03060 Protein export | 8.9145 | 0.2433 | 2.1119 | **0.0348** | 1.0000 | 4.4602 | 0.0347 |
| ko05414 Dilated cardiomyopathy DCM | 8.3430 | 0.2430 | 2.1091 | **0.0351** | 1.0000 | 4.4483 | 0.0349 |
| ko04112 Cell cycle Caulobacter | 8.4160 | 0.2413 | 2.0950 | **0.0363** | 1.0000 | 4.3888 | 0.0362 |
| ko00514 Other types of Oglycan biosynthesis | 8.4763 | 0.2412 | 2.0939 | **0.0364** | 1.0000 | 4.3845 | 0.0363 |
| ko04062 Chemokine signaling pathway | 8.6717 | 0.2407 | 2.0891 | **0.0368** | 1.0000 | 4.3643 | 0.0367 |
| ko04630 JAKSTAT signaling pathway | 8.3258 | 0.2401 | 2.0843 | **0.0373** | 1.0000 | 4.3441 | 0.0371 |
| ko05161 Hepatitis B | 8.3531 | 0.2392 | 2.0765 | **0.0380** | 1.0000 | 4.3117 | 0.0379 |
| ko05134 Legionellosis | 8.2638 | 0.2372 | 2.0593 | **0.0396** | 1.0000 | 4.2406 | 0.0395 |
| ko04380 Osteoclast differentiation | 8.1820 | 0.2370 | 2.0573 | **0.0398** | 1.0000 | 4.2326 | 0.0397 |
| ko04960 Aldosteroneregulated sodium reabsorption | 8.3848 | 0.2367 | 2.0547 | **0.0400** | 1.0000 | 4.2218 | 0.0399 |
| ko04977 Vitamin digestion and absorption | 8.2621 | 0.2347 | 2.0375 | **0.0417** | 1.0000 | 4.1513 | 0.0416 |
| ko00521 Streptomycin biosynthesis | 8.1185 | 0.2346 | 2.0362 | **0.0419** | 1.0000 | 4.1463 | 0.0417 |
| ko00515 Mannose type Oglycan biosynthesis | 8.2088 | 0.2345 | 2.0356 | **0.0419** | 1.0000 | 4.1437 | 0.0418 |
| ko05219 Bladder cancer | 8.0989 | 0.2343 | 2.0337 | **0.0421** | 1.0000 | 4.1360 | 0.0420 |
| ko04918 Thyroid hormone synthesis | 8.2716 | 0.2341 | 2.0316 | **0.0423** | 1.0000 | 4.1276 | 0.0422 |
| ko00945 Stilbenoid diarylheptanoid and gingerol biosynthesis | 8.3038 | 0.2339 | 2.0305 | **0.0424** | 1.0000 | 4.1229 | 0.0423 |
| ko03018 RNA degradation | 8.2000 | 0.2337 | 2.0290 | **0.0426** | 1.0000 | 4.1168 | 0.0425 |

| | | | | | | | |
|---|---|---|---|---|---|---|---|
| ko05111 Biofilm formation Vibrio cholerae | 8.1061 | 0.2328 | 2.0205 | **0.0435** | 1.0000 | 4.0823 | 0.0433 |
| ko01521 EGFR tyrosine kinase inhibitor resistance | 8.1712 | 0.2326 | 2.0191 | **0.0436** | 1.0000 | 4.0766 | 0.0435 |
| ko04214 Apoptosis fly | 8.0990 | 0.2320 | 2.0142 | **0.0441** | 1.0000 | 4.0570 | 0.0440 |
| ko04742 Taste transduction | 8.0448 | 0.2314 | 2.0086 | **0.0447** | 1.0000 | 4.0347 | 0.0446 |
| ko04659 Th17 cell differentiation | 8.0071 | 0.2283 | 1.9821 | **0.0476** | 1.0000 | 3.9287 | 0.0475 |
| ko04510 Focal adhesion | 7.9041 | 0.2276 | 1.9759 | **0.0483** | 1.0000 | 3.9040 | 0.0482 |
| ko04660 T cell receptor signaling pathway | 7.9551 | 0.2275 | 1.9744 | **0.0485** | 1.0000 | 3.8982 | 0.0483 |
| ko00908 Zeatin biosynthesis | 7.9121 | 0.2272 | 1.9721 | **0.0487** | 1.0000 | 3.8894 | 0.0486 |
| ko05150 Staphylococcus aureus infection | 8.0775 | 0.2256 | 1.9585 | 0.0503 | 1.0000 | 3.8358 | 0.0502 |
| ko00909 Sesquiterpenoid and triterpenoid biosynthesis | 7.7929 | 0.2254 | 1.9563 | 0.0506 | 1.0000 | 3.8272 | 0.0504 |
| ko02030 Bacterial chemotaxis | 7.8896 | 0.2252 | 1.9546 | 0.0508 | 1.0000 | 3.8206 | 0.0506 |
| ko05162 Measles | 7.8443 | 0.2240 | 1.9442 | 0.0520 | 1.0000 | 3.7799 | 0.0519 |
| ko04650 Natural killer cell mediated cytotoxicity | 7.7751 | 0.2224 | 1.9303 | 0.0537 | 1.0000 | 3.7260 | 0.0536 |
| ko04540 Gap junction | 7.9189 | 0.2223 | 1.9295 | 0.0538 | 1.0000 | 3.7228 | 0.0537 |
| ko04140 Autophagy animal | 7.7823 | 0.2187 | 1.8987 | 0.0578 | 1.0000 | 3.6051 | 0.0576 |
| LFC = log fold change. *limma* software R program. Log2 fold change calculated for each time point. Weeks 0, 3, 13, 26, 39, 52 were examined across six time points. Functional gene pathways filtered at (>0.01%) for analysis. | | | | | | | |

## Longitudinal Assessment of Fecal Targeted Short-Chain-Fatty-Acids Metabolomics

Fecal targeted SCFA metabolomics concentration changes over time for Acetate (mM/kg), Propionate (mM/kg), Butyrate (mM/kg), Total SCFA (mM/kg), and Total Butyrate-to-Total SCFA ratio (mM/kg), in the RRMS FMT subject were examined with results shown (**Table S9, Figure S9A-E**). All five targeted SCFA concentration measurements started high, stayed within the classified normal SCFA range, and remained above the observational household spouse control.

**Supplementary Table 9.** Fecal targeted short-chain-fatty-acids metabolomics concentration changes over time for acetate (mM/kg), propionate (mM/kg), butyrate (mM/kg), total SCFA (mM/kg), and total butyrate-to-total SCFA ratio (mM/kg), in the RRMS FMT subject.

| Multiple Comparison Test | Acetate | Propionate | Butyrate | Total SCFA | Total Butyrate to Total SCFA Ratio |
|---|---|---|---|---|---|
| F (Treatment) & *P*-Value comparisons between baseline and weeks after FMT | **$F_{(5,10)}$ = 54.21 <0.0001** | **$F_{(5,10)}$ = 136.7 <0.0001** | **$F_{(5,10)}$ = 154.5 <0.0001** | **$F_{(5,10)}$ = 88.81 <0.0001** | **$F_{(5,10)}$ = 426.8 <0.0001** |
| *Bonferroni Post-hoc Test P-Values and Week Mean Average Comparisons in Brackets* | | | | | |
| 0wk vs 3wk | **0.0352 [53.21, 48.19]** | **0.0020 [14.69, 11.86]** | >0.9999 [12.21, 11.58] | 0.0547 [80.11, 71.63] | 0.1141 [15.24, 16.16] |
| 0wk vs 13wk | 0.1176 [53.21, 57.32] | **0.0005 [14.69, 18.02]** | **<0.0001 [12.21, 20.13]** | **0.0007 [80.11, 95.46]** | **<0.0001 [15.24, 21.07]** |
| 0wk vs 26wk | **0.0004 [53.21, 44.11]** | **<0.0001 [14.69, 8.75]** | **<0.0001 [12.21, 5.04]** | **<0.0001 [80.11, 57.90]** | **<0.0001 [15.24, 8.70]** |
| 0wk vs 39wk | **0.0207 [53.21, 47.77]** | >0.9999 [14.69, 15.04] | >0.9999 [12.21, 13.00] | >0.9999 [80.11, 75.81] | **0.0007 [15.24, 17.14]** |
| 0wk vs. 52wk | **<0.0001 [53.21, 39.06]** | **<0.0001 [14.69, 7.98]** | **0.0003 [12.21, 7.79]** | **<0.0001 [80.11, 54.83]** | 0.0590 [15.24, 14.21] |
| 3wk vs. 13wk | **0.0004 [48.19, 57.32]** | **<0.0001 [11.86, 18.02]** | **<0.0001 [11.58, 20.13]** | **<0.0001 [71.63, 95.46]** | **<0.0001 [16.16, 21.07]** |
| 3wk vs. 26wk | 0.1219 [48.19, 44.11] | **0.0010 [11.86, 8.75]** | **<0.0001 [11.58, 5.04]** | **0.0017 [71.63, 57.90]** | **<0.0001 [16.16, 8.70]** |
| 3wk vs. 39wk | >0.9999 [48.19, 47.77] | **0.0008 [11.86, 15.04]** | 0.5367 [11.58, 13.00] | >0.9999 [71.63, 75.81] | 0.0814 [16.16, 17.14] |
| 3wk vs. 52wk | **0.0004 [48.19, 39.06]** | **0.0001 [11.86, 7.98]** | **0.0011 [11.58, 7.79]** | **0.0003 [71.63, 54.83]** | **0.0005 [16.16, 14.21]** |
| 13wk vs. 26wk | **<0.0001 [57.32, 44.11]** | **<0.0001 [18.02, 8.75]** | **<0.0001 [20.13, 5.04]** | **<0.0001 [95.46, 57.90]** | **<0.0001 [21.07, 8.70]** |
| 13wk vs. 39wk | **0.0002 [57.32, 47.77]** | **0.0013 [18.02, 15.04]** | **<0.0001 [20.13, 13.00]** | **<0.0001 [95.46, 75.81]** | **<0.0001 [21.07, 17.14]** |
| 13wk vs. 52wk | **<0.0001 [57.32, 39.06]** | **<0.0001 [18.02, 7.98]** | **<0.0001 [20.13, 7.79]** | **<0.0001 [95.46, 54.83]** | **<0.0001 [21.07, 14.21]** |
| 26wk vs. 39wk | 0.2159 [44.11, 47.77] | **<0.0001 [8.75, 15.04]** | **<0.0001 [5.04, 13.00]** | **0.0002 [57.90, 75.81]** | **<0.0001 [8.70, 17.14]** |
| 26wk vs. 52wk | **0.0340 [44.11, 39.06]** | >0.9999 [8.75, 7.98] | **0.0127 [5.04, 7.79]** | >0.9999 [57.90, 54.83] | **<0.0001 [8.70, 14.21]** |
| 39wk vs. 52wk | **0.0005 [47.77, 39.06]** | **<0.0001 [15.04, 7.98]** | **<0.0001 [13.00, 7.79]** | **<0.0001 [75.81, 54.83]** | **<0.0001 [17.14, 14.21]** |
| Parametric repeated measures 1-way ANOVA, based on Shapiro Wilk normality test of residuals and Levene homogeneity of variance, provided a p-value for each test. Post-hoc test of Bonferroni provided an adjusted p-value, along with each collection visit's mean average comparison in brackets. Bold font indicates statistically significant results. A total of 3 measurements per visit number. Visit numbers: (0 = baseline; 3 = 3 weeks; 13 = 13 weeks; 26 = 26 weeks; 39 = 39 weeks; 52 = 52 weeks). Targeted metabolomics of short-chain-fatty-acids (SCFA). | | | | | |

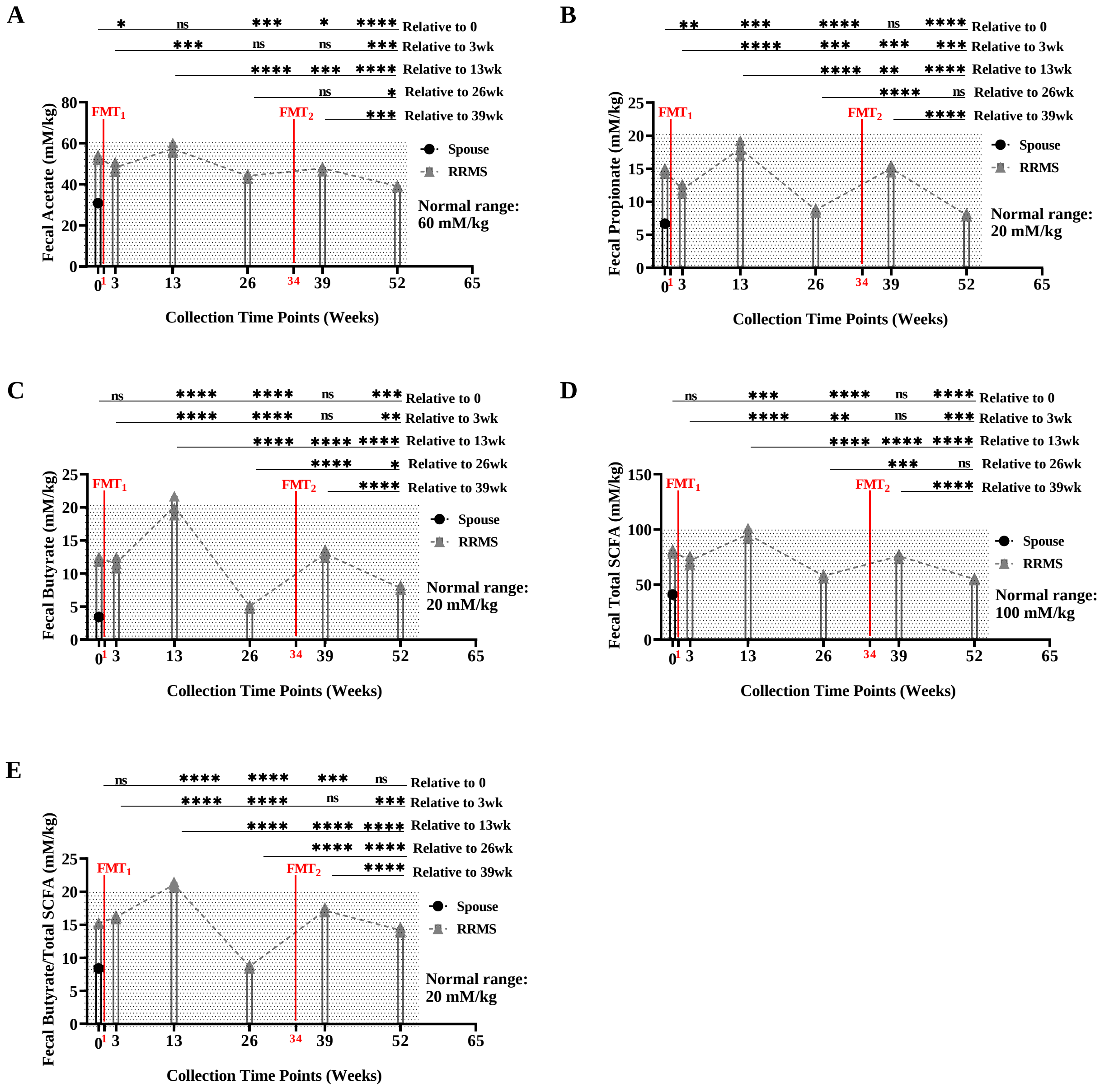


**Supplementary Figure 9.** Fecal targeted short-chain-fatty-acid metabolite's concentration changes over time. Graphs depict (A) acetate (mM/kg); (B) propionate (mM/kg); (C) butyrate (mM/kg); (D) total SCFA (mM/kg); and (E) total Butyrate-to-total SCFA ratio (mM/kg), in the RRMS subject. RRMS subject's Weeks 0, 3, 13, 26, 39, 52 were examined across six time points. The first FMT ($FMT_1$) was between 0 and 3 weeks; second FMT ($FMT_2$) was between 26 and 39 weeks. Post-hoc test of Bonferroni adjusted p-values: ns = no significance, *<0.05, **<0.01, ***<0.001, ****<0.0001.

**Longitudinal Assessment of Serum Neuronal and Inflammatory Biomarkers**

**Supplementary Table 10.** Measurements of serum biomarker changes over time for Brain-derived neurotrophic factor (BDNF) (ng/ml), Interleukin-6 (IL-6) (pg/ml), Interleukin-8 (IL-8) (pg/ml), and Tumor necrosis factor alpha (TNF-α) (pg/ml), in the RRMS FMT subject.

| Multiple Comparison Test | BDNF | IL-6 | IL-8 | TNF-α |
|---|---|---|---|---|
| F (Treatment) & *P*-Value comparisons between baseline and weeks after FMT | **$F_{(5,10)}$ = 54.21 <0.0001** | **$F_{(5,10)}$ = 136.7 <0.0001** | **$F_{(5,10)}$ = 154.5 <0.0001** | **$F_{(5,10)}$ = 88.81 <0.0001** |
| *Bonferroni Post-hoc Test P-Values and Week Mean Average Comparisons in Brackets* | | | | |
| 0wk vs 3wk | **<0.0001 [2.39, 27.60]** | **0.0002 [0.95, 1.71]** | **0.0023 [8.26, 10.25]** | **<0.0001 [0.18, 0.82]** |
| 0wk vs 13wk | **<0.0001 [2.39, 13.09]** | **0.0117 [0.95, 1.33]** | **<0.0001 [8.26, 12.97]** | **0.0023 [0.18, 0.32]** |
| 0wk vs 26wk | **0.0002 [2.39, 10.56]** | **0.0008 [0.95, 1.57]** | **<0.0001 [8.26, 30.77]** | **<0.0001 [0.18, 0.70]** |
| 0wk vs 39wk | **<0.0001 [2.39, 27.31]** | **0.0075 [0.95, 1.37]** | **<0.0001 [8.26, 13.51]** | **<0.0001 [0.18, 0.77]** |
| 0wk vs. 52wk | **<0.0001 [2.39, 19.26]** | **0.0001 [0.95, 1.79]** | **0.0267 [8.26, 6.98]** | **<0.0001 [0.18, 0.62]** |
| 0wk vs. 65wk | **<0.0001 [2.39, 32.12]** | >0.9999 [0.95, 1.05] | **0.0012 [8.26, 6.02]** | **<0.0001 [0.18, 0.58]** |
| 3wk vs. 13wk | **<0.0001 [27.60, 13.09]** | **0.0126 [1.71, 1.33]** | **0.0004 [10.25, 12.97]** | **<0.0001 [0.82, 0.32]** |
| 3wk vs. 26wk | **<0.0001 [27.60, 10.56]** | >0.9999 [1.71, 1.57] | **<0.0001 [10.25, 30.77]** | **0.0065 [0.82, 0.70]** |
| 3wk vs. 39wk | >0.9999 [27.60, 27.31] | **0.0202 [1.71, 1.37]** | **0.0001 [10.25, 13.51]** | 0.3522 [0.82, 0.77] |
| 3wk vs. 52wk | **0.0001 [27.60, 19.26]** | >0.9999 [1.71, 1.79] | **0.0001 [10.25, 6.98]** | **0.0003 [0.82, 0.62]** |
| 3wk vs. 65wk | **0.0050 [27.60, 32.12]** | **0.0006 [1.71, 1.05]** | **<0.0001 [10.25, 6.02]** | **0.0001 [0.82, 0.58]** |
| 13wk vs. 26wk | 0.0988 [13.09, 10.56] | 0.1318 [1.33, 1.57] | **<0.0001 [12.97, 30.77]** | **<0.0001 [0.32, 0.70]** |
| 13wk vs. 39wk | **<0.0001 [13.09, 27.31]** | >0.9999 [1.33, 1.37] | >0.9999 [12.97, 13.51] | **<0.0001 [0.32, 0.77]** |
| 13wk vs. 52wk | **0.0009 [13.09, 19.26]** | **0.0047 [1.33, 1.79]** | **<0.0001 [12.97, 6.98]** | **<0.0001 [0.32, 0.62]** |
| 13wk vs. 65wk | **<0.0001 [13.09, 32.12]** | 0.0560 [1.33, 1.05] | **<0.0001 [12.97, 6.02]** | **<0.0001 [0.32, 0.58]** |
| 26wk vs. 39wk | **<0.0001 [10.56, 27.31]** | 0.2543 [1.57, 1.37] | **<0.0001 [30.77, 13.51]** | 0.1296 [0.70, 0.77] |
| 26wk vs. 52wk | **0.0001 [10.56, 19.26]** | 0.2025 [1.57, 1.79] | **<0.0001 [30.77, 6.98]** | **0.0441 [0.70, 0.62]** |
| 26wk vs. 65wk | **<0.0001 [10.56, 32.12]** | **0.0022 [1.57, 1.05]** | **<0.0001 [30.77, 6.02]** | **0.0070 [0.70, 0.58]** |
| 39wk vs. 52wk | **0.0002 [27.31, 19.26]** | **0.0072 [1.37, 1.79]** | **<0.0001 [13.51, 6.98]** | **0.0019 [0.77, 0.62]** |
| 39wk vs. 65wk | **0.0035 [27.31, 32.12]** | **0.0324 [1.37, 1.05]** | **<0.0001 [13.51, 6.02]** | **0.0006 [0.77, 0.58]** |
| 52wk vs. 65wk | **<0.0001** | **0.0003** | 0.1072 | >0.9999 |

| | | | | |
|---|---|---|---|---|
| | **[19.26, 32.12]** | **[1.79, 1.05]** | [6.98, 6.02] | [0.62, 0.58] |

Parametric repeated measures 1-way ANOVA, based on Shapiro Wilk normality test of residuals and Levene homogeneity of variance, provided a p-value for each test. Post-hoc test of Bonferroni provided an adjusted p-value, along with each collection visit's mean average comparison in brackets. Bold font indicates statistically significant results. A total of 2 measurements per visit. Visit numbers: (0 = baseline; 3 = 3 weeks; 13 = 13 weeks; 26 = 26 weeks; 39 = 39 weeks; 52 = 52 weeks; 65 = 65 weeks). ELISA (enzyme-linked immunosorbent assay).

***Please note: IL-17 was measured, but all collection time points were undetectable (<0.190 pg/ml).***

## Longitudinal Assessment of the Gait Metrics

**Supplementary Table 11.** Summary of statistical results during gait metrics across time in the RRMS FMT subject.

| Multiple Comparison Test | Stride Time (s) | Stride Distance (m) | Cadence (steps/min) | Step Width (m) | Avg. Pelvis Forward Velocity (m/s) | Pelvis Smoothness |
|---|---|---|---|---|---|---|
| *P-Value* comparison between baseline and weeks after FMT | **<0.0001*** | **<0.0001*** | **<0.0001*** | **0.0013** | **<0.0001** | **<0.0001** |
| *Bonferroni Post-hoc Test P-Values and Week Mean Average Comparisons in Brackets* | | | | | | |
| 0wk vs 3wk | **0.0094 [1.24, 1.16]** | **0.0002 [1.23, 1.46]** | **0.0047 [48.82, 52.02]** | 0.1197 [0.19, 0.21] | **<0.0001 [1.31, 1.69]** | >0.9999 [0.10, 0.11] |
| 0wk vs 13wk | 0.5597 [1.24, 1.19] | **0.0179 [1.23, 1.43]** | 0.5597 [48.72, 50.69] | **0.0016 [0.19, 0.22]** | **<0.0001 [1.31, 1.55]** | >0.9999 [0.10, 0.11] |
| 0wk vs. 52wk | **<0.0001 [1.24, 1.12]** | **<0.0001 [1.23, 1.53]** | **<0.0001 [48.72, 53.99]** | >0.9999 [0.19, 0.20] | **<0.0001 [1.31, 1.82]** | **<0.0001 [0.10, 0.29]** |
| 3wk vs. 13wk | 0.8258 [1.16, 1.19] | >0.9999 [1.46, 1.43] | 0.5597 [52.02, 50.69] | 0.8054 [0.21, 0.22] | **0.0016 [1.69, 1.55]** | >0.9999 [0.11, 0.11] |
| 3wk vs. 52wk | 0.7280 [1.16, 1.12] | 0.0589 [1.46, 1.53] | 0.9335 [52.02, 53.99] | 0.6921 [0.21, 0.20] | **0.0036 [1.69, 1.82]** | **<0.0001 [0.11, 0.29]** |
| 13wk vs. 52wk | **0.0145 [1.19, 1.12]** | **0.0008 [1.43, 1.53]** | **0.0117 [50.69, 53.99]** | **0.0177 [0.22, 0.20]** | **<0.0001 [1.55, 1.82]** | **<0.0001 [0.11, 0.29]** |

Parametric repeated measures 1-way ANOVA or non-parametric Friedman's test (*), based on Shapiro Wilk normality test of residuals and Levene homogeneity of variance, provided a p-value for each test. Post-hoc test of Bonferroni (parametric) or Dunn's multiple comparison test (non-parametric) provided an adjusted p-value, along with each visit time's mean average comparison in brackets. Bold font indicates statistically significant results. A total of 18 gait tasks measured per visit. Week Collection: (0 = baseline; 3 = 3 weeks; 13 = 13 weeks; 52 = 52 weeks).

**"Side Gaze Gait" and "Alternating Gaze Gait" Tasks Results Across Lab Visits.**

During the side gaze gait and alternating gaze gait tasks, statistically significantly differences in gait metrics were generally found between week 0 and week 52, except in stride time and cadence (**Tables S12, S13, Figure S10A-F**, and **Figure S11A-F**). Note, these comparisons were performed using only six strides during each condition (i.e., six data points in each gait task group).

**Supplementary Table 12.** Summary of statistical results during side gaze gait tasks across time in the RRMS FMT subject.

| Multiple Comparison Test | Stride Time (s) | Stride Distance (m) | Cadence (steps/min) | Step Width (m) | Avg. Pelvis Forward Velocity (m/s) | Pelvis Smoothness |
|---|---|---|---|---|---|---|
| *P-Value* Comparison between baseline and weeks after FMT | 0.0686 | **<0.0001*** | 0.0559 | **<0.0001** | **<0.0001** | **<0.0001** |
| *Bonferroni Post-hoc Test P-Values and Week Mean Average Comparisons in Brackets* | | | | | | |
| 0wk vs 3wk | >0.9999 [1.23, 1.17] | 0.2650 [1.17, 1.42] | >0.9999 [49.10, 51.35] | **<0.0001 [0.31, 0.21]** | **<0.0001 [1.26, 1.59]** | >0.9999 [0.14, 0.12] |
| 0wk vs 13wk | >0.9999 [1.23, 1.19] | 0.2650 [1.17, 1.41] | >0.9999 [49.10, 50.4] | **<0.0001 [0.31, 0.19]** | **0.0002 [1.26, 1.57]** | >0.9999 [0.14, 0.11] |
| 0wk vs. 52wk | 0.3807 [1.23, 1.11] | **0.0003 [1.17, 1.52]** | 0.3503 [49.10, 54.35] | **<0.0001 [0.31, 0.19]** | **<0.0001 [1.26, 1.80]** | **<0.0001 [0.14, 0.29]** |
| 3wk vs. 13wk | 0.5994 [1.17, 1.19] | >0.9999 [1.42, 1.41] | 0.5984 [51.35, 50.40] | >0.9999 [0.21, 0.19] | >0.9999 [1.59, 1.57] | >0.9999 [0.12, 0.11] |
| 3wk vs. 52wk | 0.3005 [1.17, 1.11] | 0.2650 [1.42, 1.52] | 0.2718 [51.35, 54.35] | >0.9999 [0.21, 0.19] | **0.0080 [1.59, 1.80]** | **<0.0001 [0.12, 0.29]** |
| 13wk vs. 52wk | 0.0718 [1.19, 1.11] | 0.2650 [1.41, 1.52] | 0.0867 [50.40, 54.35] | >0.9999 [0.19, 0.19] | **0.0033 [1.57, 1.80]** | **<0.0001 [0.11, 0.29]** |

Parametric repeated measures 1-way ANOVA or non-parametric Friedman's test (*), based on Shapiro Wilk normality test of residuals and Levene homogeneity of variance, provided a p-value for each test. Post-hoc test of Bonferroni (parametric) or Dunn's multiple comparison test (non-parametric) provided an adjusted p-value, along with each visit time's mean average comparison in brackets. Bold font indicates statistically significant results. A total of 6 gait tasks measured per visit. Week Collection: (0 = baseline; 3 = 3 weeks; 13 = 13 weeks; 52 = 52 weeks).

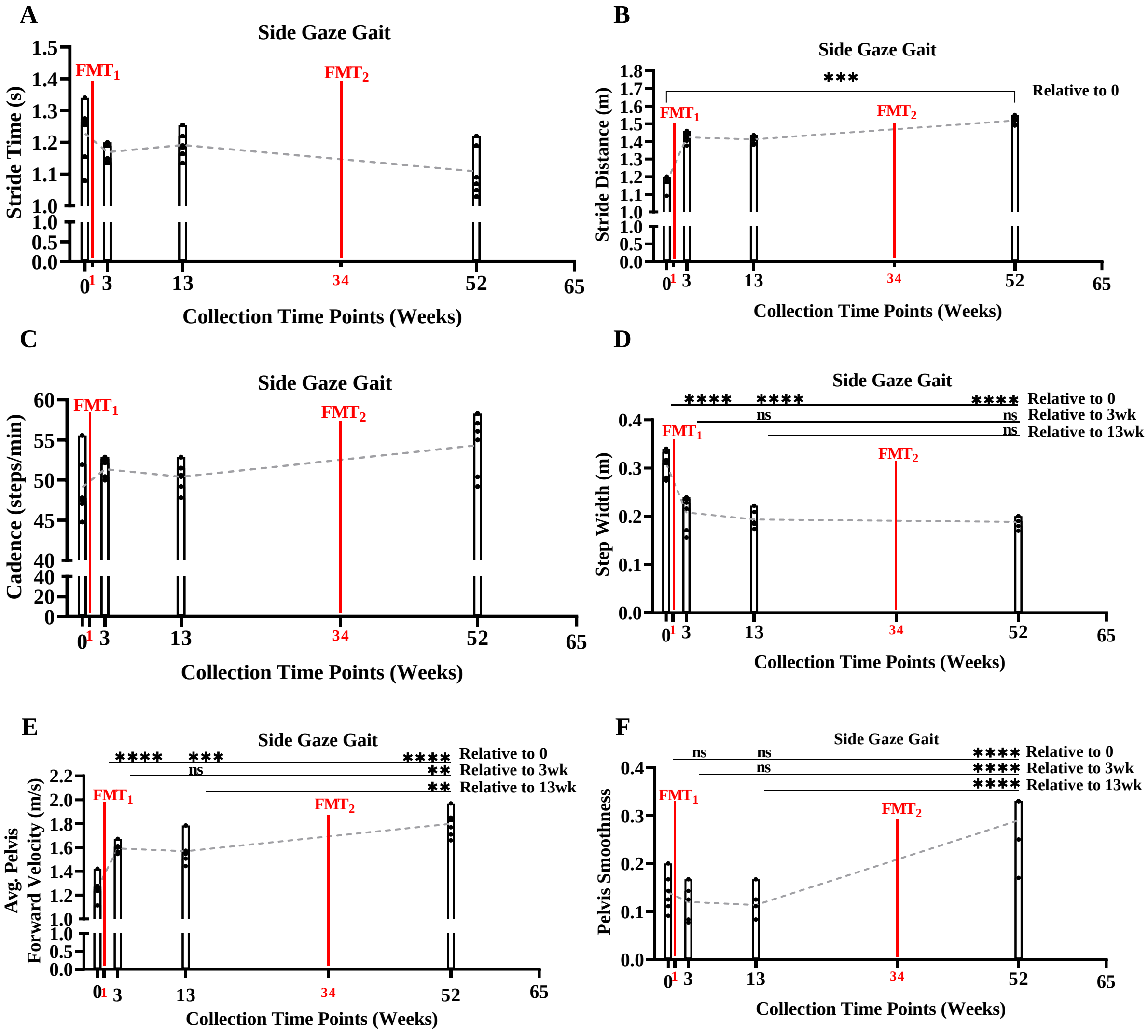


**Supplementary Figure 10.** Measurement of "side" gaze gait tasks changes over time. Graphs depict (A) stride time; (B) stride distance; (C) step width; (D) cadence; (E) average forward velocity; and (F) pelvis smoothness in the RRMS subject. RRMS subject's Weeks 0, 3, 13, 52 were examined across four time points. Directional mean trend dotted line shown across collection time points and FMT weeks. Post-hoc test of Bonferroni adjusted p-values: ns = no significance, *<0.05, **<0.01, ***<0.001, ****<0.0001.

**Supplementary Table 13.** Summary of statistical results during alternating gaze gait tasks across time in the RRMS FMT subject.

| Multiple Comparison Test | Stride Time (s) | Stride Distance (m) | Cadence (steps/min) | Step Width (m) | Avg. Pelvis Forward Velocity (m/s) | Pelvis Smoothness |
|---|---|---|---|---|---|---|
| *P-Value* comparison between baseline and weeks after FMT | 0.5120* | **<0.0001** | 0.5120* | **<0.0001** | **0.0005** | **0.0002** |
| *Bonferroni Post-hoc Test P-Values and Week Mean Average Comparisons in Brackets* | | | | | | |
| 0wk vs 3wk | >0.9999 [1.17, 1.20] | **0.0006 [1.18, 1.31]** | >0.9999 [51.35, 50.27] | **0.0002 [0.32, 0.21]** | >0.9999 [1.35, 1.41] | >0.9999 [0.09, 0.11] |
| 0wk vs 13wk | >0.9999 [1.17, 1.19] | **0.0001 [1.18, 1.33]** | >0.9999 [51.35, 50.58] | **0.0013 [0.32, 0.23]** | 0.1390 [1.35, 1.51] | >0.9999 [0.09, 0.14] |
| 0wk vs. 52wk | >0.9999 [1.17, 1.13] | **<0.0001 [1.18, 1.45]** | >0.9999 [51.35, 53.23] | **0.0002 [0.32, 0.21]** | **0.0005 [1.35, 1.69]** | **0.0064 [0.09, 0.28]** |
| 3wk vs. 13wk | >0.9999 [1.20, 1.19] | >0.9999 [1.31, 1.33] | >0.9999 [50.27, 50.58] | >0.9999 [0.21, 0.23] | 0.8031 [1.41, 1.51] | >0.9999 [0.11, 0.14] |
| 3wk vs. 52wk | >0.9999 [1.20, 1.13] | **0.0003 [1.31, 1.45]** | >0.9999 [50.27, 53.23] | >0.9999 [0.21, 0.21] | **0.0033 [1.41, 1.69]** | **0.0061 [0.11, 0.28]** |
| 13wk vs. 52wk | >0.9999 [1.19, 1.13] | **0.0012 [1.33, 1.45]** | >0.9999 [50.58, 53.23] | >0.9999 [0.23, 0.21] | 0.0829 [1.51, 1.69] | **0.0037 [0.14, 0.28]** |
| Parametric repeated measures 1-way ANOVA or non-parametric Friedman's test (*), based on Shapiro Wilk normality test of residuals and Levene homogeneity of variance, provided a p-value for each test. Post-hoc test of Bonferroni (parametric) or Dunn's multiple comparison test (non-parametric) provided an adjusted p-value, along with each visit time's mean average comparison in brackets. Bold font indicates statistically significant results. A total of 6 gait tasks measured per visit. Week Collection: (0 = baseline; 3 = 3 weeks; 13 = 13 weeks; 52 = 52 weeks). | | | | | | |

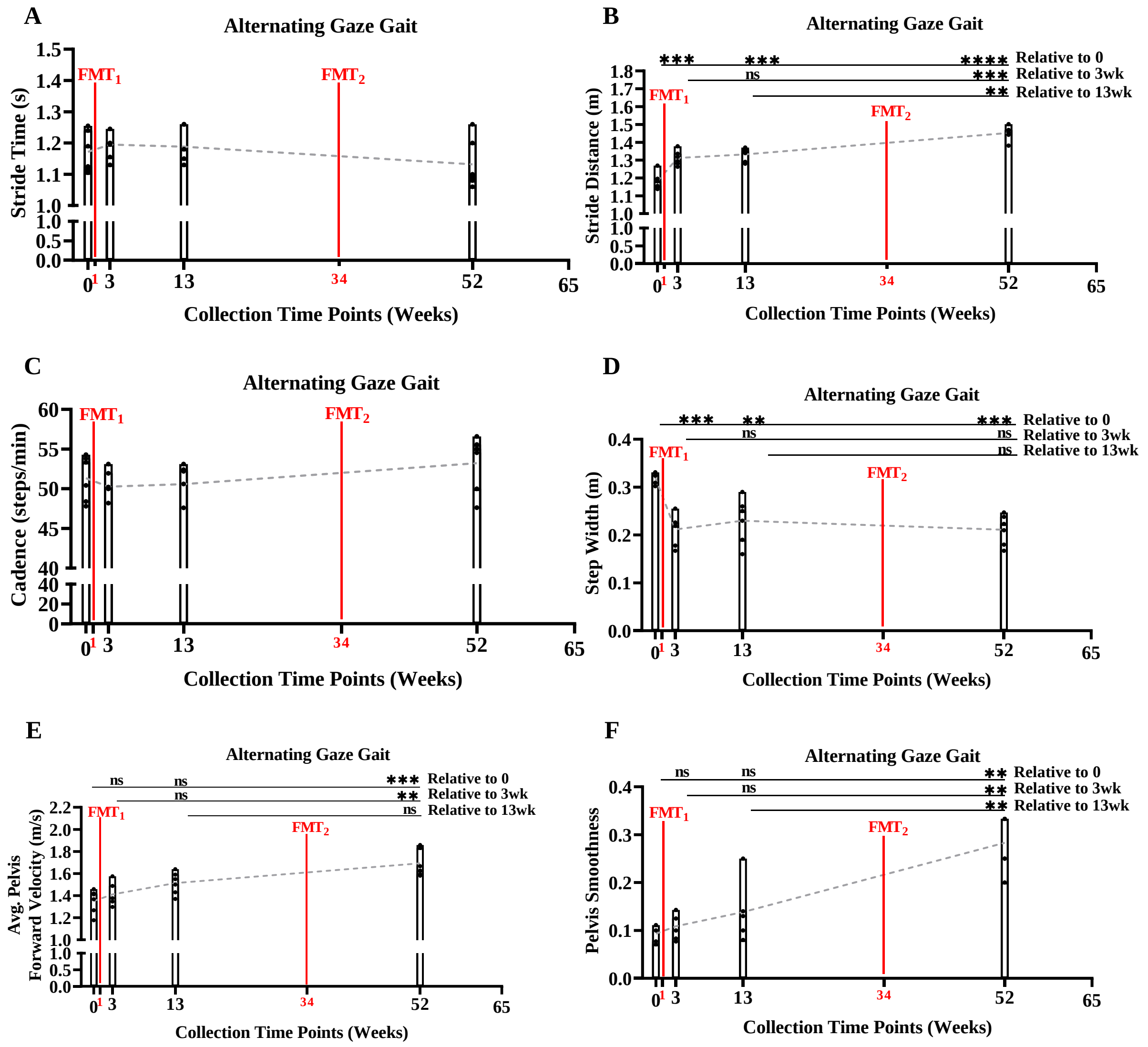


**Supplementary Figure 11.** Measurement of "alternating" gaze gait tasks changes over time. Graphs show (A) stride time; (B) stride distance; (C) step width; (D) cadence; (E) average forward velocity; and (F) pelvis smoothness in the RRMS subject. RRMS subject's Weeks 0, 3, 13, 52 were examined across four time points. Directional mean trend dotted line shown across collection time points and FMT weeks. Post-hoc test of Bonferroni adjusted p-values: ns = no significance, *<0.05, **<0.01, ***<0.001, ****<0.0001.

## Within Lab Visit Results Across Gait Tasks.

Within each week collection time point, there were few statistically significant differences between the different types of gait tasks, except in stride distance (**Table S14, Figure S12A-F**). During week 0 (baseline), there was significantly smaller step width used during the gait task, when compared to both the side gaze gait and alternating gaze gait tasks that was not present during weeks 3, 13 and 52 (**Figure S12D**). Stride distance during weeks 3, 13, and 52 was significantly smaller

during the alternating gaze gait task, when compared to both the gait or side gaze gait (**Figure S12B**). During week 3, stride distance was significantly greater during gait vs. the alternating gaze gait task (**Figure S12B**). Similarly, during week 3, average pelvis forward speed was significantly greater during gait against the alternating gaze gait task (**Figure S12E**). During week 13, stride distance was significantly greater during side gaze gait compared to alternating gaze gait (**Figure S12B**). Finally, during week 52, stride distance was significantly smaller during alternating gaze gait compared to both side gaze gait and gait tasks (**Figure S12B**).

**Supplementary Table 14.** Summary of statistical results during each week's collection time point across gait tasks, in the RRMS FMT subject.

| Metric | Kruskal-Wallis | | Post-hoc Mean Rank Tests | | |
|---|---|---|---|---|---|
| | $X^2$ | *P*-Value | Gait vs. Side | Gait vs. Alternating | Side vs. Alternating |
| Lab Week 0 | | | | | |
| Stride Time (s) | 3.55 | 0.170 | 1.00 [-6.86, 7.86] | 0.26 [-2.11, 12.61] | 0.37 [-2.61, 12.11] |
| Stride Distance (m) | 6.88 | **0.030** | 0.07 [-0.38, 14.38] | 0.07 [-0.38, 14.38] | 1.00 [-7.38, 7.38] |
| Cadence (steps/min) | 3.55 | 0.170 | 1.00 [-7.86, 6.86] | 0.26 [-12.61, 2.11] | 0.37 [-12.11, 2.61] |
| Step Width (m) | 11.42 | **<0.001** | **0.01 [-16.05, -1.29]** | **0.01 [-16.71, -1.95]** | 1.00 [-8.05, 6.71] |
| Avg. Pelvis Forward Velocity (m/s) | 2.26 | 0.320 | 0.64 [-3.55, 11.21] | 1.00 [-7.71, 7.05] | 0.53 [-11.55, 3.21] |
| Pelvis Smoothness | 5.96 | **0.049** | 0.12 [-13.4, 1.07] | 1.00 [-6.82, 7.65] | 0.09 [-0.65, 13.82] |
| Lab Week 3 | | | | | |
| Stride Time (s) | 3.53 | 0.17 | 0.87 [-10.6, 4.1] | 0.18 [-13.1, 1.6] | 1.00 [-9.85, 4.85] |
| Stride Distance (m) | 11.59 | **<0.001** | 1.00 [-4.55, 10.21] | **<0.001 [2.79, 17.55]** | 0.05 [-0.05, 14.71] |
| Cadence (steps/min) | 3.53 | 0.17 | 0.87 [-4.1, 10.6] | 0.18 [-1.6, 13.1] | 1.00 [-4.85, 9.85] |
| Step Width (m) | 0.15 | 0.93 | 1.00 [-8.55, 6.21] | 1.00 [-8.21, 6.55] | 1.00 [-7.05, 7.71] |
| Avg. Pelvis Forward Velocity (m/s) | 11.68 | **<0.001** | 0.43 [-2.88, 11.88] | **<0.001 [3.12, 17.88]** | 0.15 [-1.38, 13.38] |
| Pelvis Smoothness | 0.34 | 0.85 | 1.00 [-8.26, 6.26] | 1.00 [-6.51, 8.01] | 1.00 [-5.51, 9.01] |
| Lab Week 13 | | | | | |
| Stride Time (s) | 0.15 | 0.93 | 1.00 [-7.01, 7.67] | 1.00 -6.17, 8.51] | 1.00 [-6.51, 8.17] |
| Stride Distance (m) | 9.40 | **0.01** | 1.00 [-9.38, 5.38] | 0.07 [-0.38, 14.38] | **0.01 [1.62, 16.38]** |
| Cadence (steps/min) | 0.15 | 0.93 | 1.00 [-7.67, 7.01] | 1.00 [-8.51, 6.17] | 1.00 [-8.17, 6.51] |
| Step Width (m) | 4.25 | 0.12 | 0.22 [-1.88, 12.88] | 1.00 [-7.38, 7.38] | 0.22 [-12.88, |
| Avg. Pelvis Forward Velocity (m/s) | 0.74 | 0.69 | 1.00 [-9.88, 4.88] | 1.00 [-7.88, 6.88] | 1.00 [-5.38, 9.38] |
| Pelvis Smoothness | 1.58 | 0.45 | 1.00 [-9.48, 5.14] | 0.63 [-11.14, 3.48] | 1.00 [-8.98, 5.64] |
| Lab Week 52 | | | | | |
| Stride Time (s) | 1.92 | 0.38 | 0.53 | 1.00 | 1.00 |

| | | | | | |
|---|---|---|---|---|---|
| | | | [-3.19, 11.52] | [-6.02, 8.69] | [-10.19, 4.52] |
| Stride Distance (m) | 10.19 | **0.01** | 1.00<br>[-6.71, 8.05] | **0.01**<br>**[1.45, 16.21]** | **0.02**<br>**[0.79, 15.55]** |
| Cadence (steps/min) | 1.92 | 0.38 | 0.53<br>[-11.52, 3.19] | 1.00<br>[-8.69, 6.02] | 1.00<br>[-4.52, 10.19] |
| Step Width (m) | 3.87 | 0.14 | 0.25<br>[-2.05, 12.71] | 1.00<br>[-7.21, 7.55] | 0.28<br>[-12.55, 2.21] |
| Avg. Pelvis Forward Velocity (m/s) | 2.61 | 0.27 | 1.00<br>[-6.55, 8.21] | 0.39<br>[-2.71, 12.05] | 0.64,<br>[-3.55, 11.21] |
| Pelvis Smoothness | 0.24 | 0.89 | 1.00<br>[-6.19, 6.69] | 1.00<br>[-5.19, 7.69] | 1.00<br>[-5.44, 7.44] |

Omnibus Kruskal-Wallis provided a chi-square ($x^2$) and p-value for each test. The Dunn's multiple comparison post-hoc mean rank tests provided adjusted p-values, and confidence intervals in square brackets. Bold font indicates statistically significant results. Week Collection: (0 = baseline; 3 = 3 weeks; 13 = 13 weeks; 52 = 52 weeks).

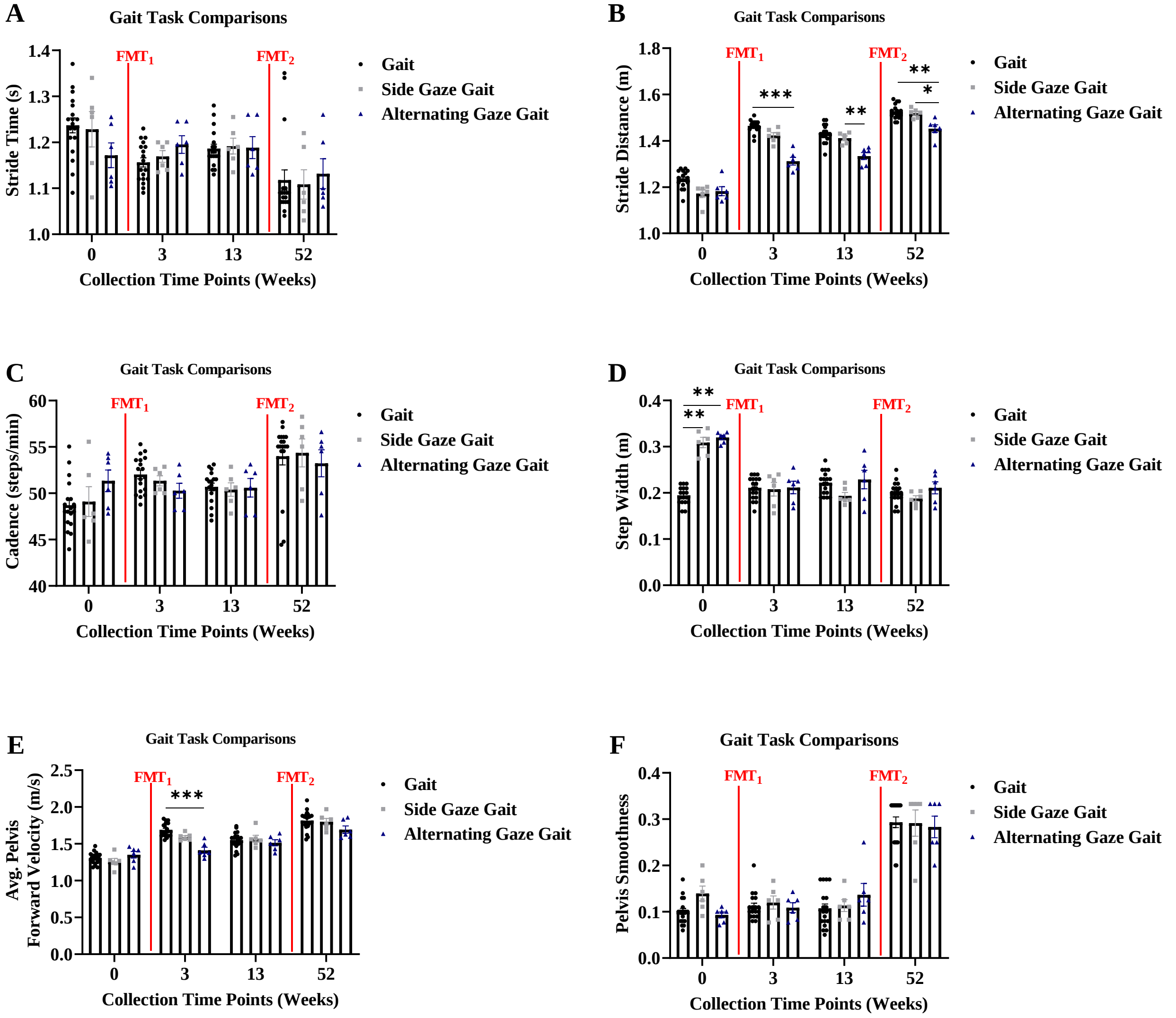


**Supplementary Figure 12.** Gait task comparisons during each collection time point. Graphs illustrate (A) stride time; (B) stride distance; (C) step width; (D) cadence; (E) average forward velocity; and (F) pelvis smoothness in the RRMS subject. RRMS subject's Weeks 0, 3, 13, 52 were examined across four time points. The first FMT ($FMT_1$) was between 0 and 3 weeks; second FMT ($FMT_2$) was between 26 and 39 weeks. Dunn's multiple comparison post-hoc adjusted p-values: *<0.05, **<0.01, ***<0.001.

## Multiple Sclerosis Walking Scale Score Outcomes

**Supplementary Table 15.** The linear regression relationships between the MS Walking Scale Questionnaire variables across six time points in the RRMS FMT subject.

| Walking Score Metric | Slope | $R^2$ value | *P*-value |
|---|---|---|---|
| Limited your ability to walk | -0.024 | 0.63 | 0.058 |
| Limited your ability to run | -0.041 | 0.52 | 0.105 |
| Limited your ability to climb up/down stairs | -0.034 | 0.74 | **0.027** |
| Made standing difficult | -0.019 | 0.60 | 0.070 |
| Limited your balance | -0.022 | 0.77 | **0.022** |
| Limited how far able to walk | -0.032 | 0.78 | **0.019** |
| Increased effort to walk | -0.024 | 0.63 | 0.058 |
| Necessary to use support indoors | 0 | 1 | 1 |
| Necessary to use support outdoors | 0 | 1 | 1 |
| Slowed down walking | -0.043 | 0.65 | 0.053 |
| Affected how smoothly you walked | -0.032 | 0.78 | **0.019** |
| Made you concentrate on your walking | -0.028 | 0.50 | 0.116 |

Linear regression ($R^2$) values are shown. Multiple Sclerosis Walking Scale Questionnaire. Weeks 0, 3, 13, 26, 39, 52 were examined across six time points (n=6). "In the past two weeks, how much has your MS….insert walking variable + score" Scale Range: 1-5 (1 = not all; 2 = a little; 3 = moderately; 4 = quite a lot; 5 = extremely).

## PROMIS-GI Characteristics

**Supplementary Table 16.** Average PROMIS-GI scores for general population and RRMS FMT subject across time.

| PROMIS Variables | Number of Items | General Population Mean (s.d.) | MS Patient All Times Mean (s.d.) | Baseline T-Score (s.d) | 3 Weeks T-Score (s.d) | 13 Weeks T-Score (s.d) | 26 Weeks T-Score (s.d) | 39 Weeks T-Score (s.d) | 52 Weeks T-Score (s.d) |
|---|---|---|---|---|---|---|---|---|---|
| Belly Pain | 6 | 50 (10) | 56.20 (8.20) | 56.20 (8.20) | 56.20 (8.20) | 56.20 (8.20) | 56.20 (8.20) | 56.20 (8.20) | 56.20 (8.20) |
| Constipation | 9 | 50 (10) | 53.98 (1.88) | 53.80 (1.80) | 54.40 (1.90) | 51.50 (2.10) | 56.20 (1.80) | 55.70 (1.80) | 52.30 (1.90) |
| Bowel Incontinence | 4 | 50 (10) | 42 (7.70) | 42 (7.70) | 42 (7.70) | 42 (7.70) | 42 (7.70) | 42 (7.70) | 42 (7.70) |
| Gas & Bloating | 12 | 50 (10) | 56.33 (1.58) | 56.40 (2.00) | 57.60 (2.00) | 56.40 (2.00) | 55.70 (1.20) | 56.10 (1.20) | 55.80 (1.10) |

GI, gastrointestinal; PROMIS, Patient-Reported Outcomes Measurement Information System; s.d. = standard deviation. A score of 50 = mean of general population reference sample. By design, all PROMIS scales are normed to a score of 50 and standard deviation of 10 in the general population using a T-metric. Higher score denotes more GI symptoms. Lower score denotes less GI symptoms.

**Magnetic Resonance Imaging**

Magnetic Resonance Imaging (MRI) (RUMC Neurology) of the brain and spine for lesions were examined near week 0 (baseline; before $FMT_1$) and near week 39 (after $FMT_1$ and $FMT_2$) time point collections, in the RRMS subject. Please note that a follow-up MRI cervical spine did not occur during this study's time frame. The diagnostics imaging impression summaries are stated as follows:

Week 0 MRI Brain: *"Severe progression of supratentorial demyelinating disease with multiple large demyelinating plaques. There are numerous enhancing plaques indicating active demyelination."*

Week 0 MRI Cervical Spine: *"Overall, the burden of enhancing lesions has decreased. However, the lesion at C3 demonstrates enlargement with some swelling and more enhancement. Also, there is mild degenerative spinal stenosis at C6-C7, which is unchanged."*

Week 39 MRI Brain: *"Stable unchanged appearance of the brain when compared to the prior examination. Constellation of findings are consistent with the clinical history of MS. The T-1 weighted plaque burden is mild in degree and the T-2 weighted plaque burden is moderate in degree."*

**Baseline and Longitudinal Assessment of Dietary Outcomes**

**Supplementary Table 17.** The ASA24® number of servings calculated at baseline, in the RRMS FMT subject.

| ASA24® Category | Export Data Measures | Primary Contributors to Energy Intake | Serving Size | Quantity Consumed | Number of Servings |
|---|---|---|---|---|---|
| F_TOTAL | Total intact fruits (whole or cut) and fruit juices (cup eq.) | Grapes | 1/2 cup | 2.13 cups | **4.26** |
| V_TOTAL | Total dark green, red and orange, starchy, and other vegetables; excludes legumes (cup eq.) | Onions, Cucumbers, Tomatoes, Peppers, Dandelion Greens, Carrots | 1/2 cup | 6.25 cups | **12.5** |
| V_LEGUMES | Beans and peas (legumes) computed as vegetables (cup eq.) | Hummus (garbanzo beans) | 1/4 cup | 0.83 cups | **3.32** |
| G_WHOLE | Grains defined as whole grains and contain the entire grain kernel? The bran, germ, and endosperm (oz. eq.) | None | N/A | 0 | **0** |
| PF_MPS_TOTAL | Total of meat, poultry, seafood, organ meat, and cured meat (oz. eq.) | Beef, Pork Tenderloin | 3 oz. | 4.12 ounces | **1.37** |
| PF_NUTSDS | Peanuts, tree nuts, and seeds; excludes coconut (oz. eq.) | Hummus, Tahini | 1 ounce | 3 ounces | **3** |
| As serving size differs based on individual foods within category, servings were determined by looking at foods eaten that day of the first recall (baseline: week 0). | | | | | |

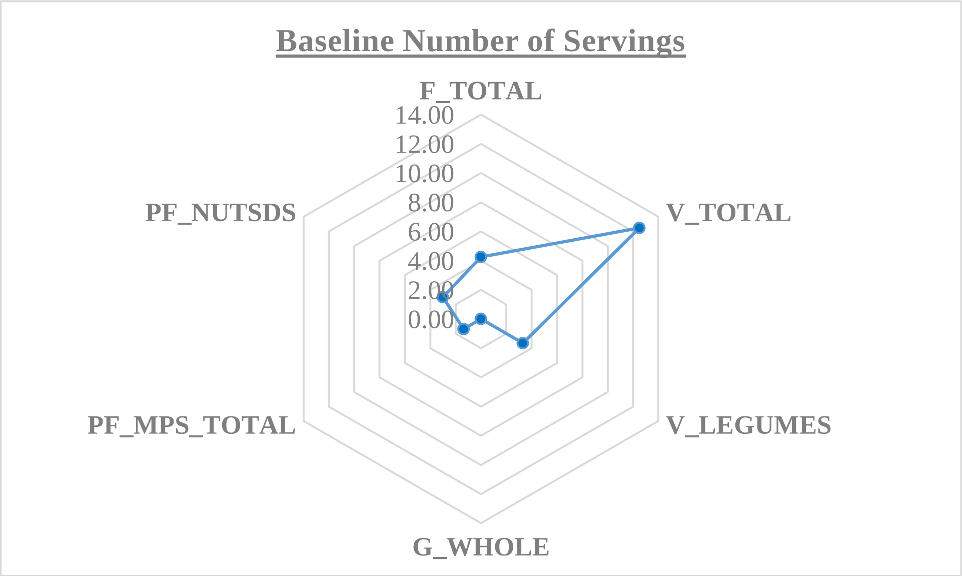


**Supplementary Figure 13.** The ASA24® nutrient category number of servings calculated at baseline (week 0) in the RRMS FMT subject.

**Supplementary Table 18.** The ASA24® nutrient variables, categories, baseline data, and linear regression relationships between ASA24® nutrition variables across six time points in the RRMS FMT subject.

| ASA24® Nutrient Variables | ASA24® Category | Baseline Data | Slope | $R^2$ value | P-value |
|---|---|---|---|---|---|
| Energy (kcal) | KCAL | 3250.76 | -11.44 | 0.20 | 0.371 |
| Protein (g) | PROT | 114.25 | -1.30 | 0.64 | 0.056 |
| Total Fat (g) | TFAT | 145.83 | -0.47 | 0.06 | 0.645 |
| Carbohydrate (g) | CARB | 384.22 | -0.84 | 0.14 | 0.464 |
| Sugars, total (g) | SUGR | 203.06 | -0.55 | 0.13 | 0.485 |
| Fiber, total dietary (g) | FIBE | 33.1 | -0.23 | 0.43 | 0.158 |
| Fatty acids, total saturated (g) | SFAT | 30.27 | -0.05 | 0.02 | 0.813 |
| Total intact fruits (whole or cut) and fruit juices (cup eq.) | F_TOTAL | 2.13 | -0.05 | 0.81 | **0.014** |
| Total dark green, red and orange, starchy, and other vegetables; excludes legumes (cup eq.) | V_TOTAL | 6.25 | -0.07 | 0.56 | 0.088 |
| Dark green vegetables (cup eq.) | V_DRKGR | 0 | 0.01 | 0.21 | 0.367 |
| Beans and peas (legumes) computed as vegetables (cup eq.) | V_LEGUMES | 0.83 | -0.01 | 0.27 | 0.292 |
| Grains defined as whole grains and contain the entire grain kernel? The bran, germ, and endosperm (oz. eq.) | G_WHOLE | 0 | 0.02 | 0.76 | **0.024** |
| Total of meat, poultry, seafood, organ meat, and cured meat (oz. eq.) | PF_MPS_TOTAL | 8.93 | -0.15 | 0.62 | 0.064 |
| Peanuts, tree nuts, and seeds; excludes coconut (oz. eq.) | PF_NUTSDS | 3 | -0.02 | 0.19 | 0.390 |
| Fats naturally present in meat, poultry, eggs, dairy (lard, tallow, butter); hydrogenated/partially hydrogenated oils; shortening, palm, palm kernel, coconut oils; coconut meat, cocoa butter; 50% of fat in stick/tub margarines, margarine spreads (grams) | SOLID_FATS | 22.43 | 0.25 | 0.31 | 0.251 |
| Foods defined as added sugars (tsp. eq.) | ADD_SUGARS | 29.87 | 0.13 | 0.14 | 0.463 |
| Linear regression ($R^2$) values are shown. ASA24®: Automated Self-Administered 24-Hour Dietary Assessment Tool. Weeks 0, 3, 13, 26, 39, 52 were examined across six time points (n=6). | | | | | |

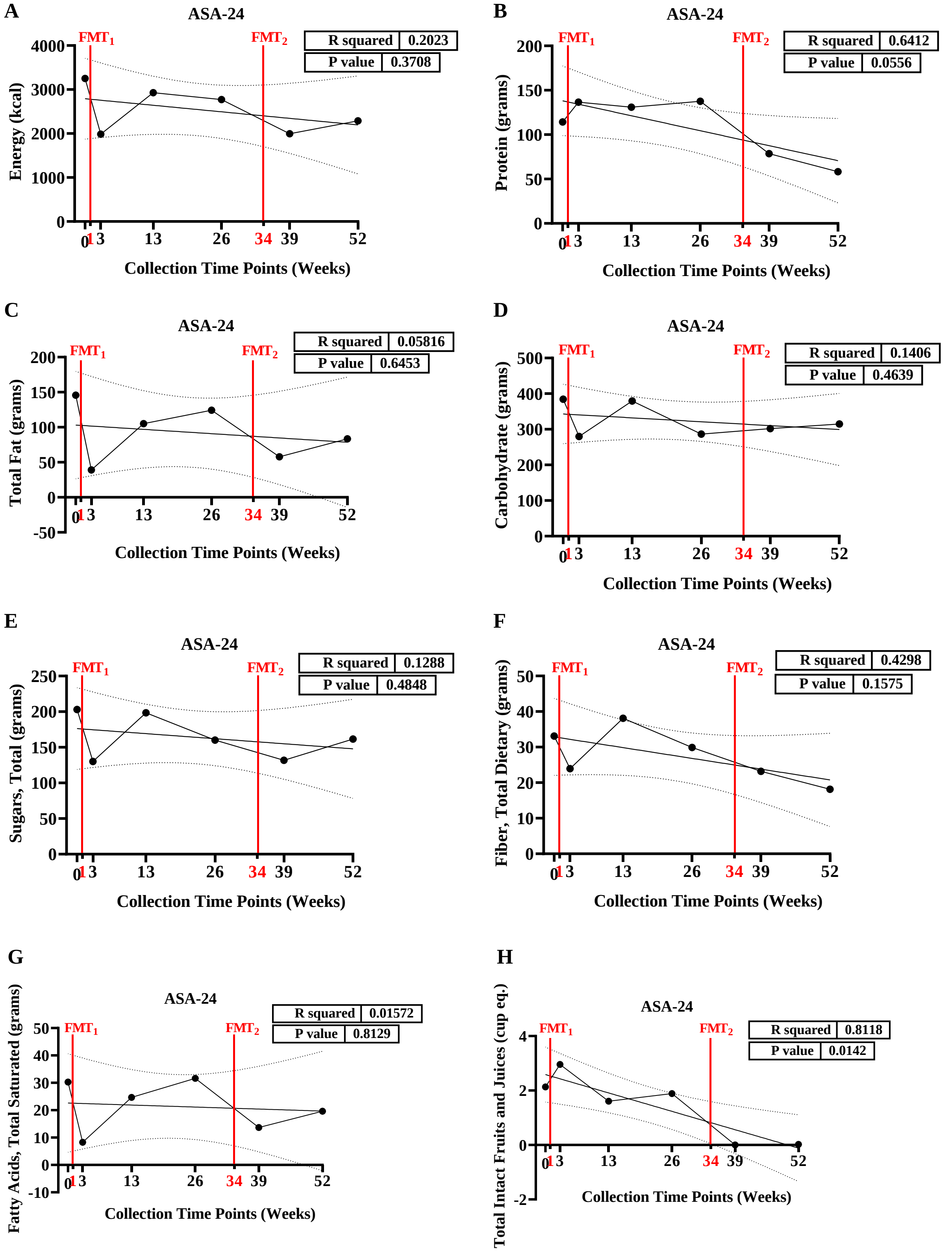
A
ASA-24
FMT1
FMT2
R squared 0.2023
P value 0.3708
Energy (kcal)
4000
3000
2000
1000
0
0 1 3 13 26 34 39 52
Collection Time Points (Weeks)
B
ASA-24
FMT1
FMT2
R squared 0.6412
P value 0.0556
Protein (grams)
200
150
100
50
0
0 1 3 13 26 34 39 52
Collection Time Points (Weeks)
C
ASA-24
FMT1
FMT2
R squared 0.05816
P value 0.6453
Total Fat (grams)
200
150
100
50
0
-50
0 1 3 13 26 34 39 52
Collection Time Points (Weeks)
D
ASA-24
FMT1
FMT2
R squared 0.1406
P value 0.4639
Carbohydrate (grams)
500
400
300
200
100
0
0 1 3 13 26 34 39 52
Collection Time Points (Weeks)
E
ASA-24
FMT1
FMT2
R squared 0.1288
P value 0.4848
Sugars, Total (grams)
250
200
150
100
50
0
0 1 3 13 26 34 39 52
Collection Time Points (Weeks)
F
ASA-24
FMT1
FMT2
R squared 0.4298
P value 0.1575
Fiber, Total Dietary (grams)
50
40
30
20
10
0
0 1 3 13 26 34 39 52
Collection Time Points (Weeks)
G
ASA-24
FMT1
FMT2
R squared 0.01572
P value 0.8129
Fatty Acids, Total Saturated (grams)
50
40
30
20
10
0
-10
0 1 3 13 26 34 39 52
Collection Time Points (Weeks)
H
ASA-24
FMT1
FMT2
R squared 0.8118
P value 0.0142
Total Intact Fruits and Juices (cup eq.)
4
2
0
-2
0 1 3 13 26 34 39 52
Collection Time Points (Weeks)

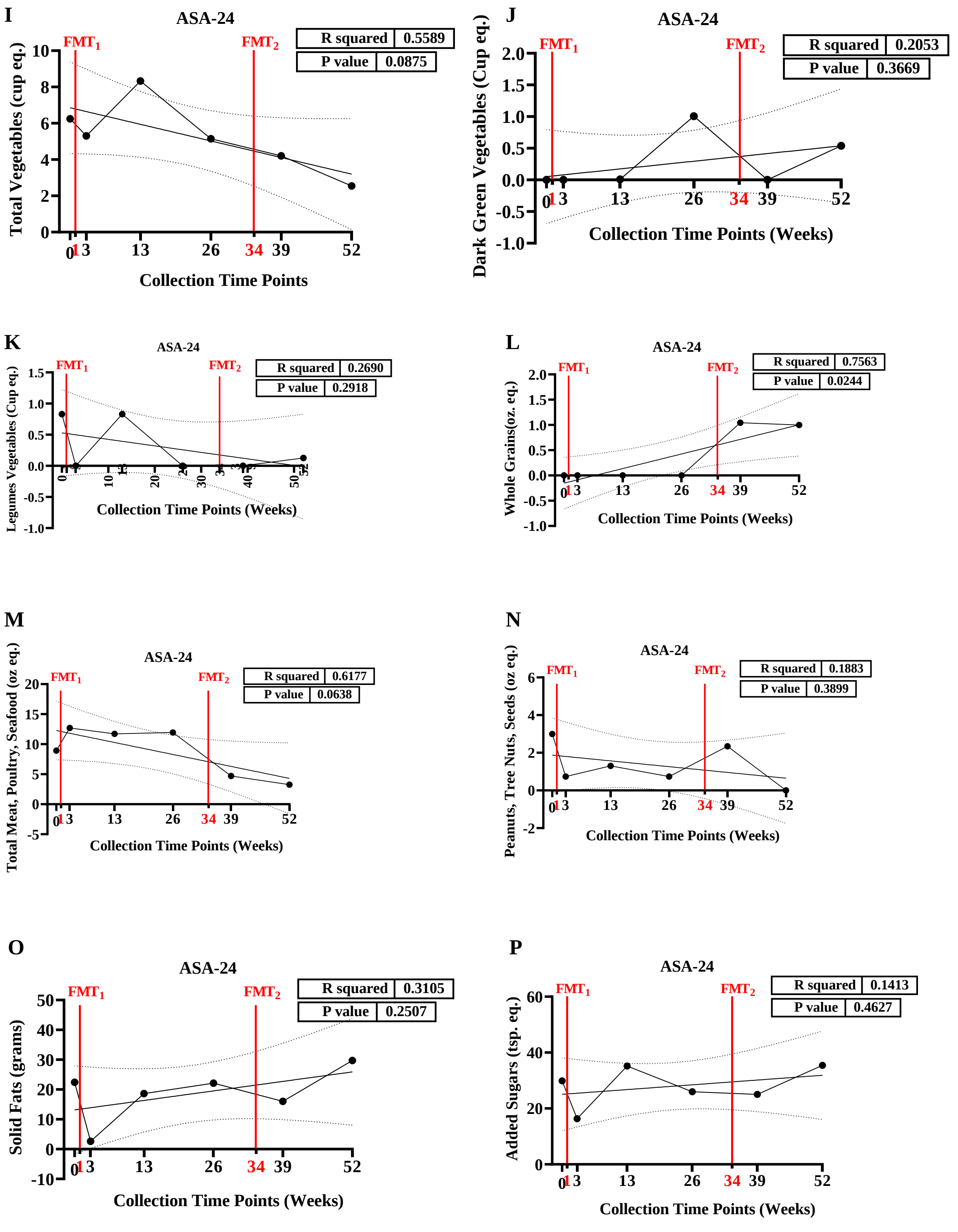


**Supplementary Figure 14.** Linear regression relationships between ASA24® nutrition variables across six time points, in the RRMS FMT subject. RRMS subject's Weeks 0, 3, 13, 26, 39, and 52 were examined across six collection time points. The $R^2$ and P-values are shown within each graph.

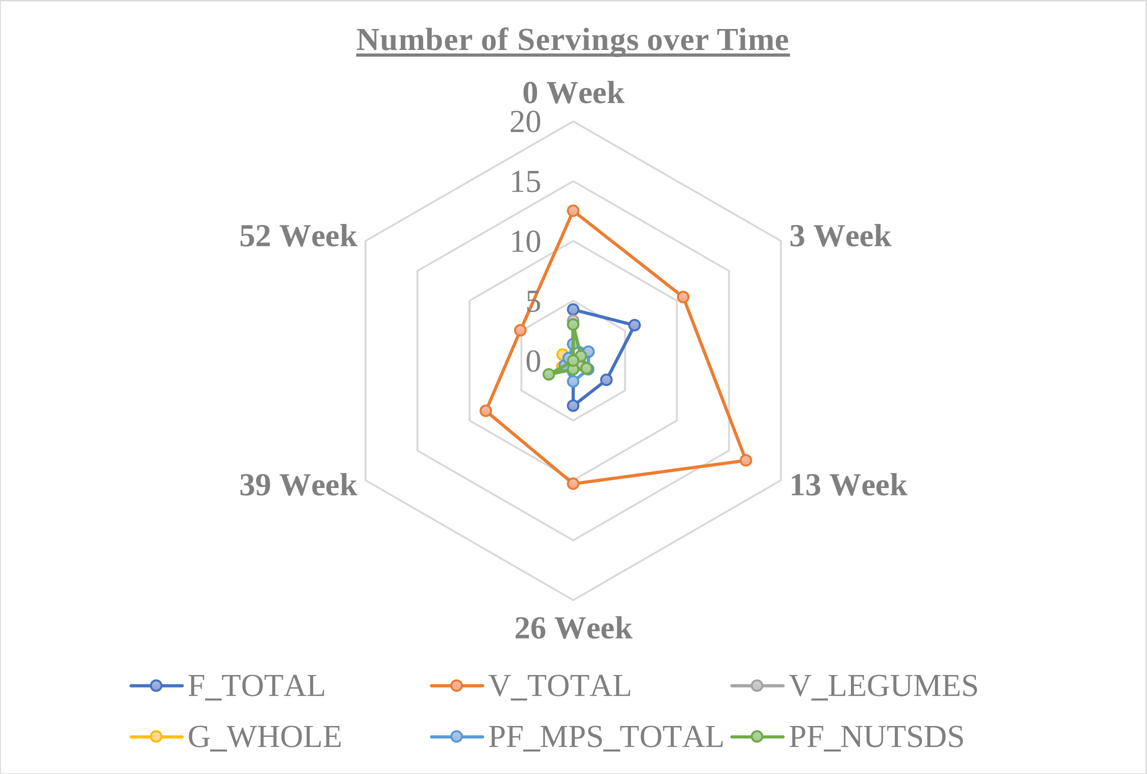


**Supplementary Figure 15.** Number of Servings over time. ASA24® nutrient category number of servings over time calculated at six different collection time points (0, 3, 13, 26, 39, and 52 weeks), in the RRMS FMT subject.

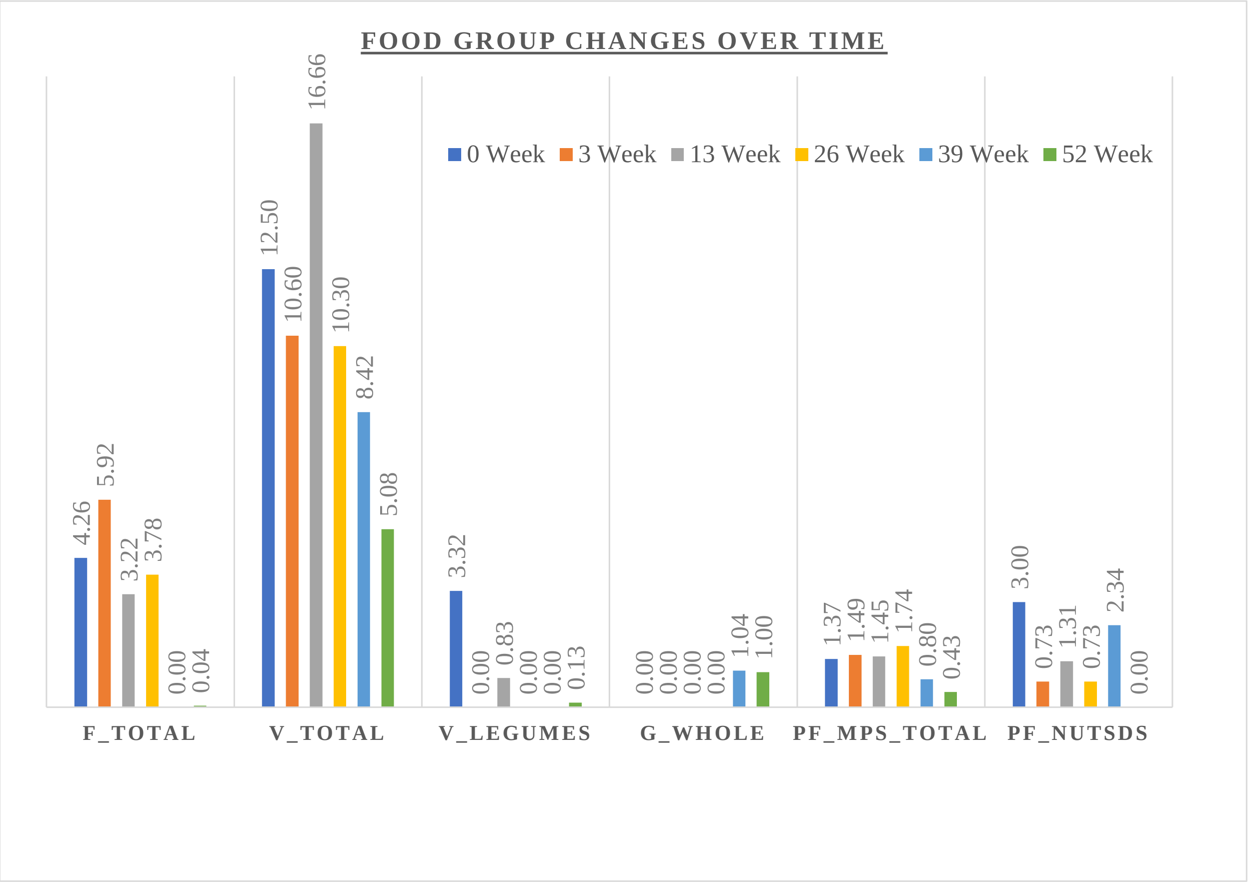


**Supplementary Figure 16.** Food group changes over time. ASA24® nutrient category food group changes over time calculated at six different collection time points (0, 3, 13, 26, 39, and 52 weeks), in the RRMS FMT subject. The total number of servings is shown per time point, within each nutrient category.

**Supplementary Table 19.** The ASA24® nutrient variables (per 1000 kcal), categories, and linear regression relationships between ASA24® nutrition (per 1000 kcal) variables across six time points in the RRMS FMT subject.

| ASA24® Nutrient (per 1000 kcal) Variables | ASA24® Category | Slope | $R^2$ value | *P*-value |
|---|---|---|---|---|
| Energy (kcal) | KCAL | -11.44 | 0.20 | 0.371 |
| Protein (g) 1000 kcal | PROT | -0.42 | 0.35 | 0.220 |
| Total Fat (g) 1000 kcal | TFAT | 0.22 | 0.002 | 0.371 |
| Carbohydrate (g) 1000 kcal | CARB | 0.25 | 0.09 | 0.562 |
| Sugars, total (g) 1000 kcal | SUGR | 0.08 | 0.14 | 0.459 |
| Fiber, total dietary (g) 1000 kcal | FIBE | -0.05 | 0.32 | 0.244 |
| Fatty acids, total saturated (g) 1000 kcal | SFAT | 0.02 | 0.04 | 0.701 |
| Total intact fruits (whole or cut) and fruit juices (cup eq.) 1000 kcal | F_TOTAL | -0.02 | 0.64 | 0.056 |
| Total dark green, red and orange, starchy, and other vegetables; excludes legumes (cup eq.) 1000 kcal | V_TOTAL | -0.02 | 0.47 | 0.134 |
| Dark green vegetables (cup eq.) 1000 kcal | V_DRKGR | 0.004 | 0.27 | 0.292 |
| Beans and peas (legumes) computed as vegetables (cup eq.) 1000 kcal | V_LEGUMES | -0.003 | 0.22 | 0.343 |
| Grains defined as whole grains and contain the entire grain kernel? The bran, germ, and endosperm (oz. eq.) 1000 kcal | G_WHOLE | 0.01 | 0.72 | **0.032** |
| Total of meat, poultry, seafood, organ meat, and cured meat (oz. eq.) 1000 kcal | PF_MPS_TOTAL | -0.06 | 0.45 | 0.146 |
| Peanuts, tree nuts, and seeds; excludes coconut (oz. eq.) 1000 kcal | PF_NUTSDS | -0.005 | 0.06 | 0.643 |
| Fats naturally present in meat, poultry, eggs, dairy (lard, tallow, butter); hydrogenated/partially hydrogenated oils; shortening, palm, palm kernel, coconut oils; coconut meat, cocoa butter; 50% of fat in stick/tub margarines, margarine spreads (grams) 1000 kcal | SOLID_FATS | 0.15 | 0.67 | **0.046** |
| Foods defined as added sugars (tsp. eq.) 1000 kcal | ADD_SUGARS | 0.11 | 0.71 | **0.034** |

Linear regression ($R^2$) values are shown. ASA24®: Automated Self-Administered 24-Hour Dietary Assessment Tool. Weeks 0, 3, 13, 26, 39, 52 were examined across six time points (n=6). Normalize data to energy (kcalories) intake.

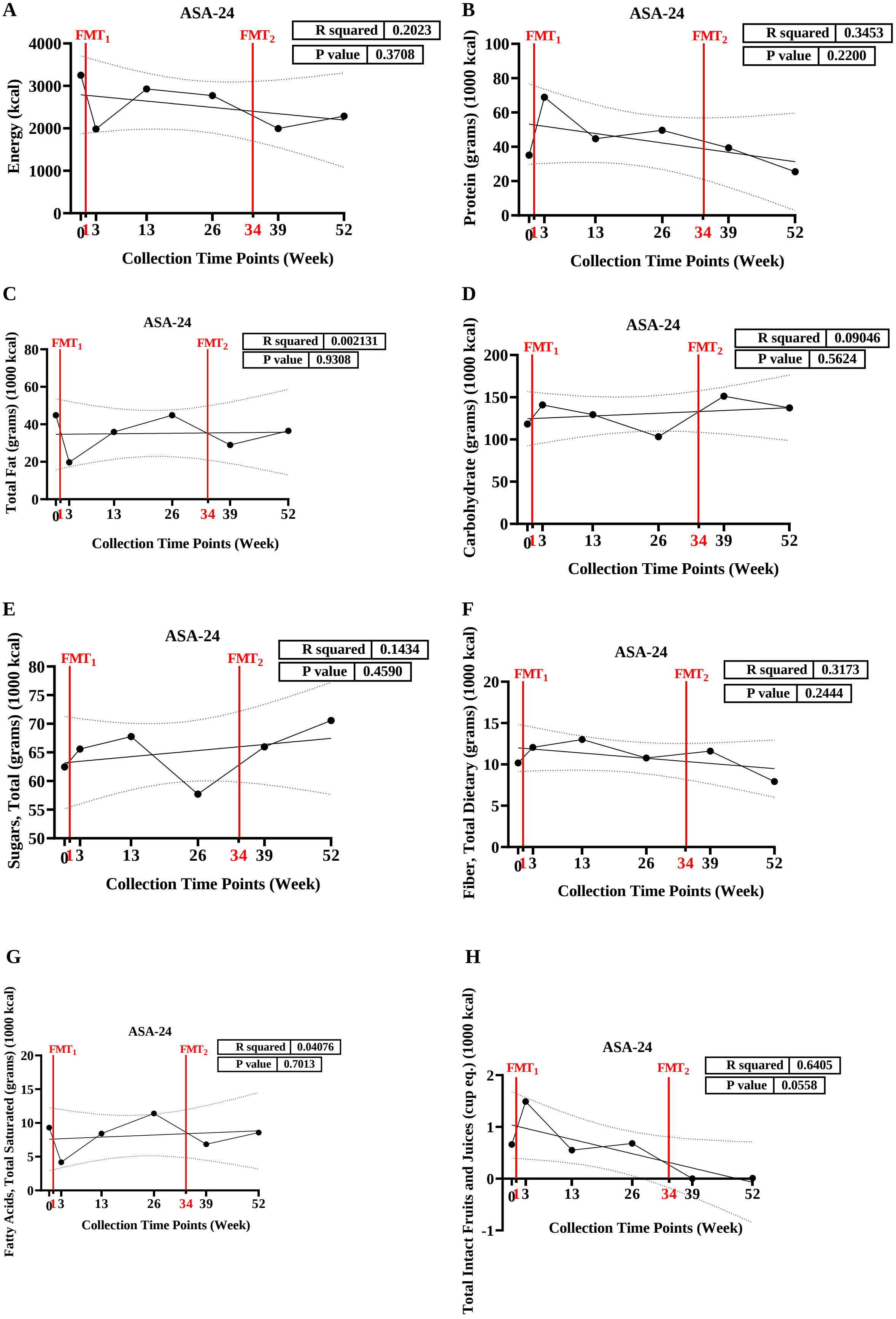
A
ASA-24
R squared 0.2023
P value 0.3708
FMT1
FMT2
Energy (kcal)
0
1000
2000
3000
4000
0 1 3 13 26 34 39 52
Collection Time Points (Week)
B
ASA-24
R squared 0.3453
P value 0.2200
FMT1
FMT2
Protein (grams) (1000 kcal)
0
20
40
60
80
100
0 1 3 13 26 34 39 52
Collection Time Points (Week)
C
ASA-24
R squared 0.002131
P value 0.9308
FMT1
FMT2
Total Fat (grams) (1000 kcal)
0
20
40
60
80
0 1 3 13 26 34 39 52
Collection Time Points (Week)
D
ASA-24
R squared 0.09046
P value 0.5624
FMT1
FMT2
Carbohydrate (grams) (1000 kcal)
0
50
100
150
200
0 1 3 13 26 34 39 52
Collection Time Points (Week)
E
ASA-24
R squared 0.1434
P value 0.4590
FMT1
FMT2
Sugars, Total (grams) (1000 kcal)
50
55
60
65
70
75
80
0 1 3 13 26 34 39 52
Collection Time Points (Week)
F
ASA-24
R squared 0.3173
P value 0.2444
FMT1
FMT2
Fiber, Total Dietary (grams) (1000 kcal)
0
5
10
15
20
0 1 3 13 26 34 39 52
Collection Time Points (Week)
G
ASA-24
R squared 0.04076
P value 0.7013
FMT1
FMT2
Fatty Acids, Total Saturated (grams) (1000 kcal)
0
5
10
15
20
0 1 3 13 26 34 39 52
Collection Time Points (Week)
H
ASA-24
R squared 0.6405
P value 0.0558
FMT1
FMT2
Total Intact Fruits and Juices (cup eq.) (1000 kcal)
-1
0
1
2
0 1 3 13 26 34 39 52
Collection Time Points (Week)

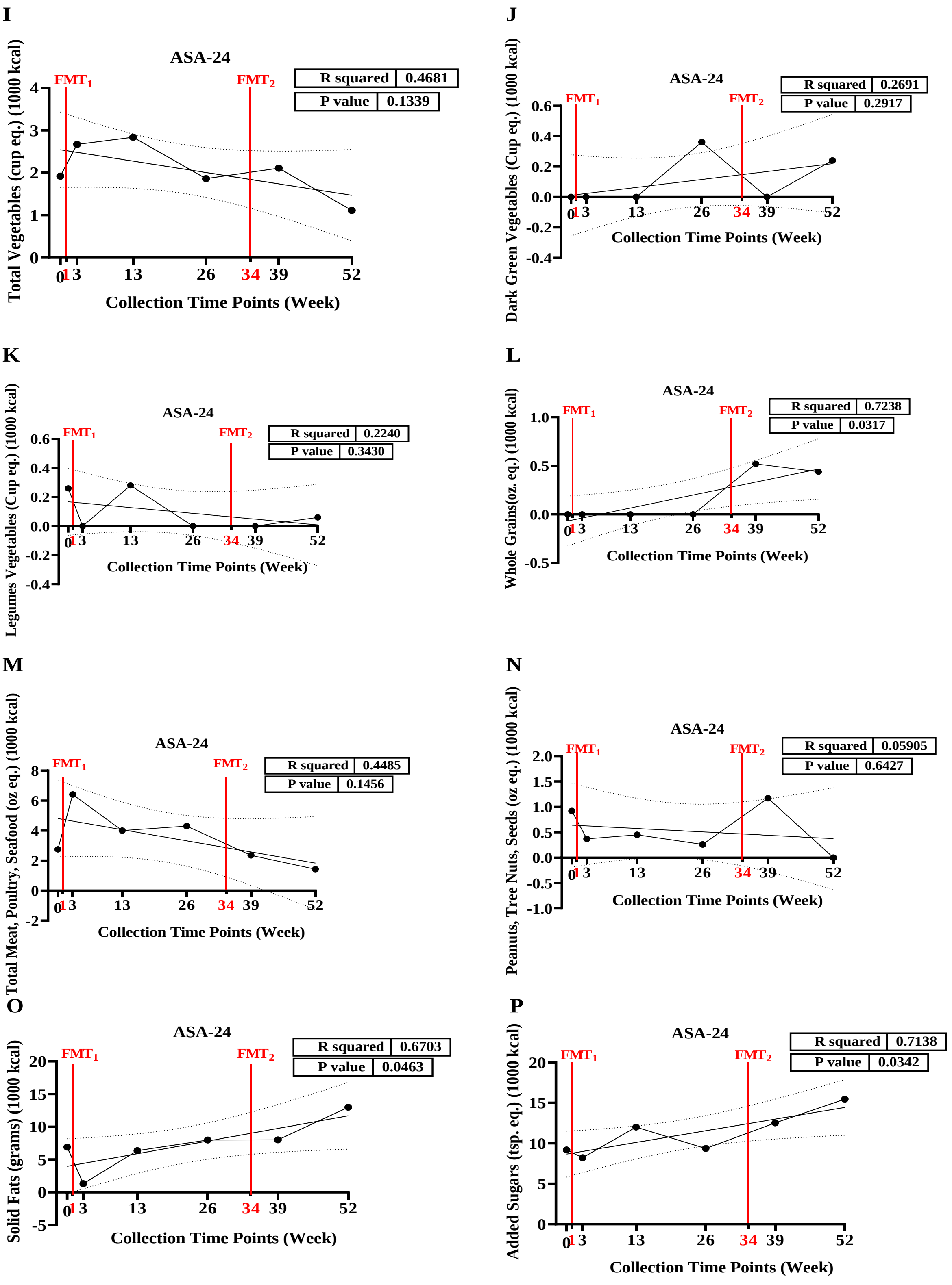


**Supplementary Figure 17.** Linear regression relationships between ASA24® nutrition (per 1000 kcal) variables across six collection time points, in the RRMS FMT subject.

**Food Timing Screener/Food Timing Questionnaire Characteristic Outcomes**

**Supplemental Table 20.** Significant correlation and regression relationships between food timing screener/food timing questionnaire data across time in the RRMS FMT subject.

| FTS / FTQ | Comparison | R value | *P*-value | $R^2$ value | *d* | ES-*r* |
|---|---|---|---|---|---|---|
| Breakfast (Free Days – Saturday and Sunday) | What Time Do You Usually Try to Fall Asleep on these Days (Free Days – Saturday and Sunday) | 0.894 | **0.016** | 0.800 | 34.89 | 0.692 |
| Lunch (Work Days – Monday thru Friday) | Hours Awake (Free Days – Saturday and Sunday) | -0.816 | **0.047** | 0.666 | 84.26 | 0.999 |
| Lunch (Work Days – Monday thru Friday) | Hours Asleep (Free Days – Saturday and Sunday) | 0.816 | **0.047** | 0.666 | 103.14 | 0.999 |
| Social Jet Lag | What Time Do You Usually Wake Up on These Days (Free Days – Saturday and Sunday) | 1.000 | **0.000** | 1.000 | 71.85 | 0.999 |
| Social Jet Lag | Hours Awake (Free Days – Saturday and Sunday) | -0.956 | **0.003** | 0.914 | 28.02 | 0.999 |
| Social Jet Lag | Hours Asleep (Free Days – Saturday and Sunday) | 0.956 | **0.003** | 0.914 | 14.57 | 0.991 |
| Hours Asleep (Free Days – Saturday and Sunday) | Hours Awake (Free Days – Saturday and Sunday) | -1.000 | **0.000** | 1.000 | 15.65 | 0.992 |
| What Time Do You Usually Wake Up on These Days (Free Days – Saturday and Sunday) | Hours Awake (Free Days – Saturday and Sunday) | -0.956 | **0.003** | 0.914 | 52.68 | 0.999 |
| What Time Do You Usually Wake Up on These Days (Free Days – Saturday and Sunday) | Hours Asleep (Free Days – Saturday and Sunday) | 0.956 | **0.003** | 0.914 | 66.13 | 0.999 |

Both Pearson correlation (R) and linear regression ($R^2$) values are shown. FTS = Food Timing Screener; FTQ = Food Timing Questionnaire. Weeks 0, 3, 13, 26, 39, 52 were examined across six time points (n=6). Effect size (ES) calculated using mean and standard deviation between variables. Cohen's (*d*) and effect size (ES-*r*) measures the magnitude of a treatment effect: 0.2 = small effect; 0.5 = moderate effect; 0.8 or > = large effect.

## Experimental or Clinical Correlations and Predictions Across Time

**Supplementary Table 21.** Significant relationships and predictors of blood biomarkers or PROMIS-GI outcomes compared to microbiome and functional genomic pathways across time, in the RRMS FMT subject.

| Blood Marker or PROMIS-GI Outcomes | Comparisons | R value | *P*-value | $R^2$ Value |
|---|---|---|---|---|
| BDNF | ko00620 Pyruvate metabolism | 0.97 | **0.002** | 0.93 |
| BDNF | ko00640 Propanoate metabolism | 0.96 | **0.003** | 0.92 |
| BDNF | ko01120 Microbial metabolism in diverse environments | 0.95 | **0.003** | 0.91 |
| BDNF | ko01200 Carbon metabolism | 0.95 | **0.003** | 0.91 |
| BDNF | ko00603 Glycosphingolipid biosynthesis globo and isoglobo series | 0.95 | **0.004** | 0.90 |
| BDNF | ko01110 Biosynthesis of secondary metabolites | 0.95 | **0.004** | 0.90 |
| BDNF | ko00680 Methane metabolism | 0.95 | **0.004** | 0.90 |
| BDNF | ko01100 Metabolic pathways | 0.94 | **0.005** | 0.90 |
| BDNF | ko00770 Pantothenate and CoA biosynthesis | 0.94 | **0.005** | 0.90 |
| BDNF | ko01130 Biosynthesis of antibiotics | 0.94 | **0.005** | 0.90 |
| BDNF | ko01230 Biosynthesis of amino acids | 0.94 | **0.005** | 0.90 |
| BDNF | ko00061 Fatty acid biosynthesis | 0.94 | **0.005** | 0.90 |
| BDNF | ko01212 Fatty acid metabolism | 0.94 | **0.005** | 0.90 |
| BDNF | ko00130 Ubiquinone and other terpenoid quinone biosynthesis | 0.93 | **0.006** | 0.90 |
| BDNF | Fungi: *Rhodotorula toruloides* | -0.93 | **0.006** | 0.90 |
| BDNF | ko00650 Butanoate metabolism | 0.93 | **0.007** | 0.90 |
| IL-6 | Virus: *Phycodnaviridae* | -0.96 | **0.003** | 0.91 |
| Gas and Bloating | Bacteria: *Enterococcus hirae* | 0.97 | **0.001** | 0.94 |

Both Pearson correlation (R) and linear regression ($R^2$) values are shown. Data was normalized using Log10 transformation. Only $R^2$ values of 0.90 or greater, with a corresponding p-value of $< 0.05$, are depicted. Note: blood markers IL-8 and TNFα did not indicate significant correlations. PROMIS-GI outcomes of belly pain, constipation, and bowel incontinence did not indicate significant correlations. Weeks 0, 3, 13, 26, 39, 52 were examined across time.

**Supplementary Table 22.** Significant relationships and predictors of the three important gait metrics compared to microbiome and clinical variables in the RRMS FMT subject.

| Gait | Comparisons | R value | *P*-value | $R^2$ Value |
|---|---|---|---|---|
| Pelvis Smoothness | Fungi: *Mucoromycota* | -0.99 | **0.001** | 0.99 |
| Pelvis Smoothness | Protein (g) | -0.96 | **0.039** | 0.92 |
| Pelvis Smoothness | Total intact fruits (whole or cut) and fruit juices (cup eq.) | -0.99 | **0.010** | 0.98 |
| Pelvis Smoothness | Beans and peas (legumes) computed as vegetables (cup eq.) | -0.99 | **0.012** | 0.98 |
| Pelvis Smoothness | Grains defined as whole grains and contain the entire grain kernel, the bran, germ, and endosperm (oz. eq.) | -1.00 | **0.004** | 0.99 |
| Step Width | Fungi: *Cordyceps militaris* | 0.98 | **0.022** | 0.96 |
| Stride Time | ko00380 Tryptophan metabolism | -0.98 | **0.021** | 0.96 |
| Stride Time | ko01110 Biosynthesis of secondary metabolites | -0.97 | **0.033** | 0.93 |
| Stride Time | ko00500 Starch and sucrose metabolism | -0.97 | **0.030** | 0.94 |
| Stride Time | ko00130 Ubiquinone and other terpenoid quinone biosynthesis | -0.96 | **0.039** | 0.92 |
| Stride Time | ko00640 Propanoate metabolism | -0.97 | **0.035** | 0.93 |
| Stride Time | ko01130 Biosynthesis of antibiotics | -0.96 | **0.037** | 0.93 |
| Stride Time | ko01120 Microbial metabolism in diverse environments | -0.96 | **0.040** | 0.92 |
| Stride Time | ko01212 Fatty acid metabolism | -0.96 | **0.036** | 0.93 |
| Stride Time | ko01100 Metabolic pathways | -0.96 | **0.043** | 0.92 |
| Stride Time | ko00680 Methane metabolism | -0.96 | **0.043** | 0.92 |
| Stride Time | Virus: *Streptococcus phage YMC2011* | 0.98 | **0.016** | 0.97 |
| Stride Time | ko04142 Lysosome | -0.98 | **0.024** | 0.95 |
| Stride Time | ko03320 PPAR signaling pathway | -0.96 | **0.038** | 0.93 |
| Stride Time | Ko00750 Vitamin B6 Metabolism | -0.96 | **0.039** | 0.92 |
| Stride Time | IL6 | -0.96 | **0.042** | 0.92 |

Both Pearson correlation (R) and linear regression ($R^2$) values are shown. Data was normalized using Log10 transformation. Only $R^2$ values of 0.90 or greater, with a corresponding p-value of $< 0.05$, are depicted. These three gait metrics encompass 100% of all 18 gait metric measurements examined. Weeks 0, 3, 13, 52 were examined across time.

**Supplementary Table 23.** Significant relationships and predictors of the MS walking questionnaire score to functional gene pathways across time in the RRMS subject.

| MS Walking Variables | Comparisons | R value | *P*-value | $R^2$ Value |
|---|---|---|---|---|
| Affected how smoothly you walked | ko00750 Vitamin B6 metabolism | -0.99 | **0.00** | 0.98 |
| Affected how smoothly you walked | ko00944 Flavone & Flavonol Biosynthesis | -0.97 | **0.00** | 0.94 |
| Limited how far able to walk | ko00750 Vitamin B6 metabolism | -0.99 | **0.00** | 0.97 |
| Limited how far able to walk | ko00944 Flavone & Flavonol Biosynthesis | -0.97 | **0.00** | 0.93 |
| Limited how far able to walk | ko04142 Lysosome | -0.95 | **0.00** | 0.91 |
| Slowed down walking | ko04142 Lysosome | -0.97 | **0.00** | 0.95 |
| Slowed down walking | ko00750 Vitamin B6 metabolism | -0.97 | **0.00** | 0.94 |
| Slowed down walking | ko03320 PPAR signaling pathway | -0.96 | **0.00** | 0.92 |
| Slowed down walking | ko00380 Tryptophan metabolism | -0.95 | **0.00** | 0.90 |
| Both Pearson correlation (R) and linear regression ($R^2$) values are shown. Only $R^2$ values of 0.90 or greater, with a corresponding p-value of < 0.05, are depicted. Weeks 0, 3, 13, 26, 39, 52 were examined across time. | | | | |

**Supplementary Table 24.** Significant relationships and predictors of ASA24® dietary variables compared to experimental, microbiome or functional gene pathways across time in the RRMS FMT subject.

| ASA24® Category | Comparisons | R value | *P*-value | $R^2$ Value |
|---|---|---|---|---|
| KCAL | *Weissella cibaria* | 0.94 | **0.005** | 0.89 |
| KCAL | Ascomycota | -0.93 | **0.007** | 0.87 |
| KCAL | *Parastagonospora nodorum* | -0.90 | **0.014** | 0.81 |
| KCAL | BDNF | -0.90 | **0.015** | 0.81 |
| PROT | PF MPS TOTAL | 1.00 | **0.000** | 0.99 |
| TFAT | *Weissella cibaria* | 0.95 | **0.003** | 0.91 |
| TFAT | KCAL | 0.93 | **0.006** | 0.87 |
| TFAT | SFAT | 0.99 | **0.000** | 0.98 |
| CARB | Caudovirales | 0.94 | **0.005** | 0.89 |
| CARB | TNFa | -0.94 | **0.005** | 0.88 |
| CARB | *unidentified phage* | -0.94 | **0.006** | 0.88 |
| CARB | SUGR | 0.90 | **0.016** | 0.80 |
| SUGR | KCAL | 0.94 | **0.005** | 0.89 |
| SUGR | *Weissella cibaria* | 0.92 | **0.008** | 0.85 |
| SUGR | Ascomycota | -0.92 | **0.009** | 0.85 |
| SUGR | *Parastagonospora nodorum* | -0.91 | **0.011** | 0.83 |
| SUGR | TNFa | -0.90 | **0.014** | 0.81 |
| SUGR | CARB | 0.90 | **0.016** | 0.80 |
| SUGR | KCAL | 0.94 | **0.005** | 0.89 |
| FIBE | V TOTAL | 0.95 | **0.004** | 0.89 |

| | | | | |
|---|---|---|---|---|
| SFAT | TFAT | 0.99 | **0.000** | 0.98 |
| SFAT | *Weissella cibaria* | 0.90 | **0.013** | 0.82 |
| F TOTAL | V LEGUMES | 0.97 | **0.001** | 0.95 |
| V TOTAL | FIBE | 0.95 | **0.004** | 0.89 |
| V TOTAL | Acetate Metabolite | 0.93 | **0.008** | 0.86 |
| V DRKGR | *Acidaminococcus intestini* | 0.96 | **0.003** | 0.92 |
| V LEGUMES | F TOTAL | 0.97 | **0.001** | 0.95 |
| PF MPS TOTAL | PROT | 1.00 | **0.000** | 0.99 |
| PF NUTSDS | *Verticillium dahliae* | -0.97 | **0.001** | 0.95 |
| PF NUTSDS | *Streptococcus salivarius* | 0.96 | **0.003** | 0.92 |
| SOLID FATS | *Klebsiella phage 3 LV2017* | -0.97 | **0.001** | 0.94 |
| SOLID FATS | *Bifidobacterium adolescentis* | 0.96 | **0.002** | 0.93 |
| SOLID FATS | *Enterococcus hirae* | -0.95 | **0.004** | 0.90 |
| SOLID FATS | Gas Bloating | -0.92 | **0.008** | 0.85 |
| SOLID FATS | *Methanobrevibacter smithii* | 0.92 | **0.010** | 0.84 |
| SOLID FATS | *Streptococcus parasanguinis* | 0.91 | **0.013** | 0.82 |
| ADD SUGARS | *Bacteroides caccae* | -0.96 | **0.003** | 0.92 |
| ADD SUGARS | Actinobacteria | 0.95 | **0.003** | 0.91 |
| ADD SUGARS | Bacteroidetes | -0.94 | **0.006** | 0.88 |
| ADD SUGARS | *Bacteroides vulgatus* | -0.91 | **0.012** | 0.83 |
| ADD SUGARS | *Ruminococcus sp. SR15* | 0.90 | **0.014** | 0.82 |
| ADD SUGARS | SOLID FATS | 0.90 | **0.014** | 0.81 |

Both Pearson correlation (R) and linear regression ($R^2$) values are shown. Data was normalized using Log10 transformation. Only $R^2$ values of 0.80 or greater, with a corresponding p-value of $< 0.05$, are depicted. Weeks 0, 3, 13, 26, 39, 52 were examined across time.

**REFERENCES**


1. Evans SR. Clinical trial structures. Journal of experimental stroke & translational medicine. 2010;3(1):8-18.

2. Ma Y, Chen H, Lan C, Ren J. Help, hope and hype: ethical considerations of human microbiome research and applications. Protein & cell. 2018;9(5):404-15.

3. Borody TJ, Khoruts A. Fecal microbiota transplantation and emerging applications. Nat Rev Gastroenterol Hepatol. 2011;9(2):88-96.

4. Sharpton TJ. An introduction to the analysis of shotgun metagenomic data. Front Plant Sci. 2014;5:209.

5. Benson DA, Cavanaugh M, Clark K, Karsch-Mizrachi I, Ostell J, Pruitt KD, et al. GenBank. Nucleic Acids Res. 2018;46(D1):D41-D7.

6. Kim D, Song L, Breitwieser FP, Salzberg SL. Centrifuge: rapid and sensitive classification of metagenomic sequences. Genome Res. 2016;26(12):1721-9.

7. Buchfink B, Xie C, Huson DH. Fast and sensitive protein alignment using DIAMOND. Nat Methods. 2015;12(1):59-60.

8. The UniProt C. UniProt: the universal protein knowledgebase. Nucleic Acids Res. 2017;45(D1):D158-D69.

9. Kanehisa M, Furumichi M, Tanabe M, Sato Y, Morishima K. KEGG: new perspectives on genomes, pathways, diseases and drugs. Nucleic Acids Res. 2017;45(D1):D353-D61.

10. Kaur A, Rose DJ, Rumpagaporn P, Patterson JA, Hamaker BR. In vitro batch fecal fermentation comparison of gas and short-chain fatty acid production using "slowly fermentable" dietary fibers. J Food Sci. 2011;76(5):H137-42.

11. Loudon JK, Bell S, Johnston JM. The Clinical Orthopedic Assessment Guide: Human Kinetics; 1998.

12. Espy DD, Yang F, Bhatt T, Pai YC. Independent influence of gait speed and step length on stability and fall risk. Gait & posture. 2010;32(3):378-82.

13. Tjhai C, O'Keefe K. Using Step Size and Lower Limb Segment Orientation from Multiple Low-Cost Wearable Inertial/Magnetic Sensors for Pedestrian Navigation. Sensors. 2019;19(14).

14. Danion F, Varraine E, Bonnard M, Pailhous J. Stride variability in human gait: the effect of stride frequency and stride length. Gait & posture. 2003;18(1):69-77.

15. Tudor-Locke C, Rowe DA. Using cadence to study free-living ambulatory behaviour. Sports medicine. 2012;42(5):381-98.

16. Zatsiorky VM, Werner SL, Kaimin MA. Basic kinematics of walking. Step length and step frequency. A review. The Journal of sports medicine and physical fitness. 1994;34(2):109-34.

17. Owings TM, Grabiner MD. Step width variability, but not step length variability or step time variability, discriminates gait of healthy young and older adults during treadmill locomotion. Journal of biomechanics. 2004;37(6):935-8.

18. R. Barry Dale. 21 - Clinical Gait Assessment. James R. Andrews GLH, Kevin E. Wilk, editor: Physical Rehabilitation of the Injured Athlete (Fourth Edition); 2012. 464-79 p.

19. Lewis CL, Laudicina NM, Khuu A, Loverro KL. The Human Pelvis: Variation in Structure and Function During Gait. Anatomical record. 2017;300(4):633-42.

20. Gulde P, Hermsdorfer J. Smoothness Metrics in Complex Movement Tasks. Frontiers in neurology. 2018;9:615.

21. Brach JS, McGurl D, Wert D, Vanswearingen JM, Perera S, Cham R, et al. Validation of a measure of smoothness of walking. J Gerontol A Biol Sci Med Sci. 2011;66(1):136-41.

22. Menz HB, Lord SR, Fitzpatrick RC. Acceleration patterns of the head and pelvis when walking are associated with risk of falling in community-dwelling older people. J Gerontol A Biol Sci Med Sci. 2003;58(5):M446-52.

23. Hobart JC, Riazi A, Lamping DL, Fitzpatrick R, Thompson AJ. Measuring the impact of MS on walking ability: the 12-Item MS Walking Scale (MSWS-12). Neurology. 2003;60(1):31-6.

24. Spiegel BM, Hays RD, Bolus R, Melmed GY, Chang L, Whitman C, et al. Development of the NIH Patient-Reported Outcomes Measurement Information System (PROMIS) gastrointestinal symptom scales. Am J Gastroenterol. 2014;109(11):1804-14.

25. Moshfegh AJ, Rhodes DG, Baer DJ, Murayi T, Clemens JC, Rumpler WV, et al. The US Department of Agriculture Automated Multiple-Pass Method reduces bias in the collection of energy intakes. Am J Clin Nutr. 2008;88(2):324-32.

26. Kirkpatrick SI, Subar AF, Douglass D, Zimmerman TP, Thompson FE, Kahle LL, et al. Performance of the Automated Self-Administered 24-hour Recall relative to a measure of true intakes and to an interviewer-administered 24-h recall. Am J Clin Nutr. 2014;100(1):233-40.

27. Subar AF, Kirkpatrick SI, Mittl B, Zimmerman TP, Thompson FE, Bingley C, et al. The Automated Self-Administered 24-hour dietary recall (ASA24): a resource for researchers, clinicians, and educators from the National Cancer Institute. Journal of the Academy of Nutrition and Dietetics. 2012;112(8):1134-7.

28. Kirkpatrick SI, Potischman N, Dodd KW, Douglass D, Zimmerman TP, Kahle LL, et al. The Use of Digital Images in 24-Hour Recalls May Lead to Less Misestimation of Portion Size Compared with Traditional Interviewer-Administered Recalls. J Nutr. 2016;146(12):2567-73.

29. Thompson FE, Dixit-Joshi S, Potischman N, Dodd KW, Kirkpatrick SI, Kushi LH, et al. Comparison of Interviewer-Administered and Automated Self-Administered 24-Hour Dietary Recalls in 3 Diverse Integrated Health Systems. Am J Epidemiol. 2015;181(12):970-8.

30. Chakradeo P. Validity and Reliability of the Food Timing Questionnaire (FTQ) and Food Timing Screener (FTS). ProQuest: Rush University; 2018.

31. Oksanen J, Blanchet FG, Kindt R, Legendre P, Minchin PR, O'Hara R, et al. Package 'vegan'. Community ecology package. 2 ed2016.

32. Ritchie ME, Phipson B, Wu D, Hu Y, Law CW, Shi W, et al. limma powers differential expression analyses for RNA-sequencing and microarray studies. Nucleic Acids Res. 2015;43(7):e47.

33. Jangi S, Gandhi R, Cox LM, Li N, von Glehn F, Yan R, et al. Alterations of the human gut microbiome in multiple sclerosis. Nat Commun. 2016;7:12015.
